# ASAFind 2.0: multi‐class protein targeting prediction for diatoms and algae with complex plastids

**DOI:** 10.1111/tpj.70138

**Published:** 2025-06-04

**Authors:** Ansgar Gruber, Marta Vohnoutová, Cedar McKay, Gabrielle Rocap, Miroslav Oborník

**Affiliations:** ^1^ Biology Centre Institute of Parasitology, Czech Academy of Sciences České Budějovice Czech Republic; ^2^ Faculty of Science University of South Bohemia České Budějovice Czech Republic; ^3^ School of Oceanography University of Washington Seattle Washington USA

**Keywords:** diatoms, organelle, genome annotation, secretory pathway, chloroplast, mitochondria, periplastidic compartment, protein transport, evolution, gene transfer, technical advance

## Abstract

Plastids of diatoms and related algae with complex plastids of red algal origin are surrounded by four membranes, which also define the periplastidic compartment (PPC), the space between the second and third membranes. Metabolic reactions as well as cell biological processes take place in the PPC; however, genome‐wide predictions of the proteins targeted to this compartment were so far based on manual annotation work. Using published experimental protein localizations as reference data, we developed the first automatic prediction method for PPC proteins, which we included as a new feature in an updated version of the plastid protein predictor ASAFind. With our method, at least a subset of the PPC proteins can be predicted with high specificity, with an estimate of at least 81 proteins (0.7% of the predicted proteome) targeted to the PPC in the model diatom *Phaeodactylum tricornutum*. The proportion of PPC proteins varies, since 180 PPC proteins (1.3% of the predicted proteome) were predicted in the genome of the diatom *Thalassiosira pseudonana.* The new ASAFind version can also generate a newly designed graphical output that visualizes the contribution of each position in the sequence to the score and accepts the output of the recent versions of SignalP (5.0) and TargetP (2.0) as input data. Furthermore, we release a script to calculate custom scoring matrices that can be used for predictions in a simplified score cut‐off mode. This allows for adjustments of the method to other groups of algae.

## Significance Statement

Many photosynthetic eukaryotes possess complex plastids, which are surrounded by four membranes, and by a space known as the periplastidic compartment (PPC). ASAFind is a software for the prediction of nucleus‐encoded plastid proteins in organisms with complex plastids of red algal origin, like diatoms. Here we present a new version of ASAFind, which includes optional prediction of PPC targeted proteins, and which visualizes the scores in a newly designed graphical output.

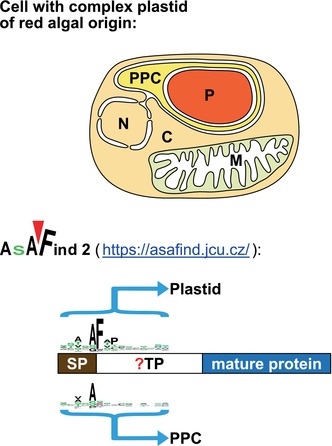

## INTRODUCTION

Eukaryotic cells are characterized by the presence of membrane‐bound organelles, which fulfill distinct functions in the cell. The location of a protein in the cell has important consequences for its function. Therefore, *in silico* prediction software for intracellular protein locations became important in analyses of biological sequence data, on (meta‐) genome or (meta‐) transcriptome levels (Emanuelsson et al., 2007; Nielsen et al., 2019).

In photosynthetic eukaryotes, photosynthesis as well as other important metabolic pathways take place in the plastids (chloroplasts) (Gould et al., 2008). These organelles are of endosymbiotic origin, they evolved from cyanobacteria, which were taken up by a eukaryotic cell, and transformed into organelles. However, in the majority of photosynthetic organisms, plastid evolution was more convoluted and involved one or several eukaryote–eukaryote endosymbioses (Archibald, 2009, 2015; Gruber & Oborník, 2024; Oborník, 2019). This led to a variety of cellular topologies that can be found in the resulting organisms with these, so‐called, “complex” plastids (Gould et al., 2008; Kroth, 2002). Also the mechanisms and signals for protein targeting differ between cells with complex plastids, and cells that contain primary plastids, that directly trace back to cyanobacteria (Gould et al., 2008; Gruber & Kroth, 2024; Gruber & Medlin, 2023; Kroth, 2002; Novák Vanclová et al., 2020; Patron & Waller, 2007).

Complex plastids are characterized by the presence of an additional cell compartment, the periplastidic compartment (PPC). The PPC corresponds to the former cytosol of the eukaryotic endosymbiont, which gave rise to the plastid. Most knowledge about the PPC comes from cryptophyte algae, which harbor a residual nucleus of the former red algal endosymbiont, called the nucleomorph, inside the PPC (Archibald, 2009; Gould et al., 2008). From the genes encoded on the nucleomorph, it can be concluded that cell biological and metabolic processes take place in the PPC (Curtis et al., 2012). In diatoms, which also contain complex plastids of red algal origin, the function of the PPC is more elusive (Gruber et al., 2009; Weber et al., 2009), although the PPC is probably an important player in the metabolic network of the cell (Ewe et al., 2018; Flori et al., 2016; Moog, 2018; Tachibana et al., 2011; Yu et al., 2022). Ultrastructural investigations of the PPC furthermore highly suggest that specific proteins must be in charge of the observed functions/features, like for example, the maintenance of an internal vesicular network (Flori et al., 2016), and many components of the plastid protein transport machinery are thought, or have been shown to be targeted to the PPC (Gruber & Kroth, 2024; Moog et al., 2011). Despite the fact that many PPC proteins in diatoms are known or have been suggested (Moog et al., 2011), currently there is no method for the prediction of PPC proteins from genome data.

Organisms with rhodophyte‐derived complex plastids are particularly diverse and distributed worldwide in marine and freshwater habitats (Archibald, 2009; de Vargas et al., 2015). While there are clear similarities pointing to a common origin of the plastids in these organisms, it is currently not clear how exactly they were acquired by the individual phylogenetic groups in which they are found (Gruber & Oborník, 2024). Organisms with rhodophyte‐derived complex plastids are either polyphyletic, or are at least separated by more than hundreds of millions of years of evolution. This is reflected by a high diversity of targeting mechanisms, targeting signals, and organelle proteome compositions in these organisms (Schwartzbach et al., 2024). Rhodophyte‐derived complex plastids can be found in Stramenopiles (including diatoms), alveolates, haptophytes, and cryptophytes; in these groups of algae, protein targeting to the plastids starts with the passage of the pre‐protein through the endoplasmic reticulum (ER) (Schwartzbach et al. 2024; Gould et al., 2008; Kroth, 2002). Nucleus‐encoded plastid proteins consequently are translated on membrane‐bound ribosomes and possess cleavable signal peptides that are exchangeable with ER signal peptides. The signal peptide is followed by a transit peptide, with similar features as transit peptides of proteins targeted to primary plastids, forming a bipartite targeting signal (Apt et al., 2002; Kroth, 2002; Patron & Waller, 2007). For this reason, plastid protein prediction in organisms with complex plastids cannot be achieved with the prediction tools developed for plastid proteins of higher plants and algae with primary plastids (Gruber & Kroth, 2014, 2017; Moog, 2018).

Two dedicated plastid protein prediction tools have been developed for heterokont algae, HECTAR (Gschloessl et al., 2008) and ASAFind (Gruber et al., 2015). HECTAR performs a meta‐analysis of various methods for signal‐ and transit peptide prediction, combined with a sequence motif search at the signal peptide cleavage site for the identification of plastid proteins (Gschloessl et al., 2008). A particular strength of HECTAR is the integrated identification of signal peptides and mitochondrial transit peptides; however, due to HECTAR being closed‐source software, the internal weighting of parameters for the HECTAR prediction is not directly comprehensible for the user, and therefore tweaking or tinkering with the method is difficult.

ASAFind is an open source software, which uses the protein sequence plus user‐supplied signal peptide predictions as input data, and employs a scoring matrix of proteins that are exclusively from diatoms (Gruber et al., 2015). The initially recommended signal peptide prediction input for ASAFind was from SignalP 3.0 NN (Bendtsen et al., 2004), while SignalP 4 (Petersen et al., 2011) is also accepted as input by ASAFind. Unlike HECTAR, ASAFind always processes the results of just one signal peptide predictor. Due to the conservation of the signal peptide cleavage site motif across various groups of organisms with red algal‐derived complex plastids (Patron & Waller, 2007), ASAFind can also be used with input data from other groups of algae in which the motif is found, albeit with a lower performance compared with diatoms (Curtis et al., 2012; Gruber et al., 2015). Among the organisms with complex plastids of red algal origin, the signal peptide cleavage site motif appears to be more weakly conserved in peridinin‐containing dinoflagellates (Patron et al., 2005; Patron & Waller, 2007), and in the closest photosynthetic relatives to apicomplexan parasites, the chromerid algae *Chromera velia* and *Vitrella brassicaformis* (Füssy et al., 2019). Recently, for the two chromerid algae, it has been shown that modification of the transit peptide scoring matrix, to one that has been compiled from the target organism, increases the prediction performance in the two chromerids (Füssy et al., 2019).

Here, we present a new version of ASAFind, which (i) includes predictions of PPC targeted proteins, (ii) allows for an easy calculation of custom scoring matrices for target organisms with specific requirements, (iii) accepts the results of newly published signal peptide predictors as input data, and (iv) generates a graphical output that visualizes the contribution of each position in the sequence to the overall score.

ASAFind 2.0 is the first prediction software for nucleus‐encoded PPC targeted proteins in diatoms, which are recognized by scoring the transit peptide region of the bipartite targeting signals. Scoring matrices for diatoms are included as default, and in addition, custom scoring matrices for other organisms with complex plastids can be generated to optimize the method for other organisms.

## RESULTS AND DISCUSSION

### General improvements

ASAFind builds its prediction on the identification of a conserved sequence motif, which surrounds the signal peptide cleavage site, as first described by Kilian and Kroth (2005). The sequence conservation is partly due to the presence of a signal peptide cleavage site motif (see −1 and −3S rule for signal peptides, von Heijne, 1983), and partly due to the presence of a phenylalanine (or similar) residue at the N‐terminus of the transit peptide (Gruber et al., 2007; Kilian & Kroth, 2005), a sequence feature that is also known from plastid transit peptides in red‐ and glaucophyte algae and from most groups of algae with complex plastids of red algal origin (Patron & Waller, 2007).

ASAFind uses a scoring matrix that was calculated from diatom plastid proteins (Gruber et al., 2015), and uses the information content of each position as well as the frequency of the residue found in this position (according to the procedure described by Schneider & Stephens, 1990). ASAFind identifies the motif by collecting position‐specific scores while scanning the region surrounding the signal peptide cleavage site that is provided as input data; the highest scoring sequence window is then used for the calculation of the transit peptide score (Gruber et al., 2015). Accepted input data for ASAFind 1 are the output of SignalP 3.0 NN (Bendtsen et al., 2004) and SignalP 4 (Petersen et al., 2011). Recently, a new version of SignalP (SignalP 5.0, Almagro Armenteros, Tsirigos, et al., 2019) has been published, and also a new version of the multi‐class protein targeting predictor TargetP (TargetP 2.0) has been developed that uses a similar approach as SignalP 5.0 and reaches similar prediction performance (Almagro Armenteros, Salvatore, et al., 2019). The combination of the new SignalP and TargetP versions with ASAFind turned out to be advantageous for the performance of ASAFind (Gruber et al., 2020), and TargetP 2.0 also performs well in the prediction of mitochondrial proteins of diatoms (Gruber et al., 2020). For the new version of ASAFind, we therefore decided to include the option of using SignalP 5.0 and TargetP 2.0 output for the plastid protein prediction (Appendix S1), and based on the statistical performance of the signal peptide predictors (Gruber et al., 2020), we recommend using ASAFind 2 in conjunction with TargetP 2.

ASAFind also proved useful for the prediction of plastid proteins in other groups of algae with complex plastids (Curtis et al., 2012; Dorrell et al., 2017; Füssy et al., 2019; Wetherbee et al., 2019), however, the prediction performance is lower than in diatoms (Curtis et al., 2012; Füssy et al., 2019), but in many cases cannot be tested and therefore remains unknown. Also experimental protein localizations are challenging to compare between organisms (Richtová et al., 2021). To facilitate the development of protein targeting predictions in other organisms than diatoms, we included the feature of using custom scoring matrices in the new ASAFind 2 (Appendix S1). A similar approach has been used by Füssy et al. (2019), who found that it improves the prediction performance for *C. velia* and *V. brassicaformis* plastid‐targeted proteins. We also included a custom scoring matrix generator with the new ASAFind, which calculates a scoring matrix from a FASTA file of training proteins (Appendix S1). In the new custom scoring matrix generator, also a small sample correction as suggested by Schneider et al. (1986) and Schneider and Stephens (1990) is applied, and we updated the original diatom plastid protein scoring matrix used by ASAFind by default, accordingly. Because the score cut‐off for the “plastid, high confidence” category of ASAFind is supplemented with a hard‐wired check for the presence of phenylalanine, tyrosine, tryptophan, or leucine at the +1 position of the transit peptide (see figure 3 of Gruber et al., 2015), which is incompatible with the method sugested by Füssy et al. (2019), the new ASAFind also offers a simple score cut‐off mode, in which no distinction between confidence levels is made. This simple score cut‐off mode facilitates adjustment of ASAFind to other organisms and scoring matrices, and allows for an exact reproduction of the method described by Füssy et al. (2019).

**Figure 1:**
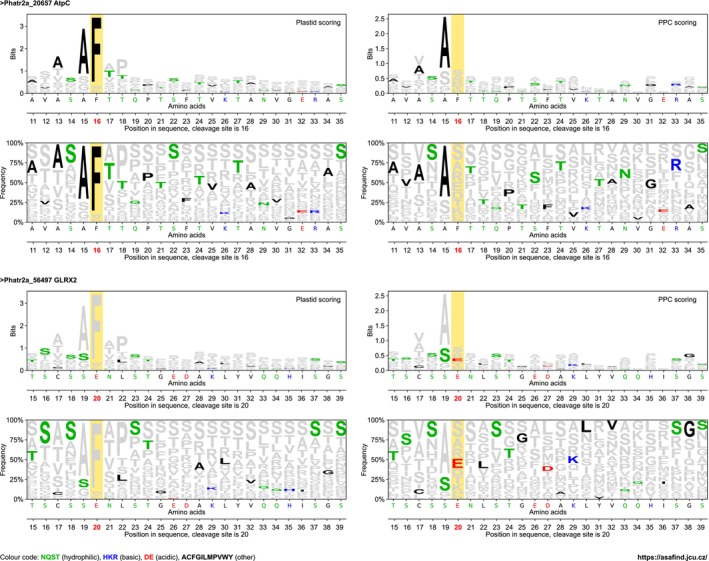
Exemplar graphical output generated by ASAFind 2. The actual sequence is highlighted against the respective scoring matrix, visualizing the contribution of each position in the sequence to the overall score. AtpC is plastid targeted, GLRX2 is PPC targeted, and the sequences are shown against both scoring matrices for comparison. PPC, periplastidic compartment.

For comparison, we also assembled a cryptophyte plastid protein scoring matrix that was obtained by BLAST, with the original diatom scoring matrix as a query against all cryptophyte sequences available at NCBI. For the BLAST search, only the mature parts of the proteins were used as queries in order not to self‐amplify the similarity of the expected targeting signals. The resulting set of sequences was then narrowed down to only three hits per sequence, avoiding overrepresentation of common and conserved proteins (Appendix S9). The resulting scoring matrix (Appendices S9 and S10) can readily be used with ASAFind 2; however, it should be noted that currently the statistical performance of this method is unknown.

Analogously to the plastid scoring matrices, ASAFind 2 also accepts custom scoring matrices for PPC protein predictions, which might help to further improve PPC protein predictions or to adjust the method to other groups of organisms than diatoms.

For manual inspection of the targeting result, we designed a graphical output that plots the residues of the tested sequence against the background of the scoring matrix (Figure 1). This visualizes the contribution of each position to the overall score, and crucial positions could, for example, be identified by *in silico* editing of the sequence and comparison of the results of the original versus the edited sequence. This procedure can be especially useful for the design of protein targeting experiments.

**Figure 2:**
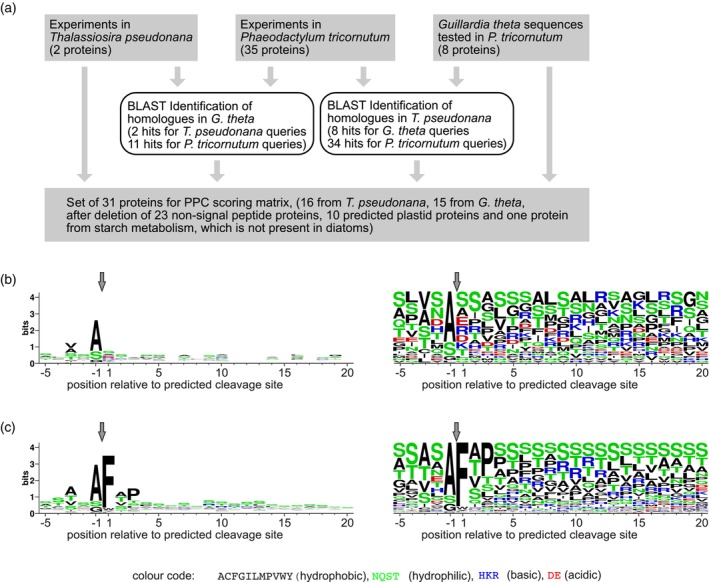
Scoring matrix for PPC protein prediction. (a) Composition of the sequence set. (b) Sequence logo (left) and frequency plot (right) of the 31 sequence set used to calculate the PPC scoring matrix. (c) Plastid protein scoring matrix (166 sequences, reproduced from Gruber et al., 2015). PPC, periplastidic compartment.

### Periplastidic compartment protein prediction

In cells with complex plastids, protein targeting to the PPC, in the same way as plastid protein targeting, starts with ER import of the protein (Gould, Sommer, Kroth, et al., 2006; Gruber & Kroth, 2024). While the PPC is the destination for PPC targeted proteins, also the plastid‐targeted proteins pass through the PPC during transport (Gould et al., 2008; Gruber & Kroth, 2024). The targeting signals of PPC proteins are very similar to plastid targeting signals; in GFP fusion experiments, a single amino acid exchange resulted in re‐targeting of a protein to the plastid (Gould, Sommer, Kroth, et al., 2006). Comparing the bipartite targeting pre‐sequences of plastid and PPC proteins, the clearest difference seems to be the lack of a conserved phenylalanine (or similar) residue in the +1 position of the transit peptide (Gould, Sommer, Kroth, et al., 2006; Gruber et al., 2009; Moog et al., 2011; Weber et al., 2009). We therefore decided to include scoring of PPC transit peptides to ASAFind 2 (Appendix S1), and collected a set of PPC proteins that has no overlap to the reference set for testing the method (Figure 2; Appendices S2 and S3; “Experimental procedures” section for details). As expected, in this set of sequences, phenylalanine or similar residues are not observed in the +1 position of the transit peptide, while the signal peptide cleavage site motif upstream of the transit peptide is conserved in a similar way as in the plastid targeting pre‐sequences (Figure 2). Furthermore, another difference between plastid and PPC targeting pre‐sequences became apparent; in plastid proteins, the first 15 positions of transit peptides are almost devoid of negatively charged residues, while in PPC proteins, negatively charged residues are present and evenly distributed across the whole 25 position sequence window (Figure 2). This is in line with the experimental finding that positive net charges are required for protein transport to the plastid, but not to the PPC (Felsner et al., 2010). A third difference is that in PPC proteins, both, positively and negatively charged residues, are evenly distributed across the whole tested transit peptide length, while in plastid proteins, the first three residues downstream of the signal peptide cleavage site are devoid of charged residues (Figure 2).

**Figure 3:**
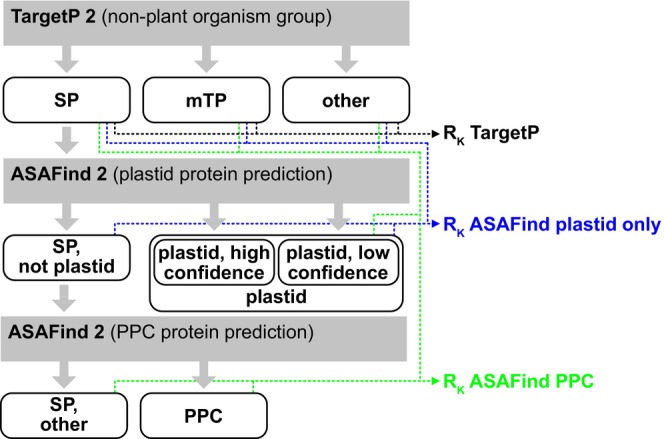
Decision tree of combinations of TargetP 2 and ASAFind 2, and corresponding correlation coefficients (*R*_K_, see Table 1).

Optional scoring of sequences for PPC targeting was implemented into ASAFind 2, analogously to the collection of a 20‐residue plastid transit peptide score starting from the position that gave the highest cleavage site score during the sliding window cleavage site motif identification (described in more detail in Gruber et al., 2015). The complete method follows the decision tree shown in Figure 3. With this sequential procedure, the PPC prediction module profits from the high specificity and sensitivity of the preceding plastid protein prediction.

In order to evaluate the performance of the plastid and PPC protein predictions, we updated the reference set of experimentally localized *Phaeodactylum tricornutum* proteins (Gruber et al., 2015) with newly published experimental localizations of proteins (Appendix S4, details in “Experimental procedures” section). The new reference set consists of 262 proteins, which could unambiguously be classified into the following categories: plastid, 71 sequences; PPC, 44 sequences; other signal peptide containing proteins, 92 sequences; mitochondrial proteins, 26 sequences; other (no targeting pre‐sequence), 29 sequences. This set is not only much larger than the previous version used by Gruber et al. (2015) (132 sequences, 55 plastid, 77 non‐plastid, positive: negative ratio for plastid proteins is 0.71), but also more balanced between the categories (more non‐plastid and non‐PPC proteins, positive: negative ratios are 0.37 for plastid proteins, 0.2 for PPC proteins, 0.11 for mitochondrial proteins, and 3.76 for proteins with signal peptides).

According to the classifications given in Figure 3, we calculated multi‐class correlation coefficients (*R*_K_), which were positive and above 0.5 in all cases (Table 1, calculated from Appendix S5; *R*_K_ can range between −1 and 1, 0 is indicative of a random assignment, 1 represents a perfect prediction). The addition of the plastid category by ASAFind 2 led to an increased overall *R*_K_ compared with the three categories already predicted by TargetP 2 (Table 1). However, this is not the case when the PPC protein prediction is included, underlining that the PPC protein prediction is less accurate than the predictions for the other categories (Table 1). Comparing the per‐category parameters precision and recall (or sensitivity) (Table 2), it becomes evident that the PPC protein predictions are highly precise (79% of positive predictions are correct), but not very sensitive (only 25% of the PPC proteins are detected), and when comparing to experimentally determined organellar proteomes of *Thalassiosira pseudana*, it appears that the distinction between mitochondrial and PPC proteins is particularly good (Appendix S11a). However, the low sensitivity of the PPC protein prediction provokes the question of whether PPC proteins are indeed a homologous group, or if multiple distinct targeting signals lead to the assembly of a seemingly homologous set of proteins. Furthermore, the presence of experimental false‐positives (not from prediction, but from experimental artifacts) could also contribute to explaining this finding. It is therefore interesting that most of the PPC proteins from the reference set were experimentally localized in GFP fusion experiments (Appendix S4), following the observation of a phenotype in the transformed cell lines that has been described as “blob”‐like structure (BLS). This BLS phenotype has first been observed in cells expressing GFP fused to mutated plastid targeting pre‐sequences (Kilian & Kroth, 2005), and is indistinguishable from the phenotype observed in cells expressing GFP fused to native PPC targeting pre‐sequences (Gould, Sommer, Kroth, et al., 2006; Gruber et al., 2009). A very similar phenotype of intracellular GFP distribution has been observed in *Thalassiosira pseudonana* when pre‐sequences of cell wall proteins were fused to the GFP reporter (Poulsen et al., 2013). Moog et al. (2011) also reported on the challenges of manually predicting PPC proteins and on a substantial number of discrepancies between expected and observed locations. In our *P. tricornutum* reference dataset, conflicting experimental results between the BLS phenotype in GFP fusion experiments and extracellular detection of the protein in a proteomics study were observed in two cases (Appendix S4). In addition to this, ambiguous/conflicting results between BLS and ER experimental locations also occurred two times (Appendix S4). Recently, also proteins that are dually targeted to plastid and PPC in *P. tricornutum* have been described (Yang et al., 2025). Therefore, classifications of proteins as PPC proteins should generally be seen with caution in the case of predictions, as well as in the case of experimental results. Given the high precision of our current PPC protein prediction and the biological interest in the PPC, we decided to release the PPC protein prediction feature despite its low sensitivity. Also for PPC predictions, the graphical output mentioned above can be generated, and in this case, all predicted plastid and PPC proteins are plotted against both scoring matrices, for direct comparison (see Figure 1 for examples).

**Table 1:** Correlation coefficients (*R*_K_, according to Gorodkin, 2004) of multi‐class targeting prediction methods (see Fig. 3), tested with the reference set of experimentally localized *Phaeodactylum tricornutum* proteins (262 sequences, Appendix S7)

Method	*R*_K_	Number of categories
ASAFind 2.0 plastid only (with TargetP 2.0)	0.61	4
ASAFind 2.0 PPC (with TargetP 2.0)	0.52	5
TargetP 2.0^a^	0.57	3
HECTAR^b^	0.59	4

PPC, periplastidic compartment.

^a^ Non‐plant organism group.

^b^ “Signal peptide” and “signal anchor” categories combined, see “Experimental procedures” section for details.

**Table 2:** Per‐class prediction performance for all classes that can be predicted by ASAFind 2 in conjunction with TargetP 2.0, and in comparison to HECTAR; tested with the set of experimentally localized *Phaeodactylum tricornutum* proteins (262 proteins, Appendix S7)

Category	Method	Precision	Recall	F_1_ score	Support
		$\frac{\mathrm{TP}}{\mathrm{TP}+\mathrm{FP}}$	$\frac{\mathrm{TP}}{\mathrm{TP}+\mathrm{FN}}$	$\frac{2\;\text{precision}\times \text{recall}}{\text{precision}+\text{recall}}$	P
Plastid, low or high confidence	TargetP 2.0/ASAFind 2	0.77	0.89	0.82	71
Plastid, high confidence only	TargetP 2.0/ASAFind 2	0.91	0.72	0.80	71
SP, not plastid	TargetP 2.0/ASAFind 2	0.94	0.50	0.65	136
SP, not plastid, not PPC	TargetP 2.0/ASAFind 2	0.64	0.40	0.49	92
PPC	TargetP 2.0/ASAFind 2	0.79	0.25	0.38	44
SP	TargetP 2.0	0.98	0.73	0.84	207
mTP	TargetP 2.0	0.80	0.77	0.78	26
other	TargetP 2.0	0.34	0.97	0.50	29
Chloroplast	HECTAR	0.85	0.63	0.73	71
Mitochondrion	HECTAR	0.82	0.69	0.75	26
Secretory pathway^a^	HECTAR	0.78	0.72	0.75	136
Other	HECTAR	0.46	0.97	0.62	29

Precision (positive predictive value), recall (sensitivity) and F_1_ scores were calculated according to the given formulas, support (positives) indicates the number of proteins from each category in the reference set.

FN, false‐negatives; FP, false‐positives; mTP, mitochondrial transit peptide; P, positives; PPC, periplastidic compartment; SP, signal peptide; TP, true‐positives.

^a^ “Signal peptide” and “signal anchor” categories combined, see “Experimental procedures” section for details.

The method allows prediction of at least a subset of the PPC proteins from genome data with high specificity. In the case of the widely investigated model diatoms *P. tricornutum* and *T. pseudonana*, this allows for the estimation that there are at least 81 *P. tricornutum* PPC proteins and at least 180 *T. pseudonana* PPC proteins (Table 3; Appendix S11b; raw data in Appendices S6 and S7). This discrepancy can only partially be explained by genome size (10 814 predicted proteins in *P. tricornutum*, vs. 13 344 predicted proteins in *T. pseudonana*, according to optimized gene catalogs published by Gruber et al., 2015), and surprisingly, there is no overlap between the represented KEEG categories in the predicted PPC proteins between the two organisms (Appendix S11b). However, this might indeed point to biological differences, as there is a number of experimental studies on selected proteins of interest in which protein locations with respect to PPC proteins differ between *P. tricornutum* and *T. pseudonana* (Ewe et al., 2018; Gruber et al., 2009; Samukawa et al., 2014; Tanaka et al., 2014; Weber et al., 2009). In contrast to this discrepancy in the number of predicted PPC proteins, the numbers of predicted proteins for plastids (1 315 in *P. tricornutum*, 1 338 in *T. pseudonana*) and mitochondria (545 in *P. tricornutum*, 475 in *T. pseudonana*) are more similar between the two species (Table 3).

**Table 3:** Genome‐wide ASAFind 2 predictions of organelle targeted proteins in the diatoms *Phaeodactylum tricornutum* and *Thalassiosira pseudonana*, based on N‐terminally optimized gene catalogs (Gruber et al., 2015) and results of TargtP 2.0 (Almagro Armenteros, Salvatore, et al., 2019), see text for details

	*Phaeodactylum tricornutum*	*Thalassiosira pseudonana*
Total number of predicted proteins	10 814	13 344
Proteins with signal peptide	2 130 (19.7)	2 413 (18.1)
Plastid proteins (high or low confidence)	1 315 (12.2)	1 338 (10.0)
Plastid proteins (high confidence only)	789 (7.3)	933 (7.0)
PPC proteins	81 (0.7)	180 (1.3)
Other secretory pathway proteins	734 (6.8)	895 (6.7)
Proteins with mTP	545 (5.0)	475 (3.6)
Other proteins	8 139 (75.3)	10 456 (78.4)

Absolute numbers are given, with percentage of predicted proteome in parentheses.

mTP, mitochondrial transit peptide; PPC, periplastidic compartment.

### Conclusions

Taken together, ASAFind 2, with the forwarding of mitochondrial predictions from TargetP 2, allows for multi‐class protein targeting predictions in diatoms and related algae. The graphical output is helpful for manual checking of the prediction results and for the design of experimental studies. The scoring matrices of ASAFind 2 can be adjusted and tweaked for specific groups of organisms. Furthermore, the optional PPC protein predictions will hopefully enable experimental studies on the function of this elusive cellular compartment.

## EXPERIMENTAL PROCEDURES

ASAFind 2 was developed from the original ASAFind code published in Gruber et al. (2015). Work on the ASAFind update started with ASAFind.py version 1.1.7. The current version at the time of publication is included in Appendix S1; future updates will be made available via the new online repository (https://github.com/ASAFind/ASAFind‐2). During the updates, ASAFind.py was made fully Python 3 compatible. A web‐based interface for running predictions without locally installed Python is available at https://asafind.jcu.cz/.

For the calculation of custom scoring matrices, we included the Python script S2_score_table_updated.py, which is also available via the above‐mentioned online repository. Scoring matrices can be generated from user‐supplied collections of 25 position sequence windows in FASTA format.

For the calculation of a scoring matrix for PPC proteins, three sets of experimentally localized proteins were assembled: direct experimental results obtained in *T. pseudonana* (Samukawa et al., 2014; Tanaka et al., 2014), or in *Phaeodactylum tricornutum* (the 35 unambiguous BLS phenotype sequences listed in table S3 of Gruber et al., 2015), and heterologous expression experiments of *Guillardia theta* sequences tested in *P. tricornutum* (Gould, Sommer, Hadfi, et al., 2006; Gould, Sommer, Kroth, et al., 2006) (Figure 2). *G. theta* homologs of the *P. tricornutum* set were taken from Curtis et al. (2012). Other best BLAST hits of proteins from the experimental sets were identified in the non‐redundant *G. theta* gene catalog (Curtis et al., 2012) and in the optimized non‐redundant *T. pseudonana* gene catalog (dataset S1 of Gruber et al., 2015, developed from the genome data published by Armbrust et al., 2004). Only the best BLAST (Altschul et al., 1997) hit of each sequence in each genome was selected, leading to 65 potential PPC proteins. Each protein was only allowed to stay in the set if it could be reasonably considered a homolog of the original BLAST query protein, if prior knowledge about metabolism would be in line with the hit being a PPC protein, if the hit had a signal peptide, and if it was not predicted to be a plastid protein (the rationale of selection is provided for each protein in Appendix S2, sequences and prediction results in Appendix S3). This led to a total of 31 proteins for the calculation of a scoring matrix (using the script provided in Appendix S1).

Sequence logos (Schneider & Stephens, 1990) and frequency plots were calculated using the WebLogo online service (http://weblogo.berkeley.edu/, Crooks et al., 2004).

The reference set from Gruber et al. (2015), who compiled the results of 32 publications (Allen et al., 2011, 2012; Apt et al., 2002; Ast et al., 2009; Bruckner et al., 2011; Bullmann et al., 2010; Burmeister, 2009; Domergue et al., 2003; Felsner et al., 2010; Gould, Sommer, Kroth, et al., 2006; Grouneva et al., 2011; Gruber et al., 2007, 2009; Hempel et al., 2009, 2010; Joshi‐Deo et al., 2010; Kilian & Kroth, 2004, 2005; Kitao et al., 2008; Kitao & Matsuda, 2009; Kroth et al., 2005; Lepetit et al., 2007; Liaud et al., 2000; Materna, 2006; Moog et al., 2011; Siaut et al., 2007; Sommer et al., 2007; Sturm et al., 2013; Tachibana et al., 2011; Tanaka et al., 2005; Vugrinec et al., 2011; Weber et al., 2009) to 134 experimentally verified protein localizations, was supplemented with localization data from 28 studies that have been published since then (Balamurugan et al., 2017; Buhmann et al., 2016; Chen et al., 2018; Dell'Aquila et al., 2020; Erdene‐Ochir et al., 2019; Ewe et al., 2018; Gile et al., 2015; Hao et al., 2018; Huang et al., 2016, 2018; Jallet et al., 2020; Lau et al., 2015; Lau et al., 2016; Leyland et al., 2020; Liu et al., 2016; Marter et al., 2020; Mix et al., 2018; Montsant et al., 2005; Moog et al., 2015; Niu et al., 2016; Peschke et al., 2013; Río Bártulos et al., 2018; Schellenberger Costa et al., 2013; Schreiber et al., 2017; Seo et al., 2020; Shao et al., 2019; Stork et al., 2012; Wang et al., 2018), resulting in a total of 284 proteins with experimentally verified intracellular locations (lipid droplet proteins omitted, Appendix S4). Protein sequences were added according to the *P. tricornutum* optimized gene catalog (dataset S2 of Gruber et al., 2015, developed from the genome data published by Bowler et al., 2008). Proteins localized via isolation of lipid droplets were not considered for statistical calculations for two reasons; one is that proteins can be targeted to lipid droplets via the endomembrane system, or via the cytosol (Leyland et al., 2020), and hence cannot be categorically classified as signal peptide containing or not. The other reason is that among the 662 proteins identified by Leyland et al. (2020) (without excluding proteins based on pre‐sequence prediction, “jpy13063‐sup‐0008‐tableS3.xls” from Leyland et al., 2020), 37 proteins were independently localized in other experimental studies. None of these proteins were confirmed as lipid droplet proteins in the respective other study, and the conflicting results point to a high number of other intracellular destinations (Appendix S4). This indicates that the sensitivity of the method applied by Leyland et al. (2020) is set to such a high level that it also identified contaminating proteins from other intracellular locations. Leyland et al. (2020) reported that the majority of the identified lipid droplet proteins do not contain known targeting signals, which our re‐analysis confirmed (precise prediction data for all sequences are in Appendix S6, also see Appendix S4 for protein IDs and for the sequence corrections).

Localization data were only used for statistical analyses if the protein could be unambiguously assigned to one of the result categories (categories in Figure 3, for details on the protein assignments see Appendix S4). Proteins with identical N‐termini (first 60 residues) were combined into just one sequence in the reference set. Appendix S4 contains information on all proteins, including the ones that were not considered for the statistical analyses; Appendix S5 contains only the final reference set (262 sequences).

TargetP 2.0 (Almagro Armenteros, Salvatore, et al., 2019) was used in a portable version, which can be obtained via the TargetP 2.0 web site (https://services.healthtech.dtu.dk/service.php?TargetP). TargetP was used with the “non‐plant” organism group selected, which is generally recommended for organisms with complex plastids, because otherwise, TargetP will also search for chloroplast transit peptides known from primary plastids of higher plants and green algae, a type of targeting pre‐sequence that does not exist in organisms with complex plastids (Gruber & Kroth, 2014, 2017; Moog, 2018).

For comparison, prediction results of SignalP 3.0 NN (Bendtsen et al., 2004), SignalP 4.1 (Nielsen, 2017; Petersen et al., 2011) (with default and “sensitive” settings) and SignalP 5.0 (Almagro Armenteros, Tsirigos, et al., 2019) (with the “Eukarya” organism group selected) were obtained via the SignalP web server (http://www.cbs.dtu.dk/services/SignalP/; which has since then moved to https://services.healthtech.dtu.dk/services/SignalP‐6.0/).

HECTAR (Gschloessl et al., 2008) predictions were obtained from the Galaxy (Goecks et al., 2010) web service provided by the Station Biologique de Roscoff in collaboration with ABiMS (https://webtools.sb‐roscoff.fr/). HECTAR has five possible result categories: “chloroplast”, “signal peptide”, “signal anchor”, “mitochondrion”, and “other localization”. Because our dataset contains no experimentally confirmed type II signal anchor protein, and since all proteins with this prediction result are in compartments that are reached via the secretory pathway, we combined the categories “signal peptide” and “signal anchor” to “secretory pathway”, and counted the predictions as correct if the experimental location was in a compartment of the secretory pathway other than the plastid; see Appendix S5 for classifications (this accounting is in favor of HECTAR).

Prediction statistics were calculated with the help of the Python module scikit‐learn (Pedregosa et al., 2011), which follows the formulas given by Matthews (1975), Baldi et al. (2000), and Gorodkin (2004).

For genome‐wide analyses, the N‐terminally extended gene catalogs for *P. tricornutum* and *T. pseudonana* published in Gruber et al. (2015), and the proteins from the newest *P. tricornutum* genome version (available at https://protists.ensembl.org/Phaeodactylum_tricornutum/Info/Index?db=core) were downloaded, ASAFind 2. predictions were based on TargetP 2.0 (Almagro Armenteros, Salvatore, et al., 2019), and KEGG annotations were obtained via KAAS (https://www.genome.jp/tools/kaas/, Moriya et al. (2007); program: BLAST; method: BBH; GENES data set: hsa, mmu, rno, dre, dme, cel, ath, sce, ago, cal, spo, ecu, pfa, cho, ehi, eco, nme, hpy, bsu, lla, mge, mtu, syn, aae, mja, ape, pti). Prediction data and KEGG annotations are provided in Appendices S6 and S7.

Nucleus encoded proteins of the experimentally determined *Thalassiosira pseudonana* organelle proteomes published by Schober et al. (2019) were extracted from the supplemental data files (“pcz097_supplementary_file_MF.xlsx”, “pcz097_Supplementary_file_PF.xlsx”, and “pcz097_supplementary_file_Tig19.xlsx” of Schober et al., 2019), and the identified proteins were marked in the *T. pseudonana* optimized gene catalog prediction results (Appendix S7). The distribution of the organelle proteins over the different prediction result categories is shown in Appendix S11.

## AUTHOR CONTRIBUTIONS

Conceptualization: AG, GR, and MO. Methodology: AG and GR. Software: MV, CM, GR, and AG. Investigation: AG, MV, CM, MO, and GR. Data curation: AG. Validation: AG. Visualization: MV, AG. Writing – original draft: AG. Writing – review and editing: AG.

## CONFLICT OF INTEREST

The authors declare that they have no conflict of interest.

## Supporting information


**Appendix S1.** Source code of ASAFind 2.0, including scripts for prediction, custom matrix calculation and graphical output generation, and the scoring matrices mentioned in the manuscript. For installation and usage instructions, see the contained readme file, our online repository: https://github.com/ASAFind/ASAFind‐2, or web‐service: https://asafind.jcu.cz/.

**Appendix S2.** Composition of scoring matrix for PPC protein prediction, details on the selection of proteins. For experimental evidence in *P. tricornutum* proteins, compare to S6; for other literature, digital object identifiers (DOIs) are given.

**Appendix S3.** Sequences and ASAFind 2 prediction results of PPC scoring matrix.

**Appendix S4.** Reference set of experimentally localized *P. tricornutum* proteins.

**Appendix S5.** Prediction results for reference set and classification of the results for the calculation of statistical parameters.

**Appendix S6.** Intracellular location predictions and KEGG annotation for the *Phaeodactylum tricornutum* extended gene catalog published in Gruber et al. (2015). ASAFind 2.0 predictions are based on TargetP 2.0 (Almagro Armenteros, Salvatore, et al., 2019); KEGG annotations were obtained via KAAS (Moriya et al., 2007).

**Appendix S7.** Intracellular location predictions and KEGG annotation for the *Thalassiosira pseudonana* extended gene catalog published in Gruber et al. (2015). ASAFind 2 predictions are based on TargetP 2.0 (Almagro Armenteros, Salvatore, et al., 2019); KEGG annotations were generated via KAAS (Moriya et al., 2007); proteome data was obtained from Schober et al. (2019).

**Appendix S8.** Intracellular location predictions for the *Phaeodactylum tricornutum* gene catalog version 3 (downloaded from https://protists.ensembl.org/Phaeodactylum_tricornutum/Info/Index?db=core). ASAFind 2 predictions are based on TargetP 2.0 (Almagro Armenteros, Salvatore, et al., 2019).

**Appendix S9.** Composition of scoring matrix assembled from cryptophyte plastid‐targeted proteins.

**Appendix S10.** Sequence logos and frequency plots for cryptophyte custom scoring matrix (Appendix .

**Appendix S11.** (a) Overlaps of *Phaeodactylum tricornutum* PPC protein predictions (Appendix S6) with experimental data (Appendix S4). Direct sequence identity (“Proteins”) and KEGG category representations (“*K* numbers”) are shown. (b) Annotated KEGG categories of predicted PPC proteins for *Phaeodactylum tricornutum* (“Phatr”) and *Thalassiosira pseudonana* (“Thaps”), raw data in Appendices S6 and S7. (c) Prediction results for experimentally determined organelle proteins; proteome data from Schober et al. (2019), prediction data from Appendix S7.

## Data Availability

All raw data and the source code of the software are included in the Supporting Information (Appendices [Link], [Link], [Link], [Link], [Link], [Link], [Link], [Link], [Link], [Link], [Link]). The software source code is additionally publicly accessible at https://github.com/ASAFind/ASAFind‐2 and can be run through our web service https://asafind.jcu.cz/.
