# Supplementary figures and images for: ASAFind 2.0: multi‐class protein targeting prediction for diatoms and algae with complex plastids
Source: Plant J. 2025 Jun 4;122(5):e70138. doi: 10.1111/tpj.70138 (PMC12136025; doi:10.1111/tpj.70138)

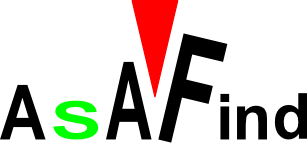

Supplement: Supplementary file 1 — Appendix S1. Source code of ASAFind 2.0, including scripts for prediction, custom matrix calculation and graphical output generation, and the scoring matrices mentioned in the manuscript. For installation and usage instructions, see the contained readme file, our online repository: https://github.com/ASAFind/ASAFind‐2, or web‐service: https://asafind.jcu.cz/. [file TPJ-122-0-s009.zip › ASAFind-2-main/ASAFind_logo_small.png]

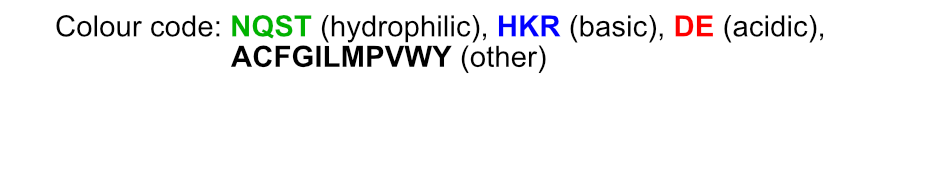

Supplement: Supplementary file 1 — Appendix S1. Source code of ASAFind 2.0, including scripts for prediction, custom matrix calculation and graphical output generation, and the scoring matrices mentioned in the manuscript. For installation and usage instructions, see the contained readme file, our online repository: https://github.com/ASAFind/ASAFind‐2, or web‐service: https://asafind.jcu.cz/. [file TPJ-122-0-s009.zip › ASAFind-2-main/colour_code.png]

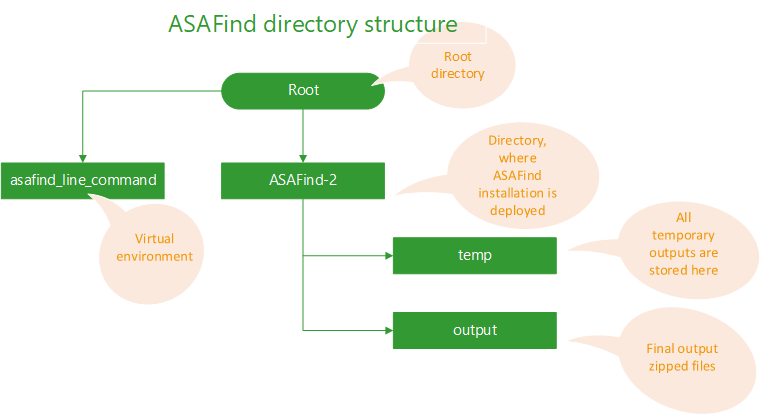

Supplement: Supplementary file 1 — Appendix S1. Source code of ASAFind 2.0, including scripts for prediction, custom matrix calculation and graphical output generation, and the scoring matrices mentioned in the manuscript. For installation and usage instructions, see the contained readme file, our online repository: https://github.com/ASAFind/ASAFind‐2, or web‐service: https://asafind.jcu.cz/. [file TPJ-122-0-s009.zip › ASAFind-2-main/directories.png]

Scoring matrix from 102 Cryptophyte proteins

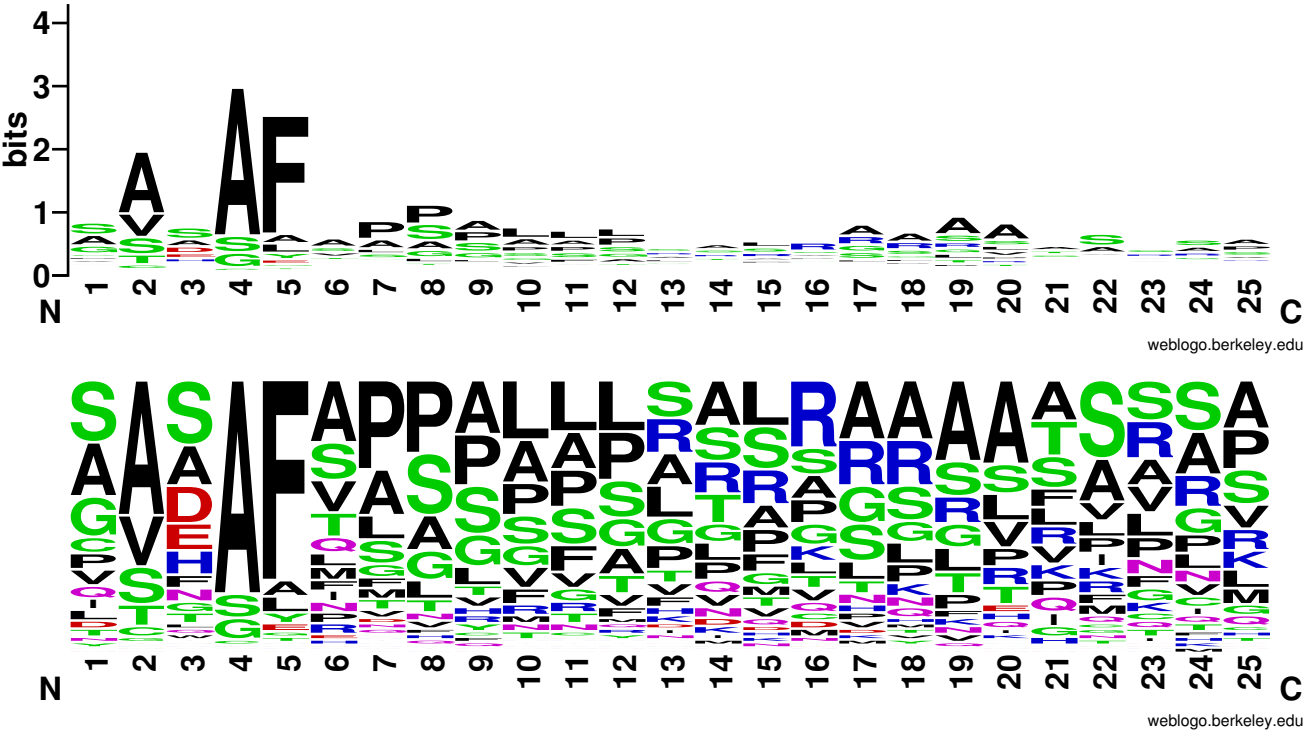

Supplement: Supplementary file 10 — Appendix S10. Sequence logos and frequency plots for cryptophyte custom scoring matrix (Appendix . [file TPJ-122-0-s006.pdf]
