# Supplementary material for: ASAFind 2.0: multi‐class protein targeting prediction for diatoms and algae with complex plastids
Source: Plant J. 2025 Jun 4;122(5):e70138. doi: 10.1111/tpj.70138 (PMC12136025; doi:10.1111/tpj.70138)
Supplement: Supplementary file 2 — Appendix S2. Composition of scoring matrix for PPC protein prediction, details on the selection of proteins. For experimental evidence in P. tricornutum proteins, compare to S6; for other literature, digital object identifiers (DOIs) are given. [file TPJ-122-0-s008.pdf]

Organism	ID	Name<br>(or name of BLAST query)	25 residue sequence window	Method, References, Comments
Thaps	Thaps3a_26706	salpha7-1		Best BLAST hit to Phatr2a_17388, not used due to lack of predicted signal peptide
Thaps	Thaps3a_22298	sCdc48-2	STIEARSSSTSYLPSNGGSLFGIQK	Best BLAST hit to Phatr2a_19162
Thaps	Thaps3a_22996	sbeta7		Best BLAST hit to Phatr2a_22110, not used due to positive plastid protein prediction by ASAFind
Thaps	Thaps3a_23853	sTLP-1	SPSSAETIRGRADSAVVVGYNEEVN	Best BLAST hit to Phatr2a_23414
Thaps	Thaps3a_9466	Der1-1	GAVTSKHHPSPFQPRRTFTKPLNSA	Best BLAST hit to Phatr2a_31697
Thaps	Thaps3a_2280	sORF139	TVTYASMPSFMTKRSHYTPLLFFKV	Best BLAST hit to Phatr2a_31704
Thaps	Thaps3a_22391	CA-I/\-s alpha CA-1	QSSNSRSWLDCINTSKLENDGMPRV	Best BLAST hit to Phatr2a_35370 and Phatr2a_44526
Thaps	Thaps3a_22447	sDer1-2		Best BLAST hit to Phatr2a_35965, not used due to positive plastid protein prediction by ASAFind
Thaps	Thaps3a_4435	sSMC	ADSSAAIDLGRAFGKATKDGATKGG	Best BLAST hit to Phatr2a_42675
Thaps	Thaps3a_1526	sSec14		Best BLAST hit to Phatr2a_44766, not used due to positive plastid protein prediction by ASAFind
Thaps	Thaps3a_8632	sDTC	APLQLLRLGGAASSSSSSAGGININ	Best BLAST hit to Phatr2a_44959
Thaps	Thaps3a_33343	PGDH		Best BLAST hit to Phatr2a_45333, not used due to lack of predicted signal peptide
Thaps	Thaps3a_22550	sbeta2	QLACAQQSAAGQDTLIAVVGRDFVM	Best BLAST hit to Phatr2a_45347
Thaps	Thaps3a_23216	sORF532a	PSTHAATTLIDSTGLLTKAIPSISQ	Best BLAST hit to Phatr2a_45935
Thaps	Thaps3a_8351	sPUB		Best BLAST hit to Phatr2a_47444, not used due to lack of predicted signal peptide
Thaps	Thaps3a_21399	sP4H		Best BLAST hit to Phatr2a_47766, not used due to positive plastid protein prediction by ASAFind
Thaps	Thaps3a_25775	ptE3P	SNVNATITLSTTGRQYHSRPASFGM	Best BLAST hit to Phatr2a_48034
Thaps	Thaps3a_19991	TrxH	SPALASSGSSFSRRTFGVVSVPPSS	Best BLAST hit to Phatr2a_48539
Thaps	Thaps3a_2811	sDPC	EICSAEAADGRRRQQQQRNNSRRPN	Best BLAST hit to Phatr2a_48633
Thaps	Thaps3a_25820	sORF534		Best BLAST hit to Phatr2a_48879, not used due to lack of predicted signal peptide
Thaps	Thaps3a_256895	sbeta6		Best BLAST hit to Phatr2a_49432, not used due to lack of predicted signal peptide

Organism	ID	Name (or name of BLAST query)	25 residue sequence window	Method, References, Comments
Thaps	Thaps3a_22697	sCdc48	TSVSATLPPTGTFGVIRNNVRSSQA	Best BLAST hit to Phatr2a_50978
Thaps	Thaps3a_267126	Ubi		Best BLAST hit to Phatr2a_54323, not used due to lack of predicted signal peptide
Thaps	Thaps3a_7676	TRD1		Best BLAST hit to Phatr2a_54863, not used due to lack of predicted signal peptide
Thaps	Thaps3a_258538	Hsp70_2		Best BLAST hit to Phatr2a_55890, not used due to lack of predicted signal peptide
Thaps	Thaps3a_265333	GLRX2		Best BLAST hit to Phatr2a_56497, not used due to positive plastid protein prediction by ASAFind
Thaps	Thaps3a_22129	NTRC		Best BLAST hit to Phatr2a_56519, not used due to lack of predicted signal peptide
Thaps	Thaps3a_20944	unknown protein		Best BLAST hit to Phatr2a_56648, not used due to lack of predicted signal peptide
Thaps	Thaps3a_270405	sDrp (Drp5b)	NELTAERGNNSGLSKSNNRASMSPT	Best BLAST hit to Phatr2a_56658
Thaps	Thaps3a_21460	ptDUP		Best BLAST hit to Phatr2a_56710, not used due to lack of predicted signal peptide
Thaps	Thaps3a_6655	salpha7-2		Best BLAST hit to Phatr2a_56730, not used due to positive plastid protein prediction by ASAFind
Thaps	Thaps3a_24247	sORF261	SSVDASHVVSGTTARLPMSLSTVIN	Best BLAST hit to Phatr2a_56731
Thaps	Thaps3a_40393	sPRP		Best BLAST hit to Phatr2a_56754, not used due to lack of predicted signal peptide
Thaps	Thaps3a_21581	sPEL		Best BLAST hit to Phatr2a_56757, not used due to lack of predicted signal peptide
Guith	gt_165822	sDer1-2		Best BLAST hit to sDer1-2 (Phatr2a_35965, in doi:10.1038/nature11681 erroneously a wrong protein ID and sequence has been used), not used due to lack of predicted signal peptide
Guith	gt_164379	sPEL		Best BLAST hit to Phatr2a_56757, from doi:10.1038/nature11681, not used due to positive plastid protein prediction by ASAFind
Guith	gt_155024	Ubi	VLSSADSMAPLSSSPSMSPSPRQLL	Best BLAST hit to Phatr2a_54323, from doi:10.1038/nature11681
Guith	gt_72696	sDTC	INTNASSEKTDSLSAGAAAHERSLG	Best BLAST hit to Phatr2a_44959, from doi:10.1038/nature11681
Guith	gt_157105	sDTC	MQAAAEKDLYKILNVPRNADEKAIK	Best BLAST hit to Phatr2a_44959, from doi:10.1038/nature11681

Organism	ID	Name (or name of BLAST query)	25 residue sequence window	Method, References, Comments
Guith	gt_166408	sTLP-1	LCTDAKPAVIKAIPTTATEKSWFHS	Best BLAST hit to Phatr2a_23414, from doi:10.1038/nature11681
Guith	gt_161858	Cdc48	SLVDADISVNGNMLLGMKQQGLSQS	Best BLAST hit to Phatr2a_50978, from doi:10.1038/nature11681
Guith	gt_72625	NTRC	QTAHSRPLVRNVKDHNEYKKLLKHH	Best BLAST hit to Phatr2a_56519, from doi:10.1038/nature11681
Guith	gt_92918	GLRX2	AVVQGKSAEDHMMDAIKQHKVQIFS	Best BLAST hit to Phatr2a_56497, from doi:10.1038/nature11681
Guith	gt_151103_85246	sTLP-1	SFIHATPVGVSQLQQNNVHPNALGG	Best BLAST hit to Phatr2a_23414, from doi:10.1038/nature11681
Guith	gt_154814	sbeta6		Best BLAST hit to Phatr2a_49432, from doi:10.1038/nature11681, not used due to lack of predicted signal peptide
Guith	gt_164385	ugg-t	TPSNASTGVSSFSSSHRIPSLTRSA	GFP fusion and expression in Phaeodactylum tricornutum doi:10.1007/s00239-005-0099-y
Guith	gt_76641	hemE	ALAMAAEVQGYAMTRAGGANFVSGL	Listed as example ppc protein in doi:10.1007/s00239-005-0099-y
Guith	gt_155857	iddi		Listed as example ppc protein in doi:10.1007/s00239-005-0099-y, not used due to positive plastid protein prediction by ASAFind
Guith	gt_84077	EF-1 alpha	ATTMGAAGRTFSAMGHRMNMIPSGT	GFP fusion and expression in Phaeodactylum tricornutum doi:10.1093/molbev/msl113
Guith	gt_96401	14.03.03	TVASSSSLSSPFPTKLLQSRPSRST	GFP fusion and expression in Phaeodactylum tricornutum doi:10.1093/molbev/msl113
Guith	gt_85468	RRM1	EEVNSDRACRSISSPLRLRGGFRGD	GFP fusion and expression in Phaeodactylum tricornutum doi:10.1093/molbev/msl113
Guith	gt_97923	CyP	GICSANYASRYAPMAGPRKFLGDGE	GFP fusion and expression in Phaeodactylum tricornutum doi:10.1093/molbev/msl113
Guith	gt_97471	Ubc8	PLVLSSSPSPTDLVRLSSSRLRGAL	GFP fusion and expression in Phaeodactylum tricornutum doi:10.1093/molbev/msl113
Thaps	Thaps3a_38775	ugg-t		best BLAST hit to gt_164385, not used because carbohydrate storage is different between diatoms and cryptophytes
Thaps	Thaps3a_719	hemE		best BLAST hit to gt_76641, not used due to lack of predicted signal peptide
Thaps	Thaps3a_21777	iddi		best BLAST hit to gt_155857, not used due to lack of predicted signal peptide
Thaps	Thaps3u_1861	EF-1 alpha		best BLAST hit to gt_84077, not used due to lack of predicted signal peptide
Thaps	Thaps3a_26146	14.03.03		best BLAST hit to gt_96401, not used due to lack of predicted signal peptide

Organism	ID	Name (or name of BLAST query)	25 residue sequence window	Method, References, Comments
Thaps	Thaps3a_21496	RRM1		best BLAST hit to gt_85468, not used due to lack of predicted signal peptide
Thaps	Thaps3a_264484	CyP		best BLAST hit to gt_97923, not used due to lack of predicted signal peptide
Thaps	Thaps3a_23095	Ubc8		best BLAST hit to gt_97471, not used due to lack of predicted signal peptide
Thaps	Thaps3a_255451	Tp delta CA3		GFP fusion and expression in Thalassiosira pseudonana doi:10.1007/s11120-014-9967-x, not used due to lack of predicted signal peptide
Thaps	Thaps3a_5027	PEPC1	QSATARSSSPATPLANLASASKRAF	GFP fusion and expression in Thalassiosira pseudonana doi:10.1007/s11120-014-9968-9 (protein ID in publication: 270453)
Guith	gt_143978	Tp delta CA3		Best BLAST hit to Thaps3a_255451, not used due to low bit-score and lack of predicted signal peptide
Guith	gt_121941	PEPC1		Best BLAST hit to Thaps3a_5027, not used due to lack of predicted signal peptide
