# Supplementary material for: ASAFind 2.0: multi‐class protein targeting prediction for diatoms and algae with complex plastids
Source: Plant J. 2025 Jun 4;122(5):e70138. doi: 10.1111/tpj.70138 (PMC12136025; doi:10.1111/tpj.70138)
Supplement: Supplementary file 4 — Appendix S4. Reference set of experimentally localized P. tricornutum proteins. [file TPJ-122-0-s001.pdf]

Table S6: Reference set. Proteins with experimentally determined intracellular location in *Phaeodactylum tricornutum*. Numerical parts of protein IDs refer to the U.S. Department of Energy Joint Genome Institute *Phaeodactylum tricornutum* v2.0 database (http://genome.jgi-psf.org/Phatr2/Phatr2.home.html) [7], complete protein IDs are from Dataset S2 of Gruber et al. [21]. BLS, 'blob'-like structure [32]; FCP, fucoxanthin chlorophyll a/c-binding protein; ies, inter envelope space (compartment between the innermost and second innermost plastid membranes); LD, lipid droplets; Pl, plastid; PS, Photosystem.

Protein ID	Name	Location	Method, References, Comments	Contained in reference set of [21]?	Used for statistics?
20657	AtpC	Pl	GFP fusion [3, 31, 32, 16, 37, 41] + self-assembling GFP with OEE1 [61] + mRuby3 fusion [42]	yes	yes
49533	Ntt1	Pl	GFP fusion [4]	yes	yes
53935	Rpe	Pl	GFP fusion [11]	yes	yes
48423	PtFAD6	Pl	GFP fusion [13]	yes	yes
Phatr2a_49339	PYC2 (pyruvate carboxylase 2)	Pl	GFP fusion [15]	no	yes
Phatr2a_49027	RP1-PPDK (PPDK regulator protein 1)	Pl	GFP fusion [15]	no	yes
20779	FSA	Pl	GFP fusion [22]	yes	yes
55112	Hlip2	Pl	GFP fusion [22]	yes	yes
54279	FBPC4	Pl	GFP fusion [23, 22], in the first study this sequence gave an ambigous result (plastid plus additional compartment?), never any BLS	yes	yes
42886	FBPC1	Pl	GFP fusion [23]	yes	yes
42456	FBPC2	Pl	GFP fusion [23]	yes	yes
31451	FBPC3	Pl	GFP fusion [23]	yes	yes
38631	PGL	Pl	GFP fusion [23]	yes	yes
Phatr2a_33198	ptTES1	Pl	GFP fusion [24]	no	yes
Phatr2u_825	FbaC1	Pl	GFP fusion [31, 32, 35, 22] + YFP fusion [2], unmapped sequence	yes	yes
24610	Tpt1/TPT4b	Pl	GFP fusion [31, 32] + GFP fusion and self-assembling GFP analyses [46], model name in publication [46] is estExt_Genewise1.C_chr_10395	yes	yes
45443	PtCa2	Pl	GFP fusion [33]	yes	yes
54013	RecA	Pl	GFP fusion [43]	yes	yes
46781	PPG1	Pl	GFP fusion [47], protein ID in reference by Moog et al.[47]: 36817	yes	yes
Phatr2a_29157	PGK	Pl	GFP fusion [50]	no	yes
Phatr2a_50738	TPI	Pl	GFP fusion [50], protein ID in publication is 18228 (erroneously)	no	yes

Protein ID	Name	Location	Method, References, Comments	Contained in reference set of [21]?	Used for statistics?
56798	PGM	Pl	GFP fusion [50], protein ID in publication is 42857, the gene model Protein ID 56857 is not part of the optimized gene catalog published in [21]	no	yes
56857	TPI	Pl	GFP Fusion [50], Protein ID in publication is 50738, the gene model Protein ID 56857 is not part of the optimized gene catalog published in [21]	no	yes
Phatr2a_43099	LPAT1/AGPAT1	Pl	GFP fusion [62] + immuno electron microscopy of transformed cell lines with Myc antibody [5]	no	yes
Phatr2a_42446	GPAT1	Pl	GFP fusion [62] + immuno electron microscopy of transformed cell lines with Myc antibody [48]	no	yes
50907	FtrB	Pl	GFP fusion [63]	yes	yes
46280	Trx-f	Pl	GFP fusion [63]	yes	yes
51357	Trx-m	Pl	GFP fusion [63]	yes	yes
33356	Trx-y	Pl	GFP fusion [63]	yes	yes
Phatr2a_48863	Glx	Pl	GFP fusion + immuno electron microscopy with GFP antibody [49]	no	yes
Phatr2a_47730	MutS	Pl	GFP fusion + immuno electron microscopy with GFP antibody [49]	no	yes
Phatr2a_43097	Syn	Pl	GFP fusion + immuno electron microscopy with GFP antibody [49]	no	yes
54017	TPT4a	Pl	GFP fusion + self-assembling GFP analyses [46], model name in publication is estExt_Phatr1_ua_kg.C_chr_10107, ID in publication is 54017	no	yes
22122	GapC1	Pl	Immuno electron microscopy with GapC1 antibody [40]	yes	yes
22993	FbaC2	Pl	YFP fusion [2], pre-sequence is processed according to western blot analysis [49]	yes	yes
Phatr2a_45177	PtTPT	Pl	YFP fusion [53], ID in publication is PHATRDRAFT_3046 (GenBank XP_002179421)	no	yes
51289	FbaC5	Pl	YFP fusion and immuno electron microscopy with GFP antibody [2]	yes	yes
21505	MGD1	Pl (ies)	GFP fusion and self-assembling GFP with PtSP1 [10], supposedly enters the ies via the stroma (no experimental data on this process available), N-terminal extension possible, compare to protein ID 56712	yes	yes
17972	PtSP1	Pl (ies)	Self assembling GFP with MGD1 [10], supposedly enters the ies via the stroma (no experimental data on this process available)	yes	yes
Phatr2a_20143	ptACSL1	Pl (LD)	GFP fusion [12] + Mass spectrometric peptide mapping of proteins from isolated lipid droplets [39], sequence identical to UniProt B7FYK0; counted as Pl, the identification in LD fraction is considered a contamination	no	yes

Protein ID	Name	Location	Method, References, Comments	Contained in reference set of [21]?	Used for statistics?
20331	OEE1	Pl (LD)	GFP fusion [31, 32, 22] + self-assembling GFP with AtpC [61] + Mass spectrometric peptide mapping of proteins from isolated lipid droplets [39], UniProt ID in publication [39] is B7FZ96; counted as Pl, the identification in LD fraction is considered a contamination. (Protein ID 20331 is not part of the optimized gene catalog published in [21])	yes	yes
42406	PtCA1	Pl (LD)	GFP fusion [59, 60, 33, 34] + immuno electron microscopy with GFP antibody [59] + CFP fusion [2] + Mass spectrometric peptide mapping of proteins from isolated lipid droplets [39], sequence identical to UniProt B7FNU0, here counted as Pl, the occurence in the lipid droplet sample is counted as contamination	yes	yes
51092	GSII	Pl (LD)	YFP fusion [55] + Mass spectrometric peptide mapping of proteins from isolated lipid droplets [39], sequence identical to UniProt B7G5A1; counted as Pl, the identification in LD fraction is considered a contamination	yes	yes
56741	ptOmp85	Pl (third outermost envelope membrane)	GFP fusions of different lengths and self-assembling GFP with Hsp70 [10], supposedly enters the third outermost envelope membrane via the stroma (no experimental data on this process available) + GFP fusion [41], protein ID in publication [41] is 46881	yes	yes
50705	Lhcf3, FcpC	Pl (thylakoid membrane, FCP trimer)	sucrose density gradient centrifugation followed by SDS-PAGE and mass spectrometry [38] + GFP fusion [16] + Two-dimensional BN/SDS-PAGE of thylakoid membranes followed by mass spectrometry [20], identical aa sequence as Lhcf4/FcpD (in original publication [6], fcpC and fcpD differ slightly from each other, and from the Phatr2 lhcf3/4)	yes	yes
25168	Lhcf4, FcpD	Pl (thylakoid membrane, FCP trimer)	sucrose density gradient centrifugation followed by SDS-PAGE and mass spectrometry [38] + GFP fusion [16] + Two-dimensional BN/SDS-PAGE of thylakoid membranes followed by mass spectrometry [20], not used for statistics; full sequence is identical to Lhcf3/FcpC (in original publication [6], fcpC and fcpD differ slightly from each other, and from the Phatr2 lhcf3/4)	yes	no
30648	Lhcf5/FcpE	Pl (thylakoid membrane, FCP trimer)	Sucrose density gradient centrifugation followed by SDS-PAGE and mass spectrometry [38] + mass spectrometry and westernblot after thylakoid membrane preparation [30] + Two-dimensional BN/SDS-PAGE of thylakoid membranes followed by mass spectrometry [20]	yes	yes
22006	Lhcf10	Pl (thylakoid membrane, FCP trimer)	Two-dimensional BN/SDS-PAGE of thylakoid membranes followed by mass spectrometry [20]	yes	yes
49912	Lhcf12	Pl (thylakoid membrane, FCP trimer)	Two-dimensional BN/SDS-PAGE of thylakoid membranes followed by mass spectrometry [20]	yes	yes
25172	Lhcf2	Pl (thylakoid membrane, FCP trimer)	Two-dimensional BN/SDS-PAGE of thylakoid membranes followed by mass spectrometry [20]	yes	yes
30031	Lhcf9	Pl (thylakoid membrane, FCP trimer)	Two-dimensional BN/SDS-PAGE of thylakoid membranes followed by mass spectrometry [20]	yes	yes

Protein ID	Name	Location	Method, References, Comments	Contained in reference set of [21]?	Used for statistics?
18049	Lhcf1/FcpA	Pl (thylakoid membrane, FCP trimer)	Two-dimensional BN/SDS-PAGE of thylakoid membranes followed by mass spectrometry [20], mass spectrometry and westernblot after thylakoid membrane preparation [30], GFP fusion [14]	yes	yes
51230	Lhcf11	Pl (thylakoid membrane, FCP trimer)	Two-dimensional BN/SDS-PAGE of thylakoid membranes followed by mass spectrometry [20], not used for statistics; first 67 residues are identical to Lhcf5/FcpE	yes	no
56689	Lhcx2	Pl (thylakoid membrane, free protein)	Two-dimensional BN/SDS-PAGE of thylakoid membranes followed by mass spectrometry [20]	yes	yes
42543	predicted, PGRL	Pl (thylakoid membrane, free protein)	Two-dimensional BN/SDS-PAGE of thylakoid membranes followed by mass spectrometry [20]	yes	yes
22395	Lhcf8	Pl (thylakoid membrane, PS I + FCP trimer)	Two-dimensional BN/SDS-PAGE of thylakoid membranes followed by mass spectrometry [20]	yes	yes
27278	Lhcx1	Pl (thylakoid membrane, PS I + free protein)	Two-dimensional BN/SDS-PAGE of thylakoid membranes followed by mass spectrometry [20], N-terminal extension possible, compare to protein ID 56679	yes	yes
34536	Lhcf16	Pl (thylakoid membrane, PS I)	Two-dimensional BN/SDS-PAGE of thylakoid membranes followed by mass spectrometry [20]	yes	yes
56310	Lhcf17	Pl (thylakoid membrane, PS I)	Two-dimensional BN/SDS-PAGE of thylakoid membranes followed by mass spectrometry [20]	yes	yes
44601	Lhcr1	Pl (thylakoid membrane, PS I)	Two-dimensional BN/SDS-PAGE of thylakoid membranes followed by mass spectrometry [20]	yes	yes
23257	Lhcr11	Pl (thylakoid membrane, PS I)	Two-dimensional BN/SDS-PAGE of thylakoid membranes followed by mass spectrometry [20]	yes	yes
54027	Lhcr12	Pl (thylakoid membrane, PS I)	Two-dimensional BN/SDS-PAGE of thylakoid membranes followed by mass spectrometry [20]	yes	yes
47813	Lhcr14	Pl (thylakoid membrane, PS I)	Two-dimensional BN/SDS-PAGE of thylakoid membranes followed by mass spectrometry [20]	yes	yes
22956	Lhcr2	Pl (thylakoid membrane, PS I)	Two-dimensional BN/SDS-PAGE of thylakoid membranes followed by mass spectrometry [20]	yes	yes
50725	Lhcr3	Pl (thylakoid membrane, PS I)	Two-dimensional BN/SDS-PAGE of thylakoid membranes followed by mass spectrometry [20]	yes	yes
56747	unnamed	Pl (thylakoid membrane, PS I)	Two-dimensional BN/SDS-PAGE of thylakoid membranes followed by mass spectrometry [20]	yes	yes
17326	Lhl1/RedCAP	Pl (thylakoid membrane, PS I)	Two-dimensional BN/SDS-PAGE of thylakoid membranes followed by mass spectrometry [20] + GFP fusion [58]	yes	yes

Protein ID	Name	Location	Method, References, Comments	Contained in reference set of [21]?	Used for statistics?
17766	Lhcr4	Pl (thylakoid membrane, PS I)	Two-dimensional BN/SDS-PAGE of thylakoid membranes followed by mass spectrometry [20], N-terminal extension possible, compare to protein ID 56749	yes	yes
17531	unnamed	Pl (thylakoid membrane, PS I)	Two-dimensional BN/SDS-PAGE of thylakoid membranes followed by mass spectrometry [20], N-terminal extension possible, compare to protein ID 56750	yes	yes
48798	unnamed	Pl (thylakoid membrane, PS I) (LD)	Two-dimensional BN/SDS-PAGE of thylakoid membranes followed by mass spectrometry [20] + Mass spectrometric peptide mapping of proteins from isolated lipid droplets [39], sequence identical to UniProt B7G8E5; counted as Pl, the identification in LD fraction is considered a contamination	yes	yes
56334	Lhcr13	Pl (thylakoid membrane, PS I) (LD)	Two-dimensional BN/SDS-PAGE of thylakoid membranes followed by mass spectrometry [20] + Mass spectrometric peptide mapping of proteins from isolated lipid droplets [39], UniProt ID in publication [39] is B7G502; counted as Pl, the identification in LD fraction is considered a contamination	yes	yes
46336	Predicted, low CO2-inducible	Pl (thylakoid membrane, PS II monomer)	Two-dimensional BN/SDS-PAGE of thylakoid membranes followed by mass spectrometry [20]	yes	yes
Phatr2a_36942	ptSRT5	Pl + nucleus	GFP fusion [12]; not used for statistics due to dual targeting	no	no
Phatr2a_46657	PetC	Pl thylakoid membrane	GFP fusion [41]	no	yes
56497	GLRX2	BLS	GFP fusion [11]	yes	yes
56648	unknown protein	BLS	GFP fusion [11]	yes	yes
56710	ptDUP	BLS	GFP fusion [26]	yes	yes
48034	ptE3P	BLS	GFP fusion [26], split GFP assay and co-immunoprecipitation [36]	yes	yes
Phatr2a_19408	MIP2	BLS	GFP fusion [41], ID in publication is 19409	no	yes
35370	CA-I/sαCA-1	BLS	GFP fusion [47, 59]	yes	yes
17388	salpha7-1	BLS	GFP fusion [47]	yes	yes
49432	sbeta6	BLS	GFP fusion [47]	yes	yes
22110	sbeta7	BLS	GFP fusion [47]	yes	yes
48633	sDPC	BLS	GFP fusion [47]	yes	yes
31704	sORF139	BLS	GFP fusion [47]	yes	yes
48879	sORF534	BLS	GFP fusion [47]	yes	yes
47766	sP4H	BLS	GFP fusion [47]	yes	yes
44766	sSec14	BLS	GFP fusion [47]	yes	yes
42675	sSMC	BLS	GFP fusion [47]	yes	yes

Protein ID	Name	Location	Method, References, Comments	Contained in reference set of [21]?	Used for statistics?
47444	sPUB	BLS	GFP fusion [47], GFP fusion and western blot [49], split GFP assay [36], protein ID in publications: 37661	yes	yes
44959	sDTC	BLS	GFP fusion [47], protein ID in reference by Moog et al.[47]: 34512	yes	yes
45347	sbeta2	BLS	GFP fusion [47], protein ID in reference by Moog et al.[47]: 35028	yes	yes
56658	sDrp (Drp5b)	BLS	GFP fusion [47], protein ID in reference by Moog et al.[47]: 37379, construct tested in [47] is two N-terminal amino acids shorter than this gene model, this is without consequence for the position of the SignalP predicted cleavage site	yes	yes
56757	sPEL	BLS	GFP fusion [47], protein ID in reference by Moog et al.[47]: 37424	yes	yes
56754	sPRP	BLS	GFP fusion [47], protein ID in reference by Moog et al.[47]: 41316	yes	yes
56730	salpha7-2	BLS	GFP fusion [47], protein ID in reference by Moog et al.[47]: 43079	yes	yes
56731	sORF261	BLS	GFP fusion [47], protein ID in reference by Moog et al.[47]: 47811	yes	yes
19162	sCdc48-2	BLS	GFP fusion [47], split GFP assay [36]	yes	yes
Phatr2a_49576	sGt8	BLS	GFP fusion [49], protein ID in publication is 40314	no	yes
Phatr2a_23111	sUfd1	BLS	GFP fusion [49], protein ID in publication is 49319	no	yes
56802	PGM	BLS	GFP fusion [50], protein ID in publication is 51298 (alternative construct length of 56801, with comment "data not shown", probably not a naturally occuring N-terminus, therefore not used for statistics here), the gene model Protein ID 56802 is not part of the optimized gene catalog published in [21]	no	no
35965	sDer1-2	BLS	GFP fusion [56, 25, 41], split GFP assay and co-immunoprecipitation [36] (in [21] erroneously a wrong protein ID and sequence has been used)	yes	yes
31697	sDer1-1	BLS	GFP fusion [56, 25], split GFP assay and co-immunoprecipitation [36]	yes	yes
54323	Ubi	BLS	GFP fusion [56]	yes	yes
50978	sCdc48	BLS	GFP fusion [56], split GFP assay [36]	yes	yes
Phatr2a_45475	Ptsalpha3-1	BLS	GFP fusion [57]	no	yes
Phatr2a_50113	sPng1	BLS	GFP fusion [57]	no	yes
Phatr2a_47962	sUbq	BLS	GFP fusion [57]	no	yes
Phatr2a_45135	sNpl4	BLS	GFP fusion [57], protein ID in publication is 34750	no	yes
Phatr2a_47897	Ptsbeta1	BLS	GFP fusion [57], protein ID in publication is 38228	no	yes
Phatr2a_44122	sUBX	BLS	GFP fusion [57], split GFP assay and co-immunoprecipitation [36]	no	yes
44526	CA-II	BLS	GFP fusion [59], N-terminal extension with predicted mitochondrial transit peptide possible, compare to protein ID 56729	yes	yes

Protein ID	Name	Location	Method, References, Comments	Contained in reference set of [21]?	Used for statistics?
56519	NTRC	BLS	GFP fusion [63], N-terminal extension possible, compare to protein ID 47568	yes	yes
Phatr2a_53360	triose phosphate translocator 2	BLS (integral membrane protein)	GFP fusion and cell fractionation with carbonate extraction of membranes [37], protein ID in Publication is 27949, GFP fusion [46]	no	yes
56855	ptsRhom3	BLS (integral membrane protein)	GFP fusion, cell fractionation with carbonate extraction of membranes, self-assembling GFP with HSP70_2, split GFP assay and co-immunoprecipitation [36], protein ID in Publication is 47107. (The new gene model Protein ID 56855 is not part of the optimized gene catalog published in [21])	no	yes
55890	Hsp70_2	BLS (LD)	GFP fusion [19, 25, 37, 41] + mRuby3 fusion [42] + Mass spectrometric peptide mapping of proteins from isolated lipid droplets [39], sequence identical to UniProt B7G3Y2; counted as BLS, the identification in LD fraction is considered a contamination	yes	yes
45333	PGDH	BLS (LD)	GFP fusion [23] + Mass spectrometric peptide mapping of proteins from isolated lipid droplets [39], sequence identical to UniProt B7FXB5, counted as BLS, the occurence in the lipid droplet sample is counted as contamination	yes	yes
45935	sORF532a	BLS (LD)	GFP fusion [47] + mass spectrometric peptide mapping of proteins from isolated lipid droplets [39], counted as BLS, the occurence in the lipid droplet sample is counted as contamination, sequence identical to UniProt B7FZ76	yes	yes
22529	TrxH	BLS (LD)	GFP fusion [63] + Mass spectrometric peptide mapping of proteins from isolated lipid droplets [39], sequence identical to UniProt B7G7L6; counted as BLS, the identification in LD fraction is considered a contamination	yes	yes
54863	TRD1/epsilon frustulin	BLS/Extracellular	GFP fusion [11] + Extraction of extracellular proteins and nanoLC-MS/MS analysis [9], ID in publication [9] is 38514, not used for statistics due to conflicting results between studies	yes	no
23414	sTLP-1/S1A protease	BLS/Extracellular	GFP fusion [47] + Extraction of extracellular proteins and nanoLC-MS/MS analysis [9], not used for statistics due to conflicting experimental data	yes	no
56768	PEPC1 (phosphoenolpyruvate carboxylase 1)	BLS/Pl	GFP fusion [15]; not used for statistics due to unclear location. (The new gene model Protein ID 56768 is not part of the optimized gene catalog published in [21])	no	no
Phatr2a_47657	PDZ1	Cytoplasmic membrane, accumulation	GFP fusion [41]	no	yes
Phatr2a_42538	PIP1	Cytoplasmic membrane, endocytic vesicles	GFP fusion [41] + GFP fusion [52], protein ID in Publication [52] is 31553	no	yes
Phatr2a_47562	PDZ2	Cytoplasmic membrane, uniform distribution	GFP fusion [41] + GFP fusion [52]	no	yes
56467	SBPase	Cytosol	GFP fusion [23]	yes	yes
48220	Sec24-like	Cytosol	GFP fusion [47]	yes	yes

Protein ID	Name	Location	Method, References, Comments	Contained in reference set of [21]?	Used for statistics?
Phatr2a_23598	Gapdh3	Cytosol	GFP fusion [50]	no	yes
Phatr2a_45997	PK	Cytosol	GFP fusion [50]	no	yes
56801	PGM	Cytosol	GFP fusion [50], protein ID in publication is 51298, the gene model Protein ID 56801 is not part of the optimized gene catalog published in [21]	no	yes
Phatr2a_54173	PthRpn10	Cytosol	GFP fusion [57], protein ID in publication is 25989	no	yes
Phatr2a_35532	Pthbeta7	Cytosol	GFP fusion [57], protein ID in publication is 35532	no	yes
51055	NTR	Cytosol	GFP fusion [63]	yes	yes
29014	FBA3	Cytosol	YFP fusion [2]	yes	yes
Phatr2a_21988	PPDK (pyruvate phosphate dikinase)	Cytosol (LD)	GFP fusion [15] + Mass spectrometric peptide mapping of proteins from isolated lipid droplets [39], sequence identical to UniProt B7G585; counted as Cytosol, the identification in LD fraction is considered a contamination	no	yes
23247	FBP	Cytosol (LD)	GFP fusion [23] + mass spectrometric peptide mapping of proteins from isolated lipid droplets [39], here counted as cytosolic, the occurence in the lipid droplet sample is counted as contamination, sequence identical to UniProt B7GAR0	yes	yes
Phatr2a_54738	TPI	Cytosol (LD)	GFP fusion [50] + Mass spectrometric peptide mapping of proteins from isolated lipid droplets [39], sequence identical to UniProt B7G3C1; counted as Cytosol, the identification in LD fraction is considered a contamination	no	yes
Phatr2a_49098	PK	Cytosol (LD)	GFP fusion [50] + Mass spectrometric peptide mapping of proteins from isolated lipid droplets [39], sequence identical to UniProt B7G9H4; counted as Cytosol, the identification in LD fraction is considered a contamination	no	yes
Phatr2a_56445	PK	Cytosol (LD)	GFP fusion [50] + Mass spectrometric peptide mapping of proteins from isolated lipid droplets [39], UniProt ID in publication is B7G9G7; counted as Cytosol, the identification in LD fraction is considered a contamination	no	yes
Phatr2a_56383	PGK	Cytosol (LD)	GFP fusion [50], ID in publication is 51125 + Mass spectrometric peptide mapping of proteins from isolated lipid droplets [39], UniProt ID in publication is B7G6H0; counted as Cytosol, the identification in LD fraction is considered a contamination	no	yes
56471	TrxH	Cytosol (LD)	GFP fusion [63] + Mass spectrometric peptide mapping of proteins from isolated lipid droplets [39], UniProt ID in publication [39] is B7G0C9; counted as Cytosol, the identification in LD fraction is considered a contamination	yes	yes
42447	FBA4	Cytosol (LD)	YFP fusion [2] + mass spectrometric peptide mapping of proteins from isolated lipid droplets [39], here counted as Cytosol, the occurence in the lipid droplet sample is counted as contamination, sequence identical to UniProt B7FRC1	yes	yes
Phatr2a_56468	ENO	Cytosol + Pl	GFP fusion, diverging results between full length and pre-sequence fusion constructs, start Methionine from GFP was not included in the pre-sequence construct [50], not used for statistics due to unclear location	no	no

Protein ID	Name	Location	Method, References, Comments	Contained in reference set of [21]?	Used for statistics?
Phatr2a_20490	Pt_ArgRS2	dually targeted: Plastid and Mitochondria	GFP fusion [17]; not used for statistics due to dual targeting, ID in Publication is 36013	no	no
56813	Pt_AsnRS2	dually targeted: Plastid and Mitochondria	GFP fusion [17]; not used for statistics due to dual targeting, ID in Publication is 42274. (The new gene model Protein ID 56813 is not part of the optimized gene catalog published in [21])	no	no
Phatr2a_33266	PtNapi1	Endomembrane system (not BLS or plastid)	GFP fusion [?]	no	yes
Phatr2a_48970	PtPhos5	Endomembrane system (not BLS or plastid)	GFP fusion [?]	no	yes
Phatr2a_45757	PtPhos6	Endomembrane system (not BLS or plastid)	GFP fusion [?]	no	yes
Phatr2a_45174	PtPhos7	Endomembrane system (not BLS or plastid)	GFP fusion [?]	no	yes
Phatr2a_48811	PtVtc1	Endomembrane system (not BLS or plastid)	GFP fusion [?]	no	yes
Phatr2a_23832	PtPho4	Endomembrane system (not BLS or plastid)	GFP fusion [?], protein ID in publication is 23830	no	yes
Phatr2a_48717	ptsRhom1	Endomembrane system (not BLS or plastid)	GFP fusion [36]	no	yes
Phatr2a_50019	PtVtc4	Endomembrane system (not BLS or plastid) (LD)	GFP fusion [?] + Mass spectrometric peptide mapping of proteins from isolated lipid droplets [39], UniProt ID in publication is B7GCM8; counted as Endomembrane system, the identification in LD fraction is considered a contamination	no	yes
Phatr2a_48538	PtVtc3	Endomembrane system in defined foci (not BLS or plastid)	GFP fusion + immuno electron microscopy with GFP antibody [?]	no	yes
48518	putative zink transporter	ER	GFP fusion [11]	yes	yes
49272	unknown protein	ER	GFP fusion [11]	yes	yes
37614	hDer1-2	ER	GFP fusion [25, 41]	yes	yes
Phatr2a_19450	protein disulfideisomerase (PDI)	ER	GFP fusion [25] + mRuby3 fusion [42], protein ID in publications: 44937	no	yes
34592	CPF2	ER	GFP fusion [47]	yes	yes
50476	Glutathione peroxidase	ER	GFP fusion [47]	yes	yes
44172	HAP	ER	GFP fusion [47]	yes	yes
49840	ORF387	ER	GFP fusion [47]	yes	yes

Protein ID	Name	Location	Method, References, Comments	Contained in reference set of [21]?	Used for statistics?
17683	SybA	ER	GFP fusion [47]	yes	yes
45601	SybD	ER	GFP fusion [47]	yes	yes
56732	Rab1b	ER	GFP fusion [47], protein ID in reference by Moog et al.[47]: 41867	yes	yes
44080	STK4	ER	GFP fusion [47], protein ID in reference by Moog et al.[47]: 33437	yes	yes
48050	Fru	ER	GFP fusion [47], protein ID in reference by Moog et al.[47]: 38415	yes	yes
55029	CA-III	ER	GFP fusion [59]	yes	yes
54145	TPT1	ER	GFP fusion + self-assembling GFP analyses [46], model name in publication is estExt__Phatr1__ua__kg.C__chr__20080, ID in publication is 54145	no	yes
54246	Bip	ER (LD)	GFP fusion [3, 32] + Mass spectrometric peptide mapping of proteins from isolated lipid droplets [39], UniProt ID in publication [39] is B7FUB7; counted as ER, the identification in LD fraction is considered a contamination. (Protein ID 20331 is not part of the optimized gene catalog published in [21])	yes	yes
Phatr2a__43157	MIP1b	ER membrane (cER, nuclear envelope, host ER)	GFP fusion [41]	no	yes
Phatr2a__20755	MIP1a	ER membrane (cER, nuclear envelope, host ER)	GFP fusion + mRFP fusion + immuno electron microscopy with GFP antibody [41]	no	yes
Phatr2a__56443	Sec61alpha	ER membrane (cER, nuclear envelope, host ER) (LD)	GFP fusion [41] + Mass spectrometric peptide mapping of proteins from isolated lipid droplets [39], UniProt ID in publication is B7GD39; counted as ER membrane, the identification in LD fraction is considered a contamination	no	yes
42574	CA-VII/sαCA-2	ER/BLS	GFP fusion [47, 59], divergent results between the two studies, clearly no plastid protein; counted as "not plastid" by Gruber et al. [21], not counted in this study due to ambiguous results regarding ER/PPC location	yes	no
Phatr2a__43320	TPT8	ER/BLS	GFP fusion [46], not used for statistics due to unclear location, model name in publication is estExt__fgenesh1__pg.C__chr__20047	no	no
54251	CA-VI/αCA-2/canonic anhydrase type VI	ER/Extracellular (not Pl or BLS)	GFP fusion [47] + GFP fusion [59] + Extraction of extracellular proteins and nanoLC-MS/MS analysis [9]	yes	yes
Phatr2a__40052	PtCDA	ER/Golgi (varies through cell cycle)	GFP fusion [54]; not used for statistics due to unclear/variable location	no	no
Phatr2a__39604	endo-1,3-beta-D-glucosidase	Extracellular	Extraction of extracellular proteins and nanoLC-MS/MS analysis [9]	no	yes
Phatr2a__49571	LRR-frustulin	Extracellular	Extraction of extracellular proteins and nanoLC-MS/MS analysis [9]	no	yes
Phatr2a__35777	LRR-protein	Extracellular	Extraction of extracellular proteins and nanoLC-MS/MS analysis [9]	no	yes
Phatr2a__45682	LRR-protein	Extracellular	Extraction of extracellular proteins and nanoLC-MS/MS analysis [9]	no	yes

Protein ID	Name	Location	Method, References, Comments	Contained in reference set of [21]?	Used for statistics?
Phatr2a_56342	M6 metalloprotease	Extracellular	Extraction of extracellular proteins and nanoLC-MS/MS analysis [9]	no	yes
Phatr2a_44309	mucin-like	Extracellular	Extraction of extracellular proteins and nanoLC-MS/MS analysis [9]	no	yes
Phatr2a_48735	S8A type b protease	Extracellular	Extraction of extracellular proteins and nanoLC-MS/MS analysis [9]	no	yes
Phatr2a_20638	superoxide dismutase	Extracellular	Extraction of extracellular proteins and nanoLC-MS/MS analysis [9]	no	yes
Phatr2a_48378	W-rich domain protein	Extracellular	Extraction of extracellular proteins and nanoLC-MS/MS analysis [9]	no	yes
22142	epsilon frustulin	Extracellular	Extraction of extracellular proteins and nanoLC-MS/MS analysis [9], ID in publication is 22142, not used for statistics because N-terminus is identical to 38418	no	no
Phatr2a_42501	mucin-like	Extracellular	Extraction of extracellular proteins and nanoLC-MS/MS analysis [9], ID in publication is 31502	no	yes
Phatr2a_43495	Cna B cell surface protein	Extracellular	Extraction of extracellular proteins and nanoLC-MS/MS analysis [9], ID in publication is 32714	no	yes
Phatr2a_44659	mucin-like	Extracellular	Extraction of extracellular proteins and nanoLC-MS/MS analysis [9], ID in publication is 34137	no	yes
Phatr2a_45068	W-rich domain protein	Extracellular	Extraction of extracellular proteins and nanoLC-MS/MS analysis [9], ID in publication is 34674	no	yes
Phatr2a_45797	mucin-like	Extracellular	Extraction of extracellular proteins and nanoLC-MS/MS analysis [9], ID in publication is 35593	no	yes
Phatr2a_47162	mucin-like	Extracellular	Extraction of extracellular proteins and nanoLC-MS/MS analysis [9], ID in publication is 37302	no	yes
38416	epsilon frustulin	Extracellular	Extraction of extracellular proteins and nanoLC-MS/MS analysis [9], ID in publication is 38416	no	yes
38418	epsilon frustulin	Extracellular	Extraction of extracellular proteins and nanoLC-MS/MS analysis [9], ID in publication is 38418, identical N-terminus to 22142	no	yes
Phatr2a_48493	S8A type a protease	Extracellular	Extraction of extracellular proteins and nanoLC-MS/MS analysis [9], ID in publication is 38969	no	yes
Phatr2a_48730	S1C protease	Extracellular	Extraction of extracellular proteins and nanoLC-MS/MS analysis [9], ID in publication is 39272	no	yes
Phatr2a_49059	l13 serine protease inhibitor	Extracellular	Extraction of extracellular proteins and nanoLC-MS/MS analysis [9], ID in publication is 39690	no	yes
Phatr2a_50592	W-rich domain proton	Extracellular	Extraction of extracellular proteins and nanoLC-MS/MS analysis [9], ID in publication is 41587	no	yes
Phatr2a_56409	S1A protease	Extracellular	Extraction of extracellular proteins and nanoLC-MS/MS analysis [9], ID in publication is 44903	no	yes

Protein ID	Name	Location	Method, References, Comments	Contained in reference set of [21]?	Used for statistics?
45709	iron starvation induced protein	Extracellular	Extraction of extracellular proteins and nanoLC-MS/MS analysis [9], ID in publication is 45709	no	yes
51797	alpha-3 frustulin	Extracellular	Extraction of extracellular proteins and nanoLC-MS/MS analysis [9], ID in publication is 51797, not used for statistics because N-terminus is identical to Phatr2a_56359	no	no
52157	laminarinase	Extracellular	Extraction of extracellular proteins and nanoLC-MS/MS analysis [9], ID in publication is 52157, not used for statistics because N-terminus is identical to 54681	no	no
Phatr2a_56359	alpha-3 frustulin	Extracellular	Extraction of extracellular proteins and nanoLC-MS/MS analysis [9], N-terminus identical to 51797	no	yes
18793	unnamed	Extracellular	Mass spectrometric peptide mapping of extracellular proteins [8]	yes	yes
46875	unnamed	Extracellular	Mass spectrometric peptide mapping of extracellular proteins [8]	yes	yes
47165	unnamed	Extracellular	Mass spectrometric peptide mapping of extracellular proteins [8]	yes	yes
55817	unnamed	Extracellular	Mass spectrometric peptide mapping of extracellular proteins [8]	yes	yes
56715	unnamed	Extracellular	Mass spectrometric peptide mapping of extracellular proteins [8]	yes	yes
56716	unnamed	Extracellular	Mass spectrometric peptide mapping of extracellular proteins [8]	yes	yes
45679	8-LRR protein	Extracellular	Mass spectrometric peptide mapping of extracellular proteins [8] + Extraction of extracellular proteins and nanoLC-MS/MS analysis [9]	yes	yes
56345	unknown cell surface protein	Extracellular	Mass spectrometric peptide mapping of extracellular proteins [8] + Extraction of extracellular proteins and nanoLC-MS/MS analysis [9], ID in publication [9] is 39858	yes	yes
50819	Tkl	Extracellular	Mass spectrometric peptide mapping of extracellular proteins [8], plastidic function and prediction result, might be an intracellular contaminant identified in the study by Bruckner et al. [8]	yes	yes
Phatr2a_47612	HASP1 (phytase-like)	Extracellular	SDS-PAGE of secreted proteins and mass spectrometry [**?**] (named PtPhos2) + SDS-PAGE of secreted proteins followed by LC-MS/MS analysis + GFP fusion [14] (ID in publication [14] is PHATRDRAFT_47612, Uniprot ID B7G4A0) + Extraction of extracellular proteins and nanoLC-MS/MS analysis [9]	no	yes
Phatr2a_49678	Alkaline phosphatase	Extracellular	SDS-PAGE of secreted proteins followed by LC-MS/MS analysis [14] (ID in Publication [14] is PHATRDRAFT_49678 (Uniprot ID B7GBF3)) + [**?**] (named PtPhos1) + Extraction of extracellular proteins and nanoLC-MS/MS analysis [9] (ID in Publication is 40436)	no	yes
Phatr2a_43513	Predictcd protein	Extracellular	SDS-PAGE of secreted proteins followed by LC-MS/MS analysis [14] (ID in Publications is PHATRDRAFT_43513 (Uniprot ID B7FSH1)) + Extraction of extracellular proteins and nanoLC-MS/MS analysis [9] (ID in Publication is 32734)	no	yes
Phatr2a_46677	Predicted protein	Extracellular	SDS-PAGE of secreted proteins followed by LC-MS/MS analysis [14], ID in Publication is PHATR_46677 (Uniprot ID B5Y3F2)	no	yes

Protein ID	Name	Location	Method, References, Comments	Contained in reference set of [21]?	Used for statistics?
Phatr2a_54681	Endo-1,3-beta-glucanase	Extracellular	SDS-PAGE of secreted proteins followed by LC-MS/MS analysis [14], ID in Publication is PHATRDRAFT_54681 (Uniprot ID B7G259), N-terminus identical to 52157	no	yes
Phatr2a_44488	unknown cell surface protein	Extracellular (LD)	Extraction of extracellular proteins and nanoLC-MS/MS analysis [9], ID in publication is 33940 + Mass spectrometric peptide mapping of proteins from isolated lipid droplets [39], sequence identical to UniProt B7FUA4; counted as Extracellular, the identification in LD fraction is considered a contamination	no	yes
Phatr2a_48054	Fru2	Extracellular space	GFP fusion [41]	no	yes
Phatr2a_45496	XylT	Medial Golgi	GFP fusion + mRFP fusion [41]	no	yes
Phatr2a_54834	MDH2 (malate dehydrogenase 2)	Mitochondria	GFP fusion [15]	no	yes
Phatr2a_56501	ME1 (NAD-dependent malic enzyme 1)	Mitochondria	GFP fusion [15]	no	yes
56769	PEPC2 (phosphoenolpyruvate carboxylase 2)	Mitochondria	GFP fusion [15], the new gene model Protein ID 56768 is not part of the optimized gene catalog published in [21]	no	yes
56752	PGP_2	Mitochondria	GFP fusion [47], protein ID in reference by Moog et al.[47]: 22127	yes	yes
47685	FolC	Mitochondria	GFP fusion [47], protein ID in reference by Moog et al.[47]: 37961	yes	yes
55162	Ank5	Mitochondria	GFP fusion [47], protein ID in reference by Moog et al.[47]: 41153	yes	yes
Phatr2a_48983	PGK	Mitochondria	GFP fusion [50]	no	yes
Phatr2a_33839	PGM	Mitochondria	GFP fusion [50]	no	yes
Phatr2a_35164	PGM	Mitochondria	GFP fusion [50]	no	yes
56172	PK	Mitochondria	GFP fusion [50]	no	yes
Phatr2a_49002	PK	Mitochondria	GFP fusion [50]	no	yes
Phatr2a_17964	PGM	Mitochondria	GFP fusion [50], ID in publication is 43253	no	yes
20030	CA-VIII	Mitochondria	GFP fusion [59]	yes	yes
31720	TrxO	Mitochondria	GFP fusion [63]	yes	yes
25308	TPI/GapC3	Mitochondria	Immuno electron microscopy with GapC3 antibody [40] + GFP fusion [50], GenBank AF063804	yes	yes
Phatr2a_18878	Phatr2a_18878	Mitochondria	YFP Fusion [29], ID in Publication is Phatr3_J11014	no	yes
56618	unCPS	Mitochondria	YFP fusion and immuno electron microscopy with GFP antibody [1]	yes	yes

Protein ID	Name	Location	Method, References, Comments	Contained in reference set of [21]?	Used for statistics?
Phatr2a_30519	PYC1 (pyruvate carboxylase 1)	Mitochondria (LD)	GFP fusion [15] + Mass spectrometric peptide mapping of proteins from isolated lipid droplets [39], sequence identical to UniProt B7GBG1; counted as Mitochondria, the identification in LD fraction is considered a contamination	no	yes
Phatr2a_20352	ME2 (NADP-dependent malic enzyme 2)	Mitochondria (LD)	GFP fusion [15], protein ID in publication is 27477 + Mass spectrometric peptide mapping of proteins from isolated lipid droplets [39], UniProt ID in publication [39] is B7FZD7, has 67 residues of N-terminal extension (MRTPNRRKYPLGGLTSWIFT-VAAILSNPPR + 37 aa); counted as Mitochondria, the identification in LD fraction is considered a contamination	no	yes
Phatr2a_24196	MDH1 (malate dehydrogenase 1)	Mitochondria (LD)	GFP fusion [15], protein ID in publication is 51297 + Mass spectrometric peptide mapping of proteins from isolated lipid droplets [39], sequence identical to UniProt B7GEG9; counted as Mitochondria, the identification in isolated LD is considered a contamination	no	yes
Phatr2a_56387	PEPCK (phosphoenolpyruvate carboxykinase)	Mitochondria (LD)	GFP fusion [15], protein ID in publication is 55018 + Mass spectrometric peptide mapping of proteins from isolated lipid droplets [39], UniProt ID in publication is B7GA05; counted as Mitochondria, the identification in LD fraction is considered a contamination	no	yes
Phatr2a_26432	GDC-L	Mitochondria (LD)	GFP fusion [41] + Mass spectrometric peptide mapping of proteins from isolated lipid droplets [39], sequence identical to UniProt B7FVM3; counted as Mitochondria, the identification in LD fraction is considered a contamination	no	yes
25127	NDK3	Mitochondria (LD)	GFP fusion [47] + mass spectrometric peptide mapping of proteins from isolated lipid droplets [39], here counted as mitochondiral, the occurence in the lipid droplet sample is counted as contamination. Protein ID in reference by Moog et al.[47]: 18001, sequence identical to UniProt B7FR80	yes	yes
20349	GLR/Gsr2	Mitochondria (LD)	GFP fusion [47] + Mass spectrometric peptide mapping of proteins from isolated lipid droplets [39], UniProt ID in publication is B7FZC3, has 81 residues of N-terminal extension (MLRTVMGFCFAIAASFVSRRTPIMVAASSS + 51 aa); counted as Mitochondria, the identification in LD fraction is considered a contamination	yes	yes
47492	Tom70	Mitochondria (LD)	GFP fusion [47] + mRuby3 fusion [42] + mass spectrometric peptide mapping of proteins from isolated lipid droplets [39], here counted as mitochondiral, the occurence in the lipid droplet sample is counted as contamination. sequence identical to UniProt B7G3J4, protein ID in reference by Moog et al.[47] is 37716	yes	yes
Phatr2u_1572	ENO	Mitochondria (LD)	GFP fusion [50] + Mass spectrometric peptide mapping of proteins from isolated lipid droplets [39], sequence identical to UniProt B7S3N7; counted as Mitochondria, the identification in LD fraction is considered a contamination	no	yes
43174	TPT11	Non-Pl membranes	GFP fusion [46], not used for statistics due to unclear location, model name in publication is estExt_fgenesh1_pg.C_chr_10932, ID in publication is 43174	no	no

Protein ID	Name	Location	Method, References, Comments	Contained in reference set of [21]?	Used for statistics?
Phatr2a_49661	TPT10	Non-Pl membranes	GFP fusion [46], not used for statistics due to unclear location, model name in publication is estExt_fgenesh1_pg.C_chr_230136	no	no
Phatr2a_50485	TPT6	Non-Pl membranes	GFP fusion [46], not used for statistics due to unclear location, model name in publication is estExt_fgenesh1_pg.C_chr_300063	no	no
Phatr2a_45268	TPT5	Non-Pl membranes	GFP fusion [46], not used for statistics due to unclear location, model name in publication is estExt_fgenesh1_pg.C_chr_60290	no	no
56688	AUREO2	Nucleus	GFP fusion [51], ID 56688 is not part of the optimized gene catalog published in [21]	no	yes
49458	AUREO1b	Nucleus	GFP fusion [51], ID in publication is 49458	no	yes
Phatr2a_54360	H2B	Nucleus (LD)	GFP fusion [45] + mRuby3 fusion [42] + Mass spectrometric peptide mapping of proteins from isolated lipid droplets [39], sequence identical to UniProt B7FWR8, H2B-1a (Protein ID 11823) and H2B-1b (Protein ID 54360) are two genes of different loci with identical aa sequence; counted as Nucleus, the identification in isolated LD is considered a contamination	no	yes
49116	AUREO1a	Nucleus + Cytosol	GFP fusion [51], ID in publication is 49116	no	yes
Phatr2a_22418	catalase	Peroxisomes	GFP fusion [18]	no	yes
Phatr2a_17720	long chain acyl-CoA ligase	Peroxisomes	GFP fusion [18]	no	yes
Phatr2a_54478	malate synthase	Peroxisomes	GFP fusion [18]	no	yes
Phatr2a_50623	Pex3	Peroxisomes	GFP fusion [18]	no	yes
Phatr2a_37372	trans-2-enoyl-CoA reductase	Peroxisomes	GFP fusion [18]	no	yes
Phatr2a_41969	3-ketoacyl-CoA thiolase	Peroxisomes	GFP fusion + immuno electron microscopy with GFP antibody [18]	no	yes
Phatr2a_47516	Pex10	Peroxisomes	GFP fusion + immuno electron microscopy with GFP antibody [18]	no	yes
Phatr2a_47406	Pex16	Peroxisomes	GFP fusion + immuno electron microscopy with GFP antibody [44]	no	yes
Phatr2a_40433	PtNapi2	Plasma membrane	GFP fusion [?]	no	yes
Phatr2a_47667	PtNapi4	Plasma membrane	GFP fusion [?]	no	yes
Phatr2a_49842	PtNapi5	Plasma membrane	GFP fusion [?]	no	yes
Phatr2a_47239	PtNapi3	Plasma membrane + spot (not BLS or plastid)	GFP fusion [?]	no	yes
17265	PtHpi1	Plasma membrane + spot (not BLS or plastid)	GFP fusion [?], the gene model Protein ID 17265 is not part of the optimized gene catalog published in [21]	no	yes

Protein ID	Name	Location	Method, References, Comments	Contained in reference set of [21]?	Used for statistics?
Phatr2a_45959	PtPhos3	Plasma membrane and Endomembrane system (not BLS or plastid)	GFP fusion [?]	no	yes
Phatr2a_47869	PtPhos8	Plasma membrane and Endomembrane system (not BLS or plastid)	GFP fusion [?]	no	yes
Phatr2a_17936	Vps29	Transgolgi network	GFP fusion + mRFP fusion [41]	no	yes
Phatr2a_20139	Vps26	Transgolgi network (LD)	GFP fusion [41], ID in publication is 20140 + Mass spectrometric peptide mapping of proteins from isolated lipid droplets [39], UniProt ID in publication is B7FYJ6, has 117 residues of N-terminal extension (MNVGSLLGALTGSGGPAVEIRLTPPSSQDI + 87 aa); counted as Transgolgi network, the identification in LD fraction is considered a contamination	no	yes
Phatr2a_43812	PGM	Unclear, cytosol + ER or mitochondria	GFP fusion [50], not used for statistics due to unclear location	no	no
Phatr2a_19075	PGM	Unclear, mitochondria or ER	GFP fusion [50], not used for statistics due to unclear location, ID in publication is 26201	no	no
19556	ABC3	Vacuolar membrane	GFP fusion [41] + GFP fusion [52], ID in publication is 19556	no	yes
bd1370	TIP1	Vacuolar membrane	GFP fusion [41], ID bd1370 is not part of the optimized gene catalog published in [21]	no	yes
Phatr2a_19587	PtVpt1	Vacuole	GFP fusion [?], protein ID in publication is 19586	no	yes
Phatr2a_50238	TGS1	Vacuole	GFP fusion [28]	no	yes
56509	TGS2	Vacuole	GFP fusion [28], ID 56509 is not part of the optimized gene catalog published in [21]	no	yes
56808	PtBGS	Vacuole	GFP fusion [27], ID 56808 is not part of the optimized gene catalog published in [21]	no	yes
Phatr2a_47599	CBS	Vacuole	GFP fusion [52]	no	yes
Phatr2a_30620	VCT3	Vacuole	GFP fusion [52]	no	yes
Phatr2a_35739	VTC2	Vacuole	GFP fusion + immuni electron microscopy with GFP antibody [52]	no	yes
Phatr2a_49793	beta-GLYC1	Vacuole (LD)	GFP fusion [52] + Mass spectrometric peptide mapping of proteins from isolated lipid droplets [39], sequence identical to UniProt B7GBX3; counted as Vacuole, the identification in LD fraction is considered a contamination	no	yes
Phatr2a_18466	Predicted protein	LD	Mass spectrometric peptide mapping of proteins from isolated lipid droplets [39], UniProt ID in publication is B5Y4I5, has 629 residues of N-terminal extension (MGNIPTGMEGDGSPSNDKNKDSKEEDDPSK + 599 aa).	no	no
Phatr2a_18582	Predicted protein	LD	Mass spectrometric peptide mapping of proteins from isolated lipid droplets [39], UniProt ID in publication is B5Y4Z9, has 67 residues of N-terminal extension (MITGSLRKLGRSTAHSATTFFVSTTRSTFR + 37 aa).	no	no

Protein ID	Name	Location	Method, References, Comments	Contained in reference set of [21]?	Used for statistics?
Phatr2a_18585	Predicted protein	LD	Mass spectrometric peptide mapping of proteins from isolated lipid droplets [39], sequence identical to UniProt B5Y501.	no	no
Phatr2a_18595	Predicted protein	LD	Mass spectrometric peptide mapping of proteins from isolated lipid droplets [39], UniProt ID in publication is B5Y512, has 25 residues of N-terminal extension (MPRHKILLEKRKLMRITSKLTVLLN).	no	no
Phatr2a_18665	Serine hydroxymethyl-transferase	LD	Mass spectrometric peptide mapping of proteins from isolated lipid droplets [39], sequence identical to UniProt B5Y594.	no	no
Phatr2a_18707	Phosphoribulokinase	LD	Mass spectrometric peptide mapping of proteins from isolated lipid droplets [39], sequence identical to UniProt B5Y5F0.	no	no
Phatr2a_18745	UDP-glucose 6-dehydrogenase	LD	Mass spectrometric peptide mapping of proteins from isolated lipid droplets [39], sequence identical to UniProt B5Y5J6.	no	no
Phatr2a_18829	Predicted protein (Fragment)	LD	Mass spectrometric peptide mapping of proteins from isolated lipid droplets [39], UniProt ID in publication is B5Y5U1, has 438 residues of N-terminal extension (MKYLVAVTTLLSTVSVSAFVPQKAAFGALT + 408 aa), and 166 positions of C-terminal extension (136 aa + VIKADGTAVKYESEERTVEAFKKFMEKTL*).	no	no
Phatr2a_18985	Predicted protein (Fragment)	LD	Mass spectrometric peptide mapping of proteins from isolated lipid droplets [39], UniProt ID in publication is B7FU58, has 70 residues of N-terminal extension (MKLASATAFAAFLVTASAFSPSMMARSTTK + 40 aa), and 56 positions of C-terminal extension (26 aa + DDLDGTNGDSDSNEDPLKNVSDSLDSLLS*).	no	no
Phatr2a_19004	Proteasome subunit alpha type	LD	Mass spectrometric peptide mapping of proteins from isolated lipid droplets [39], sequence identical to UniProt B7FU90.	no	no
Phatr2a_19025	Predicted protein	LD	Mass spectrometric peptide mapping of proteins from isolated lipid droplets [39], sequence identical to UniProt B7FUB2.	no	no
Phatr2a_19030	Predicted protein	LD	Mass spectrometric peptide mapping of proteins from isolated lipid droplets [39], sequence identical to UniProt B7FUB3.	no	no
Phatr2a_19114	Proteasome endopeptidase complex	LD	Mass spectrometric peptide mapping of proteins from isolated lipid droplets [39], UniProt ID in publication is B7FUL2, has 5 residues of N-terminal extension (MSGVT).	no	no
Phatr2a_19117	GDT1 family protein (Fragment)	LD	Mass spectrometric peptide mapping of proteins from isolated lipid droplets [39], UniProt ID in publication is B7FUM2, has 74 residues of N-terminal extension (MPVSHFSRTFFALGIVAVVAASVADDWFKT + 44 aa), and 6 positions of C-terminal extension (FFFES*).	no	no
Phatr2a_19182	Cysteine desulfurase	LD	Mass spectrometric peptide mapping of proteins from isolated lipid droplets [39], UniProt ID in publication is B7FUS8, has 82 residues of N-terminal extension (MLCSAGRRLPRRLLPKHYQRTPLSSAAAQN + 52 aa).	no	no

Protein ID	Name	Location	Method, References, Comments	Contained in reference set of [21]?	Used for statistics?
Phatr2a_19214	Predicted protein	LD	Mass spectrometric peptide mapping of proteins from isolated lipid droplets [39], UniProt ID in publication is B7FUX2, has 436 residues of N-terminal extension (MNYEVYDDDANVIETVSDEELHEEMDTEQQ + 406 aa).	no	no
Phatr2a_19217	Predicted protein (Fragment)	LD	Mass spectrometric peptide mapping of proteins from isolated lipid droplets [39], UniProt ID in publication is B7FUX3, has 118 residues of N-terminal extension (MGSTNSRPTQTQGSDREEESSVCNTPCFFF + 88 aa), and 61 positions of C-terminal extension (31 aa + YQPPARKYAAAPDRTPPTSLGPSIRKTSI*).	no	no
Phatr2a_19261	Predicted protein (Fragment)	LD	Mass spectrometric peptide mapping of proteins from isolated lipid droplets [39], UniProt ID in publication is B7FVE6, has 10 positions of C-terminal extension (ATQQASQQS*).	no	no
Phatr2a_19341	Proteasome subunit beta	LD	Mass spectrometric peptide mapping of proteins from isolated lipid droplets [39], sequence identical to UniProt B7FVS5.	no	no
Phatr2a_19361	Elongation factor G, mitochondrial	LD	Mass spectrometric peptide mapping of proteins from isolated lipid droplets [39], UniProt ID in publication is B7FVU7, has 33 residues of N-terminal extension (MVSFGFSGAVALLASLSVVNAFAPAQFGLV + 3 aa).	no	no
Phatr2a_19413	Predicted protein	LD	Mass spectrometric peptide mapping of proteins from isolated lipid droplets [39], sequence identical to UniProt B7FVY3.	no	no
Phatr2a_19490	Predicted protein (Fragment)	LD	Mass spectrometric peptide mapping of proteins from isolated lipid droplets [39], UniProt ID in publication is B7FV13, has 2 residues of N-terminal extension (MA), and 16 positions of C-terminal extension (NIEIRRDALAIDTKV*).	no	no
Phatr2a_19552	Predicted protein	LD	Mass spectrometric peptide mapping of proteins from isolated lipid droplets [39], sequence identical to UniProt B7FW92.	no	no
Phatr2a_19568	RuvB-like helicase	LD	Mass spectrometric peptide mapping of proteins from isolated lipid droplets [39], sequence identical to UniProt B7FWC9.	no	no
Phatr2a_19661	Predicted protein	LD	Mass spectrometric peptide mapping of proteins from isolated lipid droplets [39], sequence identical to UniProt B7FWP5.	no	no
Phatr2a_19690	Peptidylprolyl isomerase	LD	Mass spectrometric peptide mapping of proteins from isolated lipid droplets [39], sequence identical to UniProt B7FWT8.	no	no
Phatr2a_19708	Fumarate hydratase	LD	Mass spectrometric peptide mapping of proteins from isolated lipid droplets [39], sequence identical to UniProt B7FWX5.	no	no
Phatr2a_19785	Predicted protein	LD	Mass spectrometric peptide mapping of proteins from isolated lipid droplets [39], UniProt ID in publication is B7FX87, has 19 residues of N-terminal extension (MATAAVVNPVPFSDPDSSK).	no	no
Phatr2a_19805	Predicted protein	LD	Mass spectrometric peptide mapping of proteins from isolated lipid droplets [39], sequence identical to UniProt B7FXB1.	no	no

Protein ID	Name	Location	Method, References, Comments	Contained in reference set of [21]?	Used for statistics?
Phatr2a_19828	Ubiquitin carboxyl-terminal hydrolase	LD	Mass spectrometric peptide mapping of proteins from isolated lipid droplets [39], UniProt ID in publication is B7FXE5, has 29 residues of N-terminal extension (MVSASLAIALWIAYRVFLDSCPPSLENRT).	no	no
Phatr2a_19971	Enoyl-CoA hydratase	LD	Mass spectrometric peptide mapping of proteins from isolated lipid droplets [39], UniProt ID in publication is B7FXY5, has 134 residues of N-terminal extension (MLASRRAFAHSIVVTRRVGPHGAGSSVGRL + 104 aa).	no	no
Phatr2a_20047	Predicted protein (Fragment)	LD	Mass spectrometric peptide mapping of proteins from isolated lipid droplets [39], UniProt ID in publication is B7FY79, has 29 residues of N-terminal extension (MMSTSGPPGAAVQGVPQRHRPEDEAMHIT), and 108 positions of C-terminal extension (78 aa + SVPAPYTVPVGGDPSSMISVQYREAPGGF*).	no	no
Phatr2a_20183	Pyruvate dehydrogenase E1 component subunit beta	LD	Mass spectrometric peptide mapping of proteins from isolated lipid droplets [39], sequence identical to UniProt B7FZN6.	no	no
Phatr2a_20227	26S proteasome regulatory subunit RPN11	LD	Mass spectrometric peptide mapping of proteins from isolated lipid droplets [39], sequence identical to UniProt B7FYR8.	no	no
Phatr2a_20342	Synthase of glutamate synthase	LD	Mass spectrometric peptide mapping of proteins from isolated lipid droplets [39], sequence identical to UniProt B7FZB0.	no	no
Phatr2a_20424	Urea transporter	LD	Mass spectrometric peptide mapping of proteins from isolated lipid droplets [39], sequence identical to UniProt B7FZW5.	no	no
Phatr2a_20434	Regulatory proteasome non-atpase subunit 12	LD	Mass spectrometric peptide mapping of proteins from isolated lipid droplets [39], sequence identical to UniProt B7FZY2.	no	no
Phatr2a_20536	Transmembrane 9 superfamily member (Fragment)	LD	Mass spectrometric peptide mapping of proteins from isolated lipid droplets [39], UniProt ID in publication is B7G0A0, has 119 residues of N-terminal extension (MKFLTALCFAVVALSSVPSVAAKKTKTPKP + 89 aa).	no	no
Phatr2a_20623	Prohibitin (Fragment)	LD	Mass spectrometric peptide mapping of proteins from isolated lipid droplets [39], UniProt ID in publication is B7G0J8, has 59 residues of N-terminal extension (MSNQFRDAFQKIAQQAQKAAASGGGGGAGG + 29 aa), and 20 positions of C-terminal extension (LGDTDDKFERSIQSRKGYW*).	no	no
Phatr2a_20626	Succinate–CoA ligase [ADP-forming] subunit alpha, mitochondrial	LD	Mass spectrometric peptide mapping of proteins from isolated lipid droplets [39], UniProt ID in publication is B7G0K7, has 19 residues of N-terminal extension (MIIRHSLVKKIAAQAGRRE).	no	no
Phatr2a_20641	Vesicle-fusing ATPase (Fragment)	LD	Mass spectrometric peptide mapping of proteins from isolated lipid droplets [39], UniProt ID in publication is B7G0L9, has 224 residues of N-terminal extension (MFKVKLQVGNLPSNRLALTNKVYVSSNTAA + 194 aa).	no	no
Phatr2a_20677	Predicted protein	LD	Mass spectrometric peptide mapping of proteins from isolated lipid droplets [39], sequence identical to UniProt B7G0Q2.	no	no

Protein ID	Name	Location	Method, References, Comments	Contained in reference set of [21]?	Used for statistics?
Phatr2a_20684	Predicted protein (Fragment)	LD	Mass spectrometric peptide mapping of proteins from isolated lipid droplets [39], UniProt ID in publication is B7G0Q6, has 23 residues of N-terminal extension (MLSSIFSKSMLLRSAVTSSSSTS), and 68 positions of C-terminal extension (38 aa + GGFGGGYGDSSGGGGYGNFDMDDNDYEKK*).	no	no
Phatr2a_20774	Glutamate-1-semialdehyde 2,1-aminomutase	LD	Mass spectrometric peptide mapping of proteins from isolated lipid droplets [39], sequence identical to UniProt B7G134.	no	no
Phatr2a_20787	Predicted protein	LD	Mass spectrometric peptide mapping of proteins from isolated lipid droplets [39], sequence identical to UniProt B7G150.	no	no
Phatr2a_20805	Predicted protein (Fragment)	LD	Mass spectrometric peptide mapping of proteins from isolated lipid droplets [39], UniProt ID in publication is B7G170, has 195 residues of N-terminal extension (MSTFSVPFVSGATDSWGPPAIVTKDADNAD + 165 aa).	no	no
Phatr2a_20875	L-ascorbate peroxidase (Fragment)/Predicted protein (Fragment)	LD	Mass spectrometric peptide mapping of proteins from isolated lipid droplets [39], UniProt IDs in publication are B7G1K0 and B7G1J9, B7G1J9 has 400 residues of N-terminal extension (MANAHYADEFYDNAARAEVSRAIVNKKVNV + 370 aa), and 123 positions of C-terminal extension (93 aa + DKTIRNKEGKSATDLAAANGKDAVVKMLA*), B7G1K0 has 705 residues of N-terminal extension (MANAHYADEFYDNAARAEVSRAIVNKKVNV + 675 aa), and 16 positions of C-terminal extension (LAAANGKDAVVKMLA*).	no	no
Phatr2a_21030	V-type proton ATPase subunit	LD	Mass spectrometric peptide mapping of proteins from isolated lipid droplets [39], sequence identical to UniProt B5Y3H8.	no	no
Phatr2a_21060	Ribose-5-phosphate isomerase	LD	Mass spectrometric peptide mapping of proteins from isolated lipid droplets [39], sequence identical to UniProt B5Y3N7.	no	no
Phatr2a_21081	Predicted protein	LD	Mass spectrometric peptide mapping of proteins from isolated lipid droplets [39], sequence identical to UniProt B5Y3R0.	no	no
Phatr2a_21116	Citrulline–aspartate ligase	LD	Mass spectrometric peptide mapping of proteins from isolated lipid droplets [39], sequence identical to UniProt B5Y3V3.	no	no
Phatr2a_21122	Tubulin beta chain	LD	Mass spectrometric peptide mapping of proteins from isolated lipid droplets [39], sequence identical to UniProt B5Y3W7.	no	no
Phatr2a_21201	UDP-sulfoquinovose synthase, plastid	LD	Mass spectrometric peptide mapping of proteins from isolated lipid droplets [39], sequence identical to UniProt B5Y493.	no	no
Phatr2a_21204	Predicted protein (Fragment)	LD	Mass spectrometric peptide mapping of proteins from isolated lipid droplets [39], UniProt ID in publication is B5Y4A1, has 130 residues of N-terminal extension (MPLCCPPGSWPQLLQSRDQLNAEERSVPER + 100 aa).	no	no
Phatr2a_21323	Predicted protein	LD	Mass spectrometric peptide mapping of proteins from isolated lipid droplets [39], sequence identical to UniProt B7G2B5.	no	no

Protein ID	Name	Location	Method, References, Comments	Contained in reference set of [21]?	Used for statistics?
Phatr2a_21361	Regulatory proteasome non-atpase subunit 1	LD	Mass spectrometric peptide mapping of proteins from isolated lipid droplets [39], UniProt ID in publication is B7G2F7, has 11 residues of N-terminal extension (MAPKDKKAVDS).	no	no
Phatr2a_21430	Predicted protein	LD	Mass spectrometric peptide mapping of proteins from isolated lipid droplets [39], sequence identical to UniProt B7G2T8.	no	no
Phatr2a_21535	Predicted protein	LD	Mass spectrometric peptide mapping of proteins from isolated lipid droplets [39], sequence identical to UniProt B7G312.	no	no
Phatr2a_21592	Diaminopimelate decarboxylase	LD	Mass spectrometric peptide mapping of proteins from isolated lipid droplets [39], sequence identical to UniProt B7G3A2.	no	no
Phatr2a_21659	Predicted protein	LD	Mass spectrometric peptide mapping of proteins from isolated lipid droplets [39], sequence identical to UniProt B7G3G9.	no	no
Phatr2a_21789	T-complex protein 1 subunit gamma	LD	Mass spectrometric peptide mapping of proteins from isolated lipid droplets [39], sequence identical to UniProt B7G4D0.	no	no
Phatr2a_21817	Predicted protein	LD	Mass spectrometric peptide mapping of proteins from isolated lipid droplets [39], UniProt ID in publication is B7G4G2, has 107 residues of N-terminal extension (MRSNGSSVSPAPRSESMAYPKASTSTLVSK + 77 aa).	no	no
Phatr2a_21821	Predicted protein	LD	Mass spectrometric peptide mapping of proteins from isolated lipid droplets [39], sequence identical to UniProt B7G4G4.	no	no
Phatr2a_21845	Pyrophosphate-dependent phosphofructose kinase	LD	Mass spectrometric peptide mapping of proteins from isolated lipid droplets [39], sequence identical to UniProt B7G4J8.	no	no
Phatr2a_21876	Predicted protein	LD	Mass spectrometric peptide mapping of proteins from isolated lipid droplets [39], UniProt ID in publication is B7G4P7, has 18 residues of N-terminal extension (MFGGIPFEHFAHGGGGGG).	no	no
Phatr2a_21882	V-type proton ATPase proteolipid subunit	LD	Mass spectrometric peptide mapping of proteins from isolated lipid droplets [39], sequence identical to UniProt B7G4Q4.	no	no
Phatr2a_22095	Predicted protein	LD	Mass spectrometric peptide mapping of proteins from isolated lipid droplets [39], sequence identical to UniProt B7G5M5.	no	no
Phatr2a_22175	14-3-3-like protein (Fragment)	LD	Mass spectrometric peptide mapping of proteins from isolated lipid droplets [39], UniProt ID in publication is B7G5Y2, has 59 residues of N-terminal extension (MGVLTANGERETAHDLPVPDNRAPRNPTPQ + 29 aa), and 4 positions of C-terminal extension (DKE*).	no	no
Phatr2a_22187	Glycine cleavage system P protein	LD	Mass spectrometric peptide mapping of proteins from isolated lipid droplets [39], sequence identical to UniProt B7G5Z8.	no	no

Protein ID	Name	Location	Method, References, Comments	Contained in reference set of [21]?	Used for statistics?
Phatr2a_22206	Isocitrate dehydrogenase (Fragment)	LD	Mass spectrometric peptide mapping of proteins from isolated lipid droplets [39], UniProt ID in publication is B7G620, has 53 residues of N-terminal extension (MSSLSTLRILHSTAGRRWASYYGIYPKSAA + 23 aa), and 139 positions of C-terminal extension (109 aa + DFEEFTRMLVKMNLAPLLTKKEKEKKPDV*).	no	no
Phatr2a_22277	Predicted protein	LD	Mass spectrometric peptide mapping of proteins from isolated lipid droplets [39], sequence identical to UniProt B7G6E4.	no	no
Phatr2a_22305	Rab GDP dissociation inhibitor	LD	Mass spectrometric peptide mapping of proteins from isolated lipid droplets [39], UniProt ID in publication is B7G6J6, has 18 residues of N-terminal extension (MAEGQGEPKKNLDWLPEG).	no	no
Phatr2a_22357	GLNA, glutamine synthase	LD	Mass spectrometric peptide mapping of proteins from isolated lipid droplets [39], sequence identical to UniProt B7G6Q6.	no	no
Phatr2a_22404	Pyruvate kinase	LD	Mass spectrometric peptide mapping of proteins from isolated lipid droplets [39], sequence identical to UniProt B7G6Z9.	no	no
Phatr2a_22521	GDP-L-fucose synthase	LD	Mass spectrometric peptide mapping of proteins from isolated lipid droplets [39], UniProt ID in publication is B7G7K4, has 17 residues of N-terminal extension (MTAESNETEDARRPSVV).	no	no
Phatr2a_22525	Ubiquitin conjugating enzyme E2	LD	Mass spectrometric peptide mapping of proteins from isolated lipid droplets [39], sequence identical to UniProt B7G7K7.	no	no
Phatr2a_22602	Predicted protein (Fragment)	LD	Mass spectrometric peptide mapping of proteins from isolated lipid droplets [39], UniProt ID in publication is B7G7Z2, has 180 residues of N-terminal extension (MTTTAPKSSDDLAAERQKARDELDAILSKS + 150 aa), and 39 positions of C-terminal extension (9 aa + DAKALYEEATFAAMLETIKRKDDAAHGVS*).	no	no
Phatr2a_22666	Predicted protein	LD	Mass spectrometric peptide mapping of proteins from isolated lipid droplets [39], sequence identical to UniProt B7G853.	no	no
Phatr2a_22774	Predicted protein	LD	Mass spectrometric peptide mapping of proteins from isolated lipid droplets [39], sequence identical to UniProt B7G8J0.	no	no
Phatr2a_22834	Ubiquitin carboxyl-terminal hydrolase (Fragment)	LD	Mass spectrometric peptide mapping of proteins from isolated lipid droplets [39], UniProt ID in publication is B7G8S3, has 11 positions of C-terminal extension (ARELKQKFAT*).	no	no
Phatr2a_22873	Predicted protein	LD	Mass spectrometric peptide mapping of proteins from isolated lipid droplets [39], sequence identical to UniProt B7G8Y4.	no	no
Phatr2a_22887	Predicted protein (Fragment)	LD	Mass spectrometric peptide mapping of proteins from isolated lipid droplets [39], UniProt ID in publication is B7G916, has 144 residues of N-terminal extension (MSGVLRTLLTVTRRTVPTRVTRRCWSANSS + 114 aa).	no	no
Phatr2a_22896	ATPase ASNA1 homolog	LD	Mass spectrometric peptide mapping of proteins from isolated lipid droplets [39], sequence identical to UniProt B7G933.	no	no

Protein ID	Name	Location	Method, References, Comments	Contained in reference set of [21]?	Used for statistics?
Phatr2a_22919	Predicted protein	LD	Mass spectrometric peptide mapping of proteins from isolated lipid droplets [39], sequence identical to UniProt B7G964.	no	no
Phatr2a_23034	Predicted protein	LD	Mass spectrometric peptide mapping of proteins from isolated lipid droplets [39], UniProt ID in publication is B7G9S7, has 406 residues of N-terminal extension (MSLTLKSPVSPAFTNIQWDSLGSLKLTAQE + 376 aa).	no	no
Phatr2a_23079	60S ribosomal protein L7a	LD	Mass spectrometric peptide mapping of proteins from isolated lipid droplets [39], sequence identical to UniProt B7GA10.	no	no
Phatr2a_23084	Predicted protein (Fragment)	LD	Mass spectrometric peptide mapping of proteins from isolated lipid droplets [39], UniProt ID in publication is B7GA12, has 23 residues of N-terminal extension (MTGGASCKAVCQLSSESSGNPSL).	no	no
Phatr2a_23095	Succinate dehydrogenase [ubiquinone] iron-sulfur subunit, mitochondrial	LD	Mass spectrometric peptide mapping of proteins from isolated lipid droplets [39], UniProt ID in publication is B7GA40, has 8 residues of N-terminal extension (MFSCTVTI).	no	no
Phatr2a_23292	L-galactono-1,4-lactone dehydrogenase	LD	Mass spectrometric peptide mapping of proteins from isolated lipid droplets [39], sequence identical to UniProt B7GAW2.	no	no
Phatr2a_23381	Dihydrolipoyllysine-residue succinyltransferase	LD	Mass spectrometric peptide mapping of proteins from isolated lipid droplets [39], UniProt ID in publication is B7GBE7, has 72 residues of N-terminal extension (MLPRVRLSFVAASRRVGTLPTATLASGDRT + 42 aa).	no	no
Phatr2a_23739	CCT-theta	LD	Mass spectrometric peptide mapping of proteins from isolated lipid droplets [39], UniProt ID in publication is B7GCW2, has 23 residues of N-terminal extension (MSSMAYNQAAGLSGMLKEGSRHV).	no	no
Phatr2a_23748	Predicted protein	LD	Mass spectrometric peptide mapping of proteins from isolated lipid droplets [39], sequence identical to UniProt B7GCW6.	no	no
Phatr2a_23788	Phosphoglucomutase	LD	Mass spectrometric peptide mapping of proteins from isolated lipid droplets [39], sequence identical to UniProt B7GD07.	no	no
Phatr2a_23924	Glucose-6-phosphate isomerase	LD	Mass spectrometric peptide mapping of proteins from isolated lipid droplets [39], sequence identical to UniProt B7GDK9.	no	no
Phatr2a_24064	Predicted protein	LD	Mass spectrometric peptide mapping of proteins from isolated lipid droplets [39], UniProt ID in publication is B7GE20, has 40 residues of N-terminal extension (MAKQSVSKAVTAYLGRALRETGAALKHRGE + 10 aa).	no	no
Phatr2a_24089	UTP–glucose-1-phosphate uridylyltransferase	LD	Mass spectrometric peptide mapping of proteins from isolated lipid droplets [39], sequence identical to UniProt B7GE51.	no	no
Phatr2a_24104	Heat shock protein Hsp70	LD	Mass spectrometric peptide mapping of proteins from isolated lipid droplets [39], UniProt ID in publication is B7GE61, has 948 residues of N-terminal extension (MNTTYNERYQYPQPSRSVSIALELVVVVVN + 918 aa).	no	no

Protein ID	Name	Location	Method, References, Comments	Contained in reference set of [21]?	Used for statistics?
Phatr2a_24139	Predicted protein	LD	Mass spectrometric peptide mapping of proteins from isolated lipid droplets [39], UniProt ID in publication is B7GEA9, has 44 residues of N-terminal extension (MRLIRQSVRIAHTLSYFSLNNNKHSFAFAF + 14 aa).	no	no
Phatr2a_24142	Predicted protein	LD	Mass spectrometric peptide mapping of proteins from isolated lipid droplets [39], UniProt ID in publication is B7GEB0, has 59 residues of N-terminal extension (MPVYSVFALLPRIYHRKNRWRLSTFIAIVA + 29 aa).	no	no
Phatr2a_24186	Predicted protein	LD	Mass spectrometric peptide mapping of proteins from isolated lipid droplets [39], sequence identical to UniProt B7GEG3.	no	no
Phatr2a_24274	Predicted protein	LD	Mass spectrometric peptide mapping of proteins from isolated lipid droplets [39], UniProt ID in publication is B7GES5, has 60 residues of N-terminal extension (MIASKSASWIRTGARGAALFVGPRVSTTSP + 30 aa).	no	no
Phatr2a_24474	Proteasome subunit alpha type	LD	Mass spectrometric peptide mapping of proteins from isolated lipid droplets [39], sequence identical to UniProt B7FP06.	no	no
Phatr2a_24772	3-isopropylmalate dehydratase	LD	Mass spectrometric peptide mapping of proteins from isolated lipid droplets [39], sequence identical to UniProt B7FPZ8.	no	no
Phatr2a_24820	Mitochondria-targeted chaperonin	LD	Mass spectrometric peptide mapping of proteins from isolated lipid droplets [39], sequence identical to UniProt B7FQ72.	no	no
Phatr2a_24978	Vacuolar proton pump subunit B	LD	Mass spectrometric peptide mapping of proteins from isolated lipid droplets [39], sequence identical to UniProt B7FQQ8.	no	no
Phatr2a_25417	GDP-mannose 4,6-dehydratase	LD	Mass spectrometric peptide mapping of proteins from isolated lipid droplets [39], sequence identical to UniProt B7FSX7.	no	no
Phatr2a_25577	Predicted protein	LD	Mass spectrometric peptide mapping of proteins from isolated lipid droplets [39], sequence identical to UniProt B5Y4G9.	no	no
Phatr2a_25714	Ribosomal protein L15	LD	Mass spectrometric peptide mapping of proteins from isolated lipid droplets [39], sequence identical to UniProt B5Y502.	no	no
Phatr2a_25752	Transmembrane 9 superfamily member	LD	Mass spectrometric peptide mapping of proteins from isolated lipid droplets [39], sequence identical to UniProt B5Y536.	no	no
Phatr2a_26290	(2R,3S)-2-methylisocitrate dehydratase	LD	Mass spectrometric peptide mapping of proteins from isolated lipid droplets [39], sequence identical to UniProt B7FUR4.	no	no
Phatr2a_26363	Predicted protein	LD	Mass spectrometric peptide mapping of proteins from isolated lipid droplets [39], sequence identical to UniProt B7FUZ0.	no	no
Phatr2a_26382	Predicted protein	LD	Mass spectrometric peptide mapping of proteins from isolated lipid droplets [39], sequence identical to UniProt B7FVE3.	no	no

Protein ID	Name	Location	Method, References, Comments	Contained in reference set of [21]?	Used for statistics?
Phatr2a_26802	Histone H2A	LD	Mass spectrometric peptide mapping of proteins from isolated lipid droplets [39], sequence identical to UniProt B7FWQ2.	no	no
Phatr2a_26921	Succinate–CoA ligase [ADP-forming] subunit beta, mitochondrial	LD	Mass spectrometric peptide mapping of proteins from isolated lipid droplets [39], sequence identical to UniProt B7FXA2.	no	no
Phatr2a_26980	CCT-alpha	LD	Mass spectrometric peptide mapping of proteins from isolated lipid droplets [39], sequence identical to UniProt B7FXH5.	no	no
Phatr2a_27039	Predicted protein	LD	Mass spectrometric peptide mapping of proteins from isolated lipid droplets [39], sequence identical to UniProt B7FXR6.	no	no
Phatr2a_27118	Ubiquitin extension protein 3	LD	Mass spectrometric peptide mapping of proteins from isolated lipid droplets [39], sequence identical to UniProt B7FY02, N-terminus shared with Phatr2a_29166	no	no
Phatr2a_27166	Phosphoglycerate dehydrogenase	LD	Mass spectrometric peptide mapping of proteins from isolated lipid droplets [39], sequence identical to UniProt B7FY73.	no	no
Phatr2a_27385	Predicted protein	LD	Mass spectrometric peptide mapping of proteins from isolated lipid droplets [39], sequence identical to UniProt B7FYY2.	no	no
Phatr2a_27518	Coatomer subunit beta’	LD	Mass spectrometric peptide mapping of proteins from isolated lipid droplets [39], sequence identical to UniProt B7FZS9.	no	no
Phatr2a_27709	Phenylalanyl-tRNA synthetase beta subunit	LD	Mass spectrometric peptide mapping of proteins from isolated lipid droplets [39], sequence identical to UniProt B7G0G3.	no	no
Phatr2a_27821	RNA helicase	LD	Mass spectrometric peptide mapping of proteins from isolated lipid droplets [39], sequence identical to UniProt B7G0R3.	no	no
Phatr2a_27877	Ammonium transporter	LD	Mass spectrometric peptide mapping of proteins from isolated lipid droplets [39], sequence identical to UniProt B7G0Y4.	no	no
Phatr2a_27923	H(+)-transporting two-sector ATPase	LD	Mass spectrometric peptide mapping of proteins from isolated lipid droplets [39], sequence identical to UniProt B7G162.	no	no
Phatr2a_27972	Predicted protein	LD	Mass spectrometric peptide mapping of proteins from isolated lipid droplets [39], sequence identical to UniProt B7G1G4.	no	no
Phatr2a_28068	Beta-ketoacyl-CoA thiolase	LD	Mass spectrometric peptide mapping of proteins from isolated lipid droplets [39], sequence identical to UniProt B5Y3B5.	no	no
Phatr2a_28219	Predicted protein	LD	Mass spectrometric peptide mapping of proteins from isolated lipid droplets [39], sequence identical to UniProt B5Y3S3.	no	no
Phatr2a_28222	Transaldolase	LD	Mass spectrometric peptide mapping of proteins from isolated lipid droplets [39], sequence identical to UniProt B5Y3S6.	no	no

Protein ID	Name	Location	Method, References, Comments	Contained in reference set of [21]?	Used for statistics?
Phatr2a_28562	Predicted protein	LD	Mass spectrometric peptide mapping of proteins from isolated lipid droplets [39], sequence identical to UniProt B7G2U6.	no	no
Phatr2a_28694	Predicted protein	LD	Mass spectrometric peptide mapping of proteins from isolated lipid droplets [39], sequence identical to UniProt B7G365.	no	no
Phatr2a_28737	Elongation factor 1-alpha	LD	Mass spectrometric peptide mapping of proteins from isolated lipid droplets [39], sequence identical to UniProt B7G3C4.	no	no
Phatr2a_29016	2-oxoglutarate dehydrogenase E1 component	LD	Mass spectrometric peptide mapping of proteins from isolated lipid droplets [39], sequence identical to UniProt B7G4T8.	no	no
Phatr2a_29097	Predicted protein	LD	Mass spectrometric peptide mapping of proteins from isolated lipid droplets [39], sequence identical to UniProt B7G544.	no	no
Phatr2a_29166	Ubiquitin	LD	Mass spectrometric peptide mapping of proteins from isolated lipid droplets [39], sequence identical to UniProt B7G5H7, N-terminus shared with Phatr2a_27118	no	no
Phatr2a_29177	Predicted protein	LD	Mass spectrometric peptide mapping of proteins from isolated lipid droplets [39], sequence identical to UniProt B7G5I7.	no	no
Phatr2a_29196	Proliferating cell nuclear antigen	LD	Mass spectrometric peptide mapping of proteins from isolated lipid droplets [39], sequence identical to UniProt B7G5K3.	no	no
Phatr2a_29260	Transketolase	LD	Mass spectrometric peptide mapping of proteins from isolated lipid droplets [39], sequence identical to UniProt B7G5R3.	no	no
Phatr2a_29711	Predicted protein	LD	Mass spectrometric peptide mapping of proteins from isolated lipid droplets [39], sequence identical to UniProt B7G7X7.	no	no
Phatr2a_29885	Predicted protein	LD	Mass spectrometric peptide mapping of proteins from isolated lipid droplets [39], sequence identical to UniProt B7G8H7.	no	no
Phatr2a_29887	Nicotinate phosphoribosyltransferase	LD	Mass spectrometric peptide mapping of proteins from isolated lipid droplets [39], sequence identical to UniProt B7G8I3.	no	no
Phatr2a_29967	Predicted protein	LD	Mass spectrometric peptide mapping of proteins from isolated lipid droplets [39], sequence identical to UniProt B7G8T5.	no	no
Phatr2a_30003	Proteasome endopeptidase complex	LD	Mass spectrometric peptide mapping of proteins from isolated lipid droplets [39], sequence identical to UniProt B7G8Z3.	no	no
Phatr2a_30067	Predicted protein	LD	Mass spectrometric peptide mapping of proteins from isolated lipid droplets [39], sequence identical to UniProt B7G9A4.	no	no
Phatr2a_30139	Predicted protein	LD	Mass spectrometric peptide mapping of proteins from isolated lipid droplets [39], sequence identical to UniProt B7G9L9.	no	no
Phatr2a_30145	Citrate synthase	LD	Mass spectrometric peptide mapping of proteins from isolated lipid droplets [39], sequence identical to UniProt B7G9P5.	no	no

Protein ID	Name	Location	Method, References, Comments	Contained in reference set of [21]?	Used for statistics?
Phatr2a_30160	40S ribosomal protein S26	LD	Mass spectrometric peptide mapping of proteins from isolated lipid droplets [39], sequence identical to UniProt B7G9T0.	no	no
Phatr2a_30315	Predicted protein	LD	Mass spectrometric peptide mapping of proteins from isolated lipid droplets [39], sequence identical to UniProt B7GAG0.	no	no
Phatr2a_30334	Predicted protein	LD	Mass spectrometric peptide mapping of proteins from isolated lipid droplets [39], sequence identical to UniProt B7GAI2.	no	no
Phatr2a_30446	T-complex protein 1 subunit delta	LD	Mass spectrometric peptide mapping of proteins from isolated lipid droplets [39], sequence identical to UniProt B7GB02.	no	no
Phatr2a_30486	Predicted protein	LD	Mass spectrometric peptide mapping of proteins from isolated lipid droplets [39], sequence identical to UniProt B7GB86.	no	no
Phatr2a_30514	Ornithine transcarbamylase	LD	Mass spectrometric peptide mapping of proteins from isolated lipid droplets [39], sequence identical to UniProt B7GBF1.	no	no
Phatr2a_30578	Predicted protein	LD	Mass spectrometric peptide mapping of proteins from isolated lipid droplets [39], sequence identical to UniProt B7GBR2.	no	no
Phatr2a_30660	Predicted protein	LD	Mass spectrometric peptide mapping of proteins from isolated lipid droplets [39], sequence identical to UniProt B7GBL1.	no	no
Phatr2a_30770	Cytochrome b5	LD	Mass spectrometric peptide mapping of proteins from isolated lipid droplets [39], sequence identical to UniProt B7GCG7.	no	no
Phatr2a_31160	Predicted protein	LD	Mass spectrometric peptide mapping of proteins from isolated lipid droplets [39], sequence identical to UniProt B7GDZ3.	no	no
Phatr2a_31322	Calmodulin	LD	Mass spectrometric peptide mapping of proteins from isolated lipid droplets [39], sequence identical to UniProt B7GEH1.	no	no
Phatr2a_31619	Predicted protein	LD	Mass spectrometric peptide mapping of proteins from isolated lipid droplets [39], sequence identical to UniProt B7FNV6.	no	no
Phatr2a_31846	Predicted protein	LD	Mass spectrometric peptide mapping of proteins from isolated lipid droplets [39], sequence identical to UniProt B7FPI2.	no	no
Phatr2a_31882	6-phosphogluconolactonase	LD	Mass spectrometric peptide mapping of proteins from isolated lipid droplets [39], sequence identical to UniProt B7FPL4.	no	no
Phatr2a_31919	Predicted protein	LD	Mass spectrometric peptide mapping of proteins from isolated lipid droplets [39], sequence identical to UniProt B7FPQ0.	no	no
Phatr2a_32759	Predicted protein (Fragment)	LD	Mass spectrometric peptide mapping of proteins from isolated lipid droplets [39], sequence identical to UniProt B7FSJ6.	no	no
Phatr2a_32813	Predicted protein	LD	Mass spectrometric peptide mapping of proteins from isolated lipid droplets [39], sequence identical to UniProt B7FSP9.	no	no

Protein ID	Name	Location	Method, References, Comments	Contained in reference set of [21]?	Used for statistics?
Phatr2a_33069	Peptide-methionine (S)-S-oxide reductase (Fragment)	LD	Mass spectrometric peptide mapping of proteins from isolated lipid droplets [39], UniProt ID in publication is B7FTF5, has 81 residues of N-terminal extension (MNSQMCSSCKETLLEKTMNLPISTGTVGLG + 51 aa).	no	no
Phatr2a_33757	Predicted protein	LD	Mass spectrometric peptide mapping of proteins from isolated lipid droplets [39], sequence identical to UniProt B7FTS4.	no	no
Phatr2a_34007	Predicted protein	LD	Mass spectrometric peptide mapping of proteins from isolated lipid droplets [39], UniProt ID in publication is B7FUG7, has 67 residues of N-terminal extension (MAALAFARLKESCLREEPTVEDAVASRCPK + 37 aa).	no	no
Phatr2a_34028	Predicted protein	LD	Mass spectrometric peptide mapping of proteins from isolated lipid droplets [39], sequence identical to UniProt B7FUI8.	no	no
Phatr2a_34071	Predicted protein	LD	Mass spectrometric peptide mapping of proteins from isolated lipid droplets [39], sequence identical to UniProt B7FUM9.	no	no
Phatr2a_34120	Predicted protein	LD	Mass spectrometric peptide mapping of proteins from isolated lipid droplets [39], sequence identical to UniProt B7FUS9.	no	no
Phatr2a_34146	60S ribosomal protein L6	LD	Mass spectrometric peptide mapping of proteins from isolated lipid droplets [39], sequence identical to UniProt B7FUV3.	no	no
Phatr2a_35262	Predicted protein	LD	Mass spectrometric peptide mapping of proteins from isolated lipid droplets [39], sequence identical to UniProt B7FY09.	no	no
Phatr2a_35386	Predicted protein	LD	Mass spectrometric peptide mapping of proteins from isolated lipid droplets [39], sequence identical to UniProt B7FYD2.	no	no
Phatr2a_35566	Hypoxanthine phosphoribosyltransferase	LD	Mass spectrometric peptide mapping of proteins from isolated lipid droplets [39], sequence identical to UniProt B7FYM2.	no	no
Phatr2a_35590	Predicted protein	LD	Mass spectrometric peptide mapping of proteins from isolated lipid droplets [39], sequence identical to UniProt B7FYP7.	no	no
Phatr2a_35594	Predicted protein	LD	Mass spectrometric peptide mapping of proteins from isolated lipid droplets [39], sequence identical to UniProt B7FYQ1.	no	no
Phatr2a_35766	Predicted protein	LD	Mass spectrometric peptide mapping of proteins from isolated lipid droplets [39], sequence identical to UniProt B7FZ72.	no	no
Phatr2a_35939	Predicted protein	LD	Mass spectrometric peptide mapping of proteins from isolated lipid droplets [39], sequence identical to UniProt B7FZX8.	no	no
Phatr2a_36226	60S ribosomal protein L18a	LD	Mass spectrometric peptide mapping of proteins from isolated lipid droplets [39], sequence identical to UniProt B7G0R5.	no	no
Phatr2a_36600	Predicted protein	LD	Mass spectrometric peptide mapping of proteins from isolated lipid droplets [39], sequence identical to UniProt B7G1T6.	no	no

Protein ID	Name	Location	Method, References, Comments	Contained in reference set of [21]?	Used for statistics?
Phatr2a_36616	Peptide-methionine (S)-S-oxide reductase (Fragment)	LD	Mass spectrometric peptide mapping of proteins from isolated lipid droplets [39], UniProt ID in publication is B7G1V2, has 107 residues of N-terminal extension (MKIHRSATLLLASAASSAYAFSLTGGSRHG + 77 aa).	no	no
Phatr2a_36913	Predicted protein	LD	Mass spectrometric peptide mapping of proteins from isolated lipid droplets [39], sequence identical to UniProt B5Y408.	no	no
Phatr2a_37283	Predicted protein	LD	Mass spectrometric peptide mapping of proteins from isolated lipid droplets [39], sequence identical to UniProt B7G2N1.	no	no
Phatr2a_37468	Predicted protein	LD	Mass spectrometric peptide mapping of proteins from isolated lipid droplets [39], sequence identical to UniProt B7G3W5.	no	no
Phatr2a_38174	Predicted protein	LD	Mass spectrometric peptide mapping of proteins from isolated lipid droplets [39], sequence identical to UniProt B7G553.	no	no
Phatr2a_38192	Predicted protein	LD	Mass spectrometric peptide mapping of proteins from isolated lipid droplets [39], sequence identical to UniProt B7G570.	no	no
Phatr2a_38377	Predicted protein	LD	Mass spectrometric peptide mapping of proteins from isolated lipid droplets [39], UniProt ID in publication is B7G5Q3, has 71 residues of N-terminal extension (MTLSLRASFSLQRSQNRNTTLSLFLIRTLV + 41 aa).	no	no
Phatr2a_38724	Predicted protein	LD	Mass spectrometric peptide mapping of proteins from isolated lipid droplets [39], sequence identical to UniProt B7G6N7.	no	no
Phatr2a_39000	Predicted protein	LD	Mass spectrometric peptide mapping of proteins from isolated lipid droplets [39], UniProt ID in publication is B7G7J1, has 380 residues of N-terminal extension (MIPSSAVLISDNVSIQCKVRRSPCSTNPKF + 350 aa).	no	no
Phatr2a_39237	Predicted protein	LD	Mass spectrometric peptide mapping of proteins from isolated lipid droplets [39], sequence identical to UniProt B7G7G1.	no	no
Phatr2a_39526	Eukaryotic translation initiation factor 3 subunit I	LD	Mass spectrometric peptide mapping of proteins from isolated lipid droplets [39], sequence identical to UniProt B7G8W6.	no	no
Phatr2a_39549	Predicted protein (Fragment)	LD	Mass spectrometric peptide mapping of proteins from isolated lipid droplets [39], UniProt ID in publication is B7G8Y9, has 46 residues of N-terminal extension (MADKTTKLVAADPASGVKVNAGIIAQPFRD + 16 aa), and 80 positions of C-terminal extension (50 aa + AFGRSLIRNGGNCAVSLSSRRETMVTFVA*).	no	no
Phatr2a_39681	3-hydroxyacyl-coenzyme A dehydrogenase	LD	Mass spectrometric peptide mapping of proteins from isolated lipid droplets [39], sequence identical to UniProt B7G9C0.	no	no
Phatr2a_39687	Predicted protein	LD	Mass spectrometric peptide mapping of proteins from isolated lipid droplets [39], sequence identical to UniProt B7G9C7.	no	no

Protein ID	Name	Location	Method, References, Comments	Contained in reference set of [21]?	Used for statistics?
Phatr2a_39942	Glucose-6-phosphate 1-epimerase	LD	Mass spectrometric peptide mapping of proteins from isolated lipid droplets [39], sequence identical to UniProt B7GA17.	no	no
Phatr2a_40305	Predicted protein	LD	Mass spectrometric peptide mapping of proteins from isolated lipid droplets [39], UniProt ID in publication is B7GB24, has 248 residues of N-terminal extension (MRRDCGFAIISLSSVLQHECCSQKSRIESG + 218 aa).	no	no
Phatr2a_41601	Predicted protein	LD	Mass spectrometric peptide mapping of proteins from isolated lipid droplets [39], sequence identical to UniProt B7GEN1.	no	no
Phatr2a_41623	Peptidylprolyl isomerase (Fragment)	LD	Mass spectrometric peptide mapping of proteins from isolated lipid droplets [39], UniProt ID in publication is B7FXI1, has 24 residues of N-terminal extension (MFLSRSFFSFVGVVAVASLSPYAA), and 85 positions of C-terminal extension (55 aa + LKLWMERRIHLLQQMVGPPATSDDAENEL*).	no	no
Phatr2a_41812	Succinate dehydrogenase [ubiquinone] flavoprotein subunit, mitochondrial	LD	Mass spectrometric peptide mapping of proteins from isolated lipid droplets [39], sequence identical to UniProt B5Y5N6.	no	no
Phatr2a_42426	Predicted protein	LD	Mass spectrometric peptide mapping of proteins from isolated lipid droplets [39], sequence identical to UniProt B7FR90.	no	no
Phatr2a_42434	Predicted protein	LD	Mass spectrometric peptide mapping of proteins from isolated lipid droplets [39], sequence identical to UniProt B7FR99.	no	no
Phatr2a_42458	Phosphoserine transaminase	LD	Mass spectrometric peptide mapping of proteins from isolated lipid droplets [39], sequence identical to UniProt B7FRD6.	no	no
Phatr2a_42481	Predicted protein	LD	Mass spectrometric peptide mapping of proteins from isolated lipid droplets [39], sequence identical to UniProt B7FRF9.	no	no
Phatr2a_42500	Predicted protein	LD	Mass spectrometric peptide mapping of proteins from isolated lipid droplets [39], sequence identical to UniProt B7FRI0.	no	no
Phatr2a_42504	Predicted protein	LD	Mass spectrometric peptide mapping of proteins from isolated lipid droplets [39], sequence identical to UniProt B7FRI5.	no	no
Phatr2a_42566	Predicted protein	LD	Mass spectrometric peptide mapping of proteins from isolated lipid droplets [39], sequence identical to UniProt B7FNS4.	no	no
Phatr2a_42651	Peptide-methionine (S)-S-oxide reductase	LD	Mass spectrometric peptide mapping of proteins from isolated lipid droplets [39], UniProt ID in publication is B7FP23, has 41 residues of N-terminal extension (MKGSVCLASLWHLVRVSLLFRDTNGPKLGF + 11 aa).	no	no
Phatr2a_42659	Predicted protein	LD	Mass spectrometric peptide mapping of proteins from isolated lipid droplets [39], sequence identical to UniProt B7FP35.	no	no
Phatr2a_42712	Predicted protein	LD	Mass spectrometric peptide mapping of proteins from isolated lipid droplets [39], sequence identical to UniProt B7FP98.	no	no

Protein ID	Name	Location	Method, References, Comments	Contained in reference set of [21]?	Used for statistics?
Phatr2a_42716	Elongation factor Ts, mitochondrial	LD	Mass spectrometric peptide mapping of proteins from isolated lipid droplets [39], UniProt ID in publication is B7FPA4, has 44 residues of N-terminal extension (MRLLCRSFVQCSARLSLRGRVANLSQNSDF + 14 aa).	no	no
Phatr2a_42723	Predicted protein	LD	Mass spectrometric peptide mapping of proteins from isolated lipid droplets [39], sequence identical to UniProt B7FPB2.	no	no
Phatr2a_42730	Predicted protein	LD	Mass spectrometric peptide mapping of proteins from isolated lipid droplets [39], sequence identical to UniProt B7FPC1.	no	no
Phatr2a_42736	Predicted protein	LD	Mass spectrometric peptide mapping of proteins from isolated lipid droplets [39], sequence identical to UniProt B7FPC8.	no	no
Phatr2a_42760	Oxidoreductase	LD	Mass spectrometric peptide mapping of proteins from isolated lipid droplets [39], UniProt ID in publication is B7FPF9, has 53 residues of N-terminal extension (MRSYDGSRLAVLVAVLCRMPTLSLAFTVPL + 23 aa).	no	no
Phatr2a_42829	Regulatory proteasome non-atpase subunit 3 (Fragment)	LD	Mass spectrometric peptide mapping of proteins from isolated lipid droplets [39], UniProt ID in publication is B7FPP8, has 318 residues of N-terminal extension (MSMNSARNPPERISIRQALDILSSRSSENH + 288 aa).	no	no
Phatr2a_42832	Superoxide dismutase	LD	Mass spectrometric peptide mapping of proteins from isolated lipid droplets [39], sequence identical to UniProt B7FPQ3.	no	no
Phatr2a_42858	Predicted protein	LD	Mass spectrometric peptide mapping of proteins from isolated lipid droplets [39], sequence identical to UniProt B7FPT6.	no	no
Phatr2a_42949	Predicted protein	LD	Mass spectrometric peptide mapping of proteins from isolated lipid droplets [39], sequence identical to UniProt B7FQ44.	no	no
Phatr2a_42962	2-phosphoglycerate dehydratase (Fragment)	LD	Mass spectrometric peptide mapping of proteins from isolated lipid droplets [39], UniProt ID in publication is B7FQ57, has 4 residues of N-terminal extension (MTKP), and 9 positions of C-terminal extension (IALEAISD*).	no	no
Phatr2a_42987	Predicted protein (Fragment)	LD	Mass spectrometric peptide mapping of proteins from isolated lipid droplets [39], UniProt ID in publication is B7FQ87, has 1263 residues of N-terminal extension (MGCAGSKEAAVDVGEPTVPAPEAKVQKELE + 1233 aa), and 466 positions of C-terminal extension (436 aa + FDSYRHLENEAPSNKPLLSPAQEQQFKDF*).	no	no
Phatr2a_43003	Regulatory proteasome non-atpase subunit 5	LD	Mass spectrometric peptide mapping of proteins from isolated lipid droplets [39], UniProt ID in publication is B7FQA9, has 68 residues of N-terminal extension (MSHDFEKYVIESYKTVVDEGERSGSKDRPN + 38 aa).	no	no
Phatr2a_43008	Component of oligomeric Golgi complex 8	LD	Mass spectrometric peptide mapping of proteins from isolated lipid droplets [39], sequence identical to UniProt B7FQB5.	no	no
Phatr2a_43038	Predicted protein	LD	Mass spectrometric peptide mapping of proteins from isolated lipid droplets [39], sequence identical to UniProt B7FQE8.	no	no

Protein ID	Name	Location	Method, References, Comments	Contained in reference set of [21]?	Used for statistics?
Phatr2a_43041	Predicted protein	LD	Mass spectrometric peptide mapping of proteins from isolated lipid droplets [39], sequence identical to UniProt B7FQF2.	no	no
Phatr2a_43116	Predicted protein	LD	Mass spectrometric peptide mapping of proteins from isolated lipid droplets [39], sequence identical to UniProt B7FQP0.	no	no
Phatr2a_43127	Predicted protein	LD	Mass spectrometric peptide mapping of proteins from isolated lipid droplets [39], sequence identical to UniProt B7FQQ6.	no	no
Phatr2a_43216	Predicted protein	LD	Mass spectrometric peptide mapping of proteins from isolated lipid droplets [39], UniProt ID in publication is B7FR08, has 353 residues of N-terminal extension (MTMANESLTSKPIAAPFEWLAGGLFFGKSK + 323 aa).	no	no
Phatr2a_43225	Predicted protein	LD	Mass spectrometric peptide mapping of proteins from isolated lipid droplets [39], sequence identical to UniProt B7FR19.	no	no
Phatr2a_43249	Predicted protein	LD	Mass spectrometric peptide mapping of proteins from isolated lipid droplets [39], sequence identical to UniProt B7FR48.	no	no
Phatr2a_43251	Predicted protein	LD	Mass spectrometric peptide mapping of proteins from isolated lipid droplets [39], sequence identical to UniProt B7FR50.	no	no
Phatr2a_43302	Predicted protein	LD	Mass spectrometric peptide mapping of proteins from isolated lipid droplets [39], sequence identical to UniProt B7FRT1.	no	no
Phatr2a_43311	Predicted protein	LD	Mass spectrometric peptide mapping of proteins from isolated lipid droplets [39], sequence identical to UniProt B7FRU2.	no	no
Phatr2a_43348	Predicted protein	LD	Mass spectrometric peptide mapping of proteins from isolated lipid droplets [39], sequence identical to UniProt B7FRY4.	no	no
Phatr2a_43352	Predicted protein	LD	Mass spectrometric peptide mapping of proteins from isolated lipid droplets [39], sequence identical to UniProt B7FRY8.	no	no
Phatr2a_43427	Predicted protein	LD	Mass spectrometric peptide mapping of proteins from isolated lipid droplets [39], sequence identical to UniProt B7FS74.	no	no
Phatr2a_43489	Predicted protein	LD	Mass spectrometric peptide mapping of proteins from isolated lipid droplets [39], sequence identical to UniProt B7FSE1.	no	no
Phatr2a_43548	Predicted protein (Fragment)	LD	Mass spectrometric peptide mapping of proteins from isolated lipid droplets [39], UniProt ID in publication is B7FSL6, has 121 positions of C-terminal extension (91 aa + EELDEFGKQTGGEHYSAMSGTEATTVDAPI).	no	no
Phatr2a_43658	Predicted protein	LD	Mass spectrometric peptide mapping of proteins from isolated lipid droplets [39], sequence identical to UniProt B7FT11.	no	no
Phatr2a_43671	Predicted protein	LD	Mass spectrometric peptide mapping of proteins from isolated lipid droplets [39], sequence identical to UniProt B7FT25.	no	no

Protein ID	Name	Location	Method, References, Comments	Contained in reference set of [21]?	Used for statistics?
Phatr2a_43709	Predicted protein	LD	Mass spectrometric peptide mapping of proteins from isolated lipid droplets [39], sequence identical to UniProt B7FT71.	no	no
Phatr2a_43770	Predicted protein	LD	Mass spectrometric peptide mapping of proteins from isolated lipid droplets [39], sequence identical to UniProt B7FTE7.	no	no
Phatr2a_43801	Predicted protein	LD	Mass spectrometric peptide mapping of proteins from isolated lipid droplets [39], sequence identical to UniProt B5Y4C7.	no	no
Phatr2a_43840	Predicted protein	LD	Mass spectrometric peptide mapping of proteins from isolated lipid droplets [39], sequence identical to UniProt B5Y4I0.	no	no
Phatr2a_43841	Predicted protein	LD	Mass spectrometric peptide mapping of proteins from isolated lipid droplets [39], sequence identical to UniProt B5Y4I1.	no	no
Phatr2a_43922	Predicted protein	LD	Mass spectrometric peptide mapping of proteins from isolated lipid droplets [39], sequence identical to UniProt B5Y4S4.	no	no
Phatr2a_43944	NADH dehydrogenase [ubiquinone] flavoprotein 1, mitochondrial	LD	Mass spectrometric peptide mapping of proteins from isolated lipid droplets [39], sequence identical to UniProt B5Y4V1.	no	no
Phatr2a_44005	Predicted protein	LD	Mass spectrometric peptide mapping of proteins from isolated lipid droplets [39], sequence identical to UniProt B5Y519.	no	no
Phatr2a_44109	Annexin	LD	Mass spectrometric peptide mapping of proteins from isolated lipid droplets [39], sequence identical to UniProt B5Y5D4.	no	no
Phatr2a_44327	Predicted protein	LD	Mass spectrometric peptide mapping of proteins from isolated lipid droplets [39], sequence identical to UniProt B7FTQ7.	no	no
Phatr2a_44343	Predicted protein	LD	Mass spectrometric peptide mapping of proteins from isolated lipid droplets [39], sequence identical to UniProt B7FTS5.	no	no
Phatr2a_44357	Predicted protein	LD	Mass spectrometric peptide mapping of proteins from isolated lipid droplets [39], sequence identical to UniProt B7FTU6.	no	no
Phatr2a_44364	Predicted protein	LD	Mass spectrometric peptide mapping of proteins from isolated lipid droplets [39], sequence identical to UniProt B7FTV4.	no	no
Phatr2a_44393	Predicted protein	LD	Mass spectrometric peptide mapping of proteins from isolated lipid droplets [39], sequence identical to UniProt B7FTY3.	no	no
Phatr2a_44436	Methyltransferase (Fragment)	LD	Mass spectrometric peptide mapping of proteins from isolated lipid droplets [39], UniProt ID in publication is B7FU36, has 223 residues of N-terminal extension (MVSRSTTTQNFPNFWVDSDFFRKGQFSPQT + 193 aa).	no	no
Phatr2a_44479	Predicted protein	LD	Mass spectrometric peptide mapping of proteins from isolated lipid droplets [39], sequence identical to UniProt B7FU94.	no	no

Protein ID	Name	Location	Method, References, Comments	Contained in reference set of [21]?	Used for statistics?
Phatr2a_44509	Predicted protein	LD	Mass spectrometric peptide mapping of proteins from isolated lipid droplets [39], UniProt ID in publication is B7FUC8, has 100 residues of N-terminal extension (MLRGGSASRLLPRNVIKVAPFWNVRTAFST + 70 aa).	no	no
Phatr2a_44510	Predicted protein	LD	Mass spectrometric peptide mapping of proteins from isolated lipid droplets [39], sequence identical to UniProt B7FUC9.	no	no
Phatr2a_44546	Predicted protein	LD	Mass spectrometric peptide mapping of proteins from isolated lipid droplets [39], sequence identical to UniProt B7FUG6.	no	no
Phatr2a_44596	Predicted protein	LD	Mass spectrometric peptide mapping of proteins from isolated lipid droplets [39], sequence identical to UniProt B7FUM1.	no	no
Phatr2a_44603	Predicted protein	LD	Mass spectrometric peptide mapping of proteins from isolated lipid droplets [39], sequence identical to UniProt B7FUM8.	no	no
Phatr2a_44630	Predicted protein	LD	Mass spectrometric peptide mapping of proteins from isolated lipid droplets [39], sequence identical to UniProt B7FUR1.	no	no
Phatr2a_44639	Predicted protein	LD	Mass spectrometric peptide mapping of proteins from isolated lipid droplets [39], sequence identical to UniProt B7FUS2.	no	no
Phatr2a_44676	Predicted protein	LD	Mass spectrometric peptide mapping of proteins from isolated lipid droplets [39], UniProt ID in publication is B7FUW5, has 36 residues of N-terminal extension (MSAPPDLHPNLVVSPSKAHAALFTKLREFV + 6 aa).	no	no
Phatr2a_44715	Conserved oligomeric Golgi complex subunit 6	LD	Mass spectrometric peptide mapping of proteins from isolated lipid droplets [39], sequence identical to UniProt B7FVD5.	no	no
Phatr2a_44724	Predicted protein	LD	Mass spectrometric peptide mapping of proteins from isolated lipid droplets [39], sequence identical to UniProt B7FVE9.	no	no
Phatr2a_44796	Predicted protein	LD	Mass spectrometric peptide mapping of proteins from isolated lipid droplets [39], sequence identical to UniProt B7FVP0.	no	no
Phatr2a_44822	Predicted protein	LD	Mass spectrometric peptide mapping of proteins from isolated lipid droplets [39], sequence identical to UniProt B7FVS1.	no	no
Phatr2a_44851	Predicted protein	LD	Mass spectrometric peptide mapping of proteins from isolated lipid droplets [39], sequence identical to UniProt B7FVW2.	no	no
Phatr2a_44861	NADH dehydrogenase [ubiquinone] 1 alpha subcomplex subunit 12	LD	Mass spectrometric peptide mapping of proteins from isolated lipid droplets [39], UniProt ID in publication is B7FVX2, has 25 residues of N-terminal extension (MVWQAANNLRQALKYRGGWKGLLEH).	no	no
Phatr2a_44902	Predicted protein	LD	Mass spectrometric peptide mapping of proteins from isolated lipid droplets [39], sequence identical to UniProt B7FW24.	no	no

Protein ID	Name	Location	Method, References, Comments	Contained in reference set of [21]?	Used for statistics?
Phatr2a_45005	NADH dehydrogenase [ubiquinone] 1 beta subcomplex subunit 9 (Fragment)	LD	Mass spectrometric peptide mapping of proteins from isolated lipid droplets [39], UniProt ID in publication is B7FV90, has 61 residues of N-terminal extension (MTLVRPQPLNDIFLAAANQTRQKARSLTHN + 31 aa), and 49 positions of C-terminal extension (19 aa + NPDMSLCTPETGRNAVGTVLVDFYKKNME*).	no	no
Phatr2a_45017	Predicted protein	LD	Mass spectrometric peptide mapping of proteins from isolated lipid droplets [39], sequence identical to UniProt B7FVA8.	no	no
Phatr2a_45049	Predicted protein (Fragment)	LD	Mass spectrometric peptide mapping of proteins from isolated lipid droplets [39], UniProt ID in publication is B7FWA9, has 423 residues of N-terminal extension (MMDGLPARQSSLPLLMSPHASSSPPRNSVS + 393 aa).	no	no
Phatr2a_45053	Predicted protein	LD	Mass spectrometric peptide mapping of proteins from isolated lipid droplets [39], sequence identical to UniProt B7FWB4.	no	no
Phatr2a_45105	Predicted protein	LD	Mass spectrometric peptide mapping of proteins from isolated lipid droplets [39], UniProt ID in publication is B7FWH1, has 5 residues of N-terminal extension (MQYRK).	no	no
Phatr2a_45122	Predicted protein	LD	Mass spectrometric peptide mapping of proteins from isolated lipid droplets [39], sequence identical to UniProt B7FWJ1.	no	no
Phatr2a_45205	Predicted protein	LD	Mass spectrometric peptide mapping of proteins from isolated lipid droplets [39], sequence identical to UniProt B7FWU2.	no	no
Phatr2a_45226	Predicted protein	LD	Mass spectrometric peptide mapping of proteins from isolated lipid droplets [39], sequence identical to UniProt B7FWW9.	no	no
Phatr2a_45340	Predicted protein	LD	Mass spectrometric peptide mapping of proteins from isolated lipid droplets [39], sequence identical to UniProt B7FXC5.	no	no
Phatr2a_45351	Predicted protein (Fragment)	LD	Mass spectrometric peptide mapping of proteins from isolated lipid droplets [39], UniProt ID in publication is B7FXD7, has 80 residues of N-terminal extension (MAILSRTLVGVLTATFACIPHETNAFLKPH + 50 aa), and 28 positions of C-terminal extension (NDGPMGGLVKLDEAGDLDGMLKAAGSI*).	no	no
Phatr2a_45401	Predicted protein	LD	Mass spectrometric peptide mapping of proteins from isolated lipid droplets [39], sequence identical to UniProt B7FXK1.	no	no
Phatr2a_45408	Predicted protein	LD	Mass spectrometric peptide mapping of proteins from isolated lipid droplets [39], sequence identical to UniProt B7FXL0.	no	no
Phatr2a_45422	Predicted protein (Fragment)	LD	Mass spectrometric peptide mapping of proteins from isolated lipid droplets [39], UniProt ID in publication is B7FXM5, has 71 residues of N-terminal extension (MPGNNGRNVGNCVSPWSRMGSRPIWLILTC + 41 aa).	no	no
Phatr2a_45434	Nad-dependent epimerase/dehydratase	LD	Mass spectrometric peptide mapping of proteins from isolated lipid droplets [39], sequence identical to UniProt B7FXN9.	no	no

Protein ID	Name	Location	Method, References, Comments	Contained in reference set of [21]?	Used for statistics?
Phatr2a_45437	Predicted protein	LD	Mass spectrometric peptide mapping of proteins from isolated lipid droplets [39], UniProt ID in publication is B7FXP2, has 147 residues of N-terminal extension (MVRGENEVTARARQNTPERLYRERHPKQKP + 117 aa).	no	no
Phatr2a_45447	ATP-sulfurylase	LD	Mass spectrometric peptide mapping of proteins from isolated lipid droplets [39], UniProt ID in publication is B7FXQ3, has 167 residues of N-terminal extension (MAKYAYAPLSIDDSGTSRRNNNNNTQSGYD + 137 aa).	no	no
Phatr2a_45510	Long chain acyl-coa synthetase	LD	Mass spectrometric peptide mapping of proteins from isolated lipid droplets [39], sequence identical to UniProt B7FXX6.	no	no
Phatr2a_45582	Predicted protein	LD	Mass spectrometric peptide mapping of proteins from isolated lipid droplets [39], sequence identical to UniProt B7FY59.	no	no
Phatr2a_45592	Phosphoglycerate dehydrogenase	LD	Mass spectrometric peptide mapping of proteins from isolated lipid droplets [39], sequence identical to UniProt B7FY72.	no	no
Phatr2a_45605	Predicted protein	LD	Mass spectrometric peptide mapping of proteins from isolated lipid droplets [39], sequence identical to UniProt B7FY87.	no	no
Phatr2a_45653	Predicted protein	LD	Mass spectrometric peptide mapping of proteins from isolated lipid droplets [39], sequence identical to UniProt B7FYE8.	no	no
Phatr2a_45792	Predicted protein	LD	Mass spectrometric peptide mapping of proteins from isolated lipid droplets [39], sequence identical to UniProt B7FYP3.	no	no
Phatr2a_45811	Predicted protein	LD	Mass spectrometric peptide mapping of proteins from isolated lipid droplets [39], sequence identical to UniProt B7FYR7.	no	no
Phatr2a_45866	Peptidylprolyl isomerase (Fragment)	LD	Mass spectrometric peptide mapping of proteins from isolated lipid droplets [39], UniProt ID in publication is B7FYY9, has 300 residues of N-terminal extension (MLEYVASDSPESRMSVRSTIWHLPSPHTPQ + 270 aa).	no	no
Phatr2a_45894	Predicted protein	LD	Mass spectrometric peptide mapping of proteins from isolated lipid droplets [39], sequence identical to UniProt B7FZ26.	no	no
Phatr2a_45918	Predicted protein	LD	Mass spectrometric peptide mapping of proteins from isolated lipid droplets [39], sequence identical to UniProt B7FZ57.	no	no
Phatr2a_45921	Predicted protein	LD	Mass spectrometric peptide mapping of proteins from isolated lipid droplets [39], sequence identical to UniProt B7FZ61.	no	no
Phatr2a_45947	Predicted protein	LD	Mass spectrometric peptide mapping of proteins from isolated lipid droplets [39], sequence identical to UniProt B7FZ92.	no	no
Phatr2a_45948	Predicted protein (Fragment)	LD	Mass spectrometric peptide mapping of proteins from isolated lipid droplets [39], UniProt ID in publication is B7FZ93, has 165 residues of N-terminal extension (MTSRISIGLFIHEEGNRAHGNIASLSSRVW + 135 aa).	no	no

Protein ID	Name	Location	Method, References, Comments	Contained in reference set of [21]?	Used for statistics?
Phatr2a_45983	Pyruvate dehydrogenase (acetyl-transferring)	LD	Mass spectrometric peptide mapping of proteins from isolated lipid droplets [39], UniProt ID in publication is B7FZE1, has 815 residues of N-terminal extension (MKFSTATLALCVATASAFVPVALRPQSNGV + 785 aa).	no	no
Phatr2a_46006	Predicted protein	LD	Mass spectrometric peptide mapping of proteins from isolated lipid droplets [39], sequence identical to UniProt B7FZH4.	no	no
Phatr2a_46077	Predicted protein	LD	Mass spectrometric peptide mapping of proteins from isolated lipid droplets [39], sequence identical to UniProt B7FZZ2.	no	no
Phatr2a_46098	Predicted protein	LD	Mass spectrometric peptide mapping of proteins from isolated lipid droplets [39], sequence identical to UniProt B7G022.	no	no
Phatr2a_46100	Predicted protein	LD	Mass spectrometric peptide mapping of proteins from isolated lipid droplets [39], sequence identical to UniProt B7G025.	no	no
Phatr2a_46131	Predicted protein	LD	Mass spectrometric peptide mapping of proteins from isolated lipid droplets [39], sequence identical to UniProt B7G059.	no	no
Phatr2a_46187	Predicted protein	LD	Mass spectrometric peptide mapping of proteins from isolated lipid droplets [39], sequence identical to UniProt B7G0C7.	no	no
Phatr2a_46215	Predicted protein	LD	Mass spectrometric peptide mapping of proteins from isolated lipid droplets [39], sequence identical to UniProt B7G0H1.	no	no
Phatr2a_46248	Predicted protein	LD	Mass spectrometric peptide mapping of proteins from isolated lipid droplets [39], sequence identical to UniProt B7G0K8.	no	no
Phatr2a_46320	Eukaryotic translation initiation factor 3 subunit A	LD	Mass spectrometric peptide mapping of proteins from isolated lipid droplets [39], sequence identical to UniProt B7G0T8.	no	no
Phatr2a_46321	PRA1 family protein	LD	Mass spectrometric peptide mapping of proteins from isolated lipid droplets [39], sequence identical to UniProt B7G0T9.	no	no
Phatr2a_46388	Predicted protein (Fragment)	LD	Mass spectrometric peptide mapping of proteins from isolated lipid droplets [39], UniProt ID in publication is B7G119, has 71 residues of N-terminal extension (MYDFIKQLLLGNPQSQQRIELQHSSFRNNS + 41 aa), and 44 positions of C-terminal extension (14 aa + TEATEHGDLAHLLMFSMNEHPGSGRSDIS*).	no	no
Phatr2a_46461	Homoserine kinase	LD	Mass spectrometric peptide mapping of proteins from isolated lipid droplets [39], sequence identical to UniProt B7G1B0.	no	no
Phatr2a_46484	Predicted protein	LD	Mass spectrometric peptide mapping of proteins from isolated lipid droplets [39], sequence identical to UniProt B7G1E0.	no	no
Phatr2a_46524	Predicted protein	LD	Mass spectrometric peptide mapping of proteins from isolated lipid droplets [39], sequence identical to UniProt B7G1I4.	no	no

Protein ID	Name	Location	Method, References, Comments	Contained in reference set of [21]?	Used for statistics?
Phatr2a_46548	Predicted protein	LD	Mass spectrometric peptide mapping of proteins from isolated lipid droplets [39], sequence identical to UniProt B7G1L4.	no	no
Phatr2a_46579	Predicted protein	LD	Mass spectrometric peptide mapping of proteins from isolated lipid droplets [39], sequence identical to UniProt B7G1Q3.	no	no
Phatr2a_46588	Predicted protein	LD	Mass spectrometric peptide mapping of proteins from isolated lipid droplets [39], sequence identical to UniProt B7G1R9.	no	no
Phatr2a_46599	Clathrin light chain	LD	Mass spectrometric peptide mapping of proteins from isolated lipid droplets [39], sequence identical to UniProt B7G1T5.	no	no
Phatr2a_46612	Predicted protein	LD	Mass spectrometric peptide mapping of proteins from isolated lipid droplets [39], sequence identical to UniProt B7G1V3.	no	no
Phatr2a_46623	Predicted protein	LD	Mass spectrometric peptide mapping of proteins from isolated lipid droplets [39], sequence identical to UniProt B7G1W7.	no	no
Phatr2a_46658	Predicted protein	LD	Mass spectrometric peptide mapping of proteins from isolated lipid droplets [39], UniProt ID in publication is B5Y3C9, has 113 residues of N-terminal extension (MRLTLSTQAALEGWHTYVVRNSSFPGRLPV + 83 aa).	no	no
Phatr2a_46664	Predicted protein	LD	Mass spectrometric peptide mapping of proteins from isolated lipid droplets [39], sequence identical to UniProt B5Y3D6.	no	no
Phatr2a_46707	Predicted protein	LD	Mass spectrometric peptide mapping of proteins from isolated lipid droplets [39], sequence identical to UniProt B5Y3J1.	no	no
Phatr2a_46721	Predicted protein	LD	Mass spectrometric peptide mapping of proteins from isolated lipid droplets [39], sequence identical to UniProt B5Y3K6.	no	no
Phatr2a_46758	Predicted protein	LD	Mass spectrometric peptide mapping of proteins from isolated lipid droplets [39], sequence identical to UniProt B5Y3P4.	no	no
Phatr2a_46820	Chloride channel protein	LD	Mass spectrometric peptide mapping of proteins from isolated lipid droplets [39], UniProt ID in publication is B5Y3W1, has 84 residues of N-terminal extension (MKMTSRGLSPIKPSWILRDLAGKNAQEFRG + 54 aa).	no	no
Phatr2a_46861	Predicted protein	LD	Mass spectrometric peptide mapping of proteins from isolated lipid droplets [39], UniProt ID in publication is B5Y407, has 56 residues of N-terminal extension (MAS-RDLTGAFMERRSAFLRKRSANHDSNER + 26 aa).	no	no
Phatr2a_46917	Predicted protein	LD	Mass spectrometric peptide mapping of proteins from isolated lipid droplets [39], sequence identical to UniProt B5Y470.	no	no
Phatr2a_46924	Predicted protein	LD	Mass spectrometric peptide mapping of proteins from isolated lipid droplets [39], sequence identical to UniProt B5Y477.	no	no
Phatr2a_46937	Predicted protein	LD	Mass spectrometric peptide mapping of proteins from isolated lipid droplets [39], sequence identical to UniProt B5Y495.	no	no

Protein ID	Name	Location	Method, References, Comments	Contained in reference set of [21]?	Used for statistics?
Phatr2a_47022	Predicted protein	LD	Mass spectrometric peptide mapping of proteins from isolated lipid droplets [39], sequence identical to UniProt B7G268.	no	no
Phatr2a_47027	Eukaryotic translation initiation factor 3 subunit M	LD	Mass spectrometric peptide mapping of proteins from isolated lipid droplets [39], sequence identical to UniProt B7G274.	no	no
Phatr2a_47031	T-complex protein 1 subunit eta	LD	Mass spectrometric peptide mapping of proteins from isolated lipid droplets [39], UniProt ID in publication is B7G279, has 5 residues of N-terminal extension (MNAQG).	no	no
Phatr2a_47067	5'-phosphoribosylglycinamide transformylase	LD	Mass spectrometric peptide mapping of proteins from isolated lipid droplets [39], sequence identical to UniProt B7G2B9.	no	no
Phatr2a_47099	Translocation protein SEC62	LD	Mass spectrometric peptide mapping of proteins from isolated lipid droplets [39], sequence identical to UniProt B7G2F8.	no	no
Phatr2a_47152	Predicted protein	LD	Mass spectrometric peptide mapping of proteins from isolated lipid droplets [39], sequence identical to UniProt B7G2N7.	no	no
Phatr2a_47178	Predicted protein	LD	Mass spectrometric peptide mapping of proteins from isolated lipid droplets [39], sequence identical to UniProt B7G2R8.	no	no
Phatr2a_47182	Predicted protein	LD	Mass spectrometric peptide mapping of proteins from isolated lipid droplets [39], sequence identical to UniProt B7G2S2.	no	no
Phatr2a_47197	Predicted protein (Fragment)	LD	Mass spectrometric peptide mapping of proteins from isolated lipid droplets [39], UniProt ID in publication is B7G2U0, has 18 residues of N-terminal extension (MIPYIRCYKYTSHSVYST), and 49 positions of C-terminal extension (19 aa + SLGKSWMWAVGGAAVTVGMGITIARHRRR*).	no	no
Phatr2a_47209	Predicted protein	LD	Mass spectrometric peptide mapping of proteins from isolated lipid droplets [39], sequence identical to UniProt B7G2V3.	no	no
Phatr2a_47271	Predicted protein	LD	Mass spectrometric peptide mapping of proteins from isolated lipid droplets [39], sequence identical to UniProt B7G3T2.	no	no
Phatr2a_47327	Exocyst subunit Exo70 family protein	LD	Mass spectrometric peptide mapping of proteins from isolated lipid droplets [39], sequence identical to UniProt B7G2Z1.	no	no
Phatr2a_47383	Predicted protein	LD	Mass spectrometric peptide mapping of proteins from isolated lipid droplets [39], sequence identical to UniProt B7G369.	no	no
Phatr2a_47403	Predicted protein	LD	Mass spectrometric peptide mapping of proteins from isolated lipid droplets [39], sequence identical to UniProt B7G395.	no	no
Phatr2a_47565	Predicted protein	LD	Mass spectrometric peptide mapping of proteins from isolated lipid droplets [39], sequence identical to UniProt B7G440.	no	no

Protein ID	Name	Location	Method, References, Comments	Contained in reference set of [21]?	Used for statistics?
Phatr2a_47590	Predicted protein	LD	Mass spectrometric peptide mapping of proteins from isolated lipid droplets [39], sequence identical to UniProt B7G469.	no	no
Phatr2a_47607	Predicted protein	LD	Mass spectrometric peptide mapping of proteins from isolated lipid droplets [39], sequence identical to UniProt B7G494.	no	no
Phatr2a_47683	Predicted protein	LD	Mass spectrometric peptide mapping of proteins from isolated lipid droplets [39], UniProt ID in publication is B7G4J1, has 12 residues of N-terminal extension (MLVRNAALLVIA).	no	no
Phatr2a_47742	Predicted protein	LD	Mass spectrometric peptide mapping of proteins from isolated lipid droplets [39], sequence identical to UniProt B7G4R1.	no	no
Phatr2a_47743	V-type proton ATPase proteolipid subunit	LD	Mass spectrometric peptide mapping of proteins from isolated lipid droplets [39], UniProt ID in publication is B7G4Q4, has 54 residues of N-terminal extension (MLQSVSKTLDLDGASLYEQQSGASSVLSAS + 24 aa).	no	no
Phatr2a_47804	Predicted protein	LD	Mass spectrometric peptide mapping of proteins from isolated lipid droplets [39], sequence identical to UniProt B7G4Z0.	no	no
Phatr2a_47843	Predicted protein	LD	Mass spectrometric peptide mapping of proteins from isolated lipid droplets [39], sequence identical to UniProt B7G539.	no	no
Phatr2a_47954	Predicted protein	LD	Mass spectrometric peptide mapping of proteins from isolated lipid droplets [39], sequence identical to UniProt B7G5H6.	no	no
Phatr2a_48084	Predicted protein	LD	Mass spectrometric peptide mapping of proteins from isolated lipid droplets [39], sequence identical to UniProt B7G5X7.	no	no
Phatr2a_48103	Predicted protein	LD	Mass spectrometric peptide mapping of proteins from isolated lipid droplets [39], sequence identical to UniProt B7G602.	no	no
Phatr2a_48211	Predicted protein	LD	Mass spectrometric peptide mapping of proteins from isolated lipid droplets [39], sequence identical to UniProt B7G6C7.	no	no
Phatr2a_48238	Predicted protein	LD	Mass spectrometric peptide mapping of proteins from isolated lipid droplets [39], sequence identical to UniProt B7G6G2.	no	no
Phatr2a_48245	Predicted protein	LD	Mass spectrometric peptide mapping of proteins from isolated lipid droplets [39], sequence identical to UniProt B7G6H1.	no	no
Phatr2a_48367	Predicted protein (Fragment)	LD	Mass spectrometric peptide mapping of proteins from isolated lipid droplets [39], UniProt ID in publication is B7G6W4, has 149 residues of N-terminal extension (MWYGLRPRDCRQGLLLFQVAVLFVRNCETR + 119 aa).	no	no
Phatr2a_48445	Predicted protein	LD	Mass spectrometric peptide mapping of proteins from isolated lipid droplets [39], sequence identical to UniProt B7G757.	no	no
Phatr2a_48449	Predicted protein	LD	Mass spectrometric peptide mapping of proteins from isolated lipid droplets [39], sequence identical to UniProt B7G763.	no	no

Protein ID	Name	Location	Method, References, Comments	Contained in reference set of [21]?	Used for statistics?
Phatr2a_48477	Predicted protein	LD	Mass spectrometric peptide mapping of proteins from isolated lipid droplets [39], UniProt ID in publication is B7G7A0, has 770 residues of N-terminal extension (MWTGGKPPPIPSSPSPPGMSPGKFRPKTQD + 740 aa).	no	no
Phatr2a_48499	Coatomer subunit zeta (Fragment)	LD	Mass spectrometric peptide mapping of proteins from isolated lipid droplets [39], UniProt ID in publication is B7G7H3, has 8 residues of N-terminal extension (MSSIPPLL), and 42 positions of C-terminal extension (12 aa + VAPGDMTIGQAFRQAREQFLAGMTSRDGM*).	no	no
Phatr2a_48507	Predicted protein	LD	Mass spectrometric peptide mapping of proteins from isolated lipid droplets [39], sequence identical to UniProt B7G7I0.	no	no
Phatr2a_48548	Predicted protein (Fragment)	LD	Mass spectrometric peptide mapping of proteins from isolated lipid droplets [39], UniProt ID in publication is B7G7M5, has 27 positions of C-terminal extension (GPRDDTDKVGGLLGDMGYKPSQVYKF*).	no	no
Phatr2a_48607	Predicted protein	LD	Mass spectrometric peptide mapping of proteins from isolated lipid droplets [39], sequence identical to UniProt B7G7V1.	no	no
Phatr2a_48636	Glutathione peroxidase	LD	Mass spectrometric peptide mapping of proteins from isolated lipid droplets [39], sequence identical to UniProt B7G7Y3.	no	no
Phatr2a_35080	Peptidylprolyl isomerase (Fragment)	LD	Mass spectrometric peptide mapping of proteins from isolated lipid droplets [39], UniProt ID in publication is B7FXI1, has 23 residues of N-terminal extension (MFMSRLFFSFVVVVVASLSPYAA), and 70 positions of C-terminal extension (40 aa + LQRLQRMVDAKVKPELKLWMERRIHLLQQM).	no	no
Phatr2a_48651	Urate oxidase (Fragment)	LD	Mass spectrometric peptide mapping of proteins from isolated lipid droplets [39], UniProt ID in publication is B7G802, has 162 residues of N-terminal extension (MFSIARRGSALQRTLVALSPSPRTGRASRL + 132 aa), and 31 positions of C-terminal extension (1 aa + RSPLDHAAAQALVQEAKQVAAALDDGVHA*).	no	no
Phatr2a_48661	Predicted protein	LD	Mass spectrometric peptide mapping of proteins from isolated lipid droplets [39], sequence identical to UniProt B7G814.	no	no
Phatr2a_48664	Predicted protein	LD	Mass spectrometric peptide mapping of proteins from isolated lipid droplets [39], sequence identical to UniProt B7G817.	no	no
Phatr2a_48704	Predicted protein	LD	Mass spectrometric peptide mapping of proteins from isolated lipid droplets [39], sequence identical to UniProt B7G7F7.	no	no
Phatr2a_48751	Predicted protein	LD	Mass spectrometric peptide mapping of proteins from isolated lipid droplets [39], sequence identical to UniProt B7G890.	no	no
Phatr2a_48767	Predicted protein	LD	Mass spectrometric peptide mapping of proteins from isolated lipid droplets [39], sequence identical to UniProt B7G8A8.	no	no
Phatr2a_48778	Predicted protein	LD	Mass spectrometric peptide mapping of proteins from isolated lipid droplets [39], sequence identical to UniProt B7G8C2.	no	no

Protein ID	Name	Location	Method, References, Comments	Contained in reference set of [21]?	Used for statistics?
Phatr2a_48822	Predicted protein	LD	Mass spectrometric peptide mapping of proteins from isolated lipid droplets [39], sequence identical to UniProt B7G8H9.	no	no
Phatr2a_48859	Predicted protein	LD	Mass spectrometric peptide mapping of proteins from isolated lipid droplets [39], sequence identical to UniProt B7G8M1.	no	no
Phatr2a_48864	Reductase with NAD or NADP as acceptor	LD	Mass spectrometric peptide mapping of proteins from isolated lipid droplets [39], sequence identical to UniProt B7G8M9.	no	no
Phatr2a_48874	Predicted protein	LD	Mass spectrometric peptide mapping of proteins from isolated lipid droplets [39], UniProt ID in publication is B7G8P1, has 52 residues of N-terminal extension (MHADEAVYGSTRLPHERVPYAVSHTYSSFL + 22 aa).	no	no
Phatr2a_48909	Predicted protein	LD	Mass spectrometric peptide mapping of proteins from isolated lipid droplets [39], sequence identical to UniProt B7G8T7.	no	no
Phatr2a_48920	Predicted protein	LD	Mass spectrometric peptide mapping of proteins from isolated lipid droplets [39], sequence identical to UniProt B7G8V5.	no	no
Phatr2a_48929	Predicted protein	LD	Mass spectrometric peptide mapping of proteins from isolated lipid droplets [39], UniProt ID in publication is B7G8W8, has 61 residues of N-terminal extension (MYLISVWSILFLFTSLPRKEVTTSKSKSIR + 31 aa).	no	no
Phatr2a_48959	Predicted protein	LD	Mass spectrometric peptide mapping of proteins from isolated lipid droplets [39], sequence identical to UniProt B7G909.	no	no
Phatr2a_49037	Predicted protein	LD	Mass spectrometric peptide mapping of proteins from isolated lipid droplets [39], sequence identical to UniProt B7G9A2.	no	no
Phatr2a_49053	ATP synthase subunit d, mitochondrial	LD	Mass spectrometric peptide mapping of proteins from isolated lipid droplets [39], sequence identical to UniProt B7G9C2.	no	no
Phatr2a_49056	Predicted protein	LD	Mass spectrometric peptide mapping of proteins from isolated lipid droplets [39], sequence identical to UniProt B7G9C6.	no	no
Phatr2a_49113	Predicted protein (Fragment)	LD	Mass spectrometric peptide mapping of proteins from isolated lipid droplets [39], UniProt ID in publication is B7G9I9, has 67 residues of N-terminal extension (MCCVVRIRWGWGLQKTGGRDGTFQPWRRLP + 37 aa).	no	no
Phatr2a_49114	CCT-beta	LD	Mass spectrometric peptide mapping of proteins from isolated lipid droplets [39], sequence identical to UniProt B7G9J0.	no	no
Phatr2a_49167	Glutathione reductase	LD	Mass spectrometric peptide mapping of proteins from isolated lipid droplets [39], sequence identical to UniProt B7G9Q1.	no	no
Phatr2a_49185	Predicted protein	LD	Mass spectrometric peptide mapping of proteins from isolated lipid droplets [39], sequence identical to UniProt B7G9S0.	no	no
Phatr2a_49189	Predicted protein	LD	Mass spectrometric peptide mapping of proteins from isolated lipid droplets [39], sequence identical to UniProt B7G9S4.	no	no

Protein ID	Name	Location	Method, References, Comments	Contained in reference set of [21]?	Used for statistics?
Phatr2a_49190	Predicted protein	LD	Mass spectrometric peptide mapping of proteins from isolated lipid droplets [39], sequence identical to UniProt B7G9S5.	no	no
Phatr2a_49216	Predicted protein	LD	Mass spectrometric peptide mapping of proteins from isolated lipid droplets [39], sequence identical to UniProt B7G9V2.	no	no
Phatr2a_49262	Predicted protein	LD	Mass spectrometric peptide mapping of proteins from isolated lipid droplets [39], UniProt ID in publication is B7GA11, has 71 residues of N-terminal extension (MKGSVFLWPSAGQRATHQQHQGTCRRNSSA + 41 aa).	no	no
Phatr2a_49287	Predicted protein	LD	Mass spectrometric peptide mapping of proteins from isolated lipid droplets [39], sequence identical to UniProt B7GA38.	no	no
Phatr2a_49294	Predicted protein (Fragment)	LD	Mass spectrometric peptide mapping of proteins from isolated lipid droplets [39], UniProt ID in publication is B7GA46, has 127 residues of N-terminal extension (MPLLHTAKTWARIAKVFLWLALLTVSGCVA + 97 aa), and 109 positions of C-terminal extension (79 aa + IRSSLNLPSMYYEDLNDIDTDEHKSTPNF*).	no	no
Phatr2a_49301	Predicted protein	LD	Mass spectrometric peptide mapping of proteins from isolated lipid droplets [39], sequence identical to UniProt B7GA54.	no	no
Phatr2a_49499	Inosine-guanosine phosphorylase (Fragment)	LD	Mass spectrometric peptide mapping of proteins from isolated lipid droplets [39], UniProt ID in publication is B7GAT6, has 29 residues of N-terminal extension (MSTMDVSHVPYRLASAYLLTRLESAGLAL), and 64 positions of C-terminal extension (34 aa + ALTVQALLAAGAVFALGCFVGTRMLTARR*).	no	no
Phatr2a_49503	Predicted protein	LD	Mass spectrometric peptide mapping of proteins from isolated lipid droplets [39], UniProt ID in publication is B7GAU1, has 1196 residues of N-terminal extension (MDVAYLVASAGLTSYVRTNVRRVDESQQTY + 1166 aa).	no	no
Phatr2a_49522	Parahox neighbor	LD	Mass spectrometric peptide mapping of proteins from isolated lipid droplets [39], sequence identical to UniProt B7GAW6.	no	no
Phatr2a_49585	Methylenetetrahydrofolate reductase (NAD(P)H) (Fragment)	LD	Mass spectrometric peptide mapping of proteins from isolated lipid droplets [39], UniProt ID in publication is B7GB47, has 79 positions of C-terminal extension (49 aa + AKVEFSQWIMAGGQKPVAYLQKGQYHKVF*).	no	no
Phatr2a_49601	Alanine glyoxylate aminotransferase	LD	Mass spectrometric peptide mapping of proteins from isolated lipid droplets [39], sequence identical to UniProt B7GB64.	no	no
Phatr2a_49707	Predicted protein	LD	Mass spectrometric peptide mapping of proteins from isolated lipid droplets [39], sequence identical to UniProt B7GBM4.	no	no
Phatr2a_49718	Predicted protein	LD	Mass spectrometric peptide mapping of proteins from isolated lipid droplets [39], sequence identical to UniProt B7GBN5.	no	no
Phatr2a_49764	Predicted protein	LD	Mass spectrometric peptide mapping of proteins from isolated lipid droplets [39], sequence identical to UniProt B7GBT9.	no	no

Protein ID	Name	Location	Method, References, Comments	Contained in reference set of [21]?	Used for statistics?
Phatr2a_49782	Predicted protein	LD	Mass spectrometric peptide mapping of proteins from isolated lipid droplets [39], sequence identical to UniProt B7GBV8.	no	no
Phatr2a_49829	Predicted protein	LD	Mass spectrometric peptide mapping of proteins from isolated lipid droplets [39], sequence identical to UniProt B7GBM0.	no	no
Phatr2a_49838	Predicted protein	LD	Mass spectrometric peptide mapping of proteins from isolated lipid droplets [39], sequence identical to UniProt B7GC08.	no	no
Phatr2a_49897	Proteasome endopeptidase complex	LD	Mass spectrometric peptide mapping of proteins from isolated lipid droplets [39], sequence identical to UniProt B7GC84.	no	no
Phatr2a_49924	Predicted protein	LD	Mass spectrometric peptide mapping of proteins from isolated lipid droplets [39], sequence identical to UniProt B7GCB8.	no	no
Phatr2a_50035	Predicted protein	LD	Mass spectrometric peptide mapping of proteins from isolated lipid droplets [39], UniProt ID in publication is B7GCP8, has 157 residues of N-terminal extension (MRYRVTTVQSFLPGGTFAYGTVCRALVFGN + 127 aa).	no	no
Phatr2a_50047	Predicted protein	LD	Mass spectrometric peptide mapping of proteins from isolated lipid droplets [39], sequence identical to UniProt B7GCR2.	no	no
Phatr2a_50049	Predicted protein	LD	Mass spectrometric peptide mapping of proteins from isolated lipid droplets [39], sequence identical to UniProt B7GCR5.	no	no
Phatr2a_50084	Predicted protein	LD	Mass spectrometric peptide mapping of proteins from isolated lipid droplets [39], sequence identical to UniProt B7GCV6.	no	no
Phatr2a_50220	Predicted protein	LD	Mass spectrometric peptide mapping of proteins from isolated lipid droplets [39], sequence identical to UniProt B7FXS3.	no	no
Phatr2a_50236	Predicted protein	LD	Mass spectrometric peptide mapping of proteins from isolated lipid droplets [39], sequence identical to UniProt B7GDE6.	no	no
Phatr2a_50259	Predicted protein	LD	Mass spectrometric peptide mapping of proteins from isolated lipid droplets [39], sequence identical to UniProt B7GDH3.	no	no
Phatr2a_50278	Predicted protein (Fragment)	LD	Mass spectrometric peptide mapping of proteins from isolated lipid droplets [39], UniProt ID in publication is B7GDJ5, has 33 residues of N-terminal extension (MFSHSAQSLATKNLFRARPCLSATVNPYAA + 3 aa), and 42 positions of C-terminal extension (12 aa + SVKVEESSSSGQQKSYEERLAAAGIPFSD*).	no	no
Phatr2a_50316	Predicted protein	LD	Mass spectrometric peptide mapping of proteins from isolated lipid droplets [39], sequence identical to UniProt B7GDP2.	no	no
Phatr2a_50366	Predicted protein	LD	Mass spectrometric peptide mapping of proteins from isolated lipid droplets [39], sequence identical to UniProt B7GDW4.	no	no
Phatr2a_50388	Predicted protein	LD	Mass spectrometric peptide mapping of proteins from isolated lipid droplets [39], sequence identical to UniProt B7GDY7.	no	no

Protein ID	Name	Location	Method, References, Comments	Contained in reference set of [21]?	Used for statistics?
Phatr2a_50441	Predicted protein	LD	Mass spectrometric peptide mapping of proteins from isolated lipid droplets [39], sequence identical to UniProt B7GE47.	no	no
Phatr2a_50448	Predicted protein	LD	Mass spectrometric peptide mapping of proteins from isolated lipid droplets [39], UniProt ID in publication is B7GE54, has 156 residues of N-terminal extension (MTLTVSKTCRSLWVERNPGSMRSATPSCGH + 126 aa).	no	no
Phatr2a_50453	5-proFAR isomerase	LD	Mass spectrometric peptide mapping of proteins from isolated lipid droplets [39], sequence identical to UniProt B7GE60.	no	no
Phatr2a_50499	Predicted protein	LD	Mass spectrometric peptide mapping of proteins from isolated lipid droplets [39], sequence identical to UniProt B7GEA5.	no	no
Phatr2a_50500	Predicted protein	LD	Mass spectrometric peptide mapping of proteins from isolated lipid droplets [39], sequence identical to UniProt B7GEA6.	no	no
Phatr2a_50577	Acetylornithine transaminase	LD	Mass spectrometric peptide mapping of proteins from isolated lipid droplets [39], sequence identical to UniProt B7GEJ6.	no	no
Phatr2a_50588	Predicted protein	LD	Mass spectrometric peptide mapping of proteins from isolated lipid droplets [39], sequence identical to UniProt B7GEL1.	no	no
Phatr2a_50796	Eukaryotic peptide chain release factor subunit 1	LD	Mass spectrometric peptide mapping of proteins from isolated lipid droplets [39], sequence identical to UniProt B7FU01.	no	no
Phatr2a_50886	Predicted protein	LD	Mass spectrometric peptide mapping of proteins from isolated lipid droplets [39], sequence identical to UniProt B7FXK9.	no	no
Phatr2a_51026	Histone deacetylase	LD	Mass spectrometric peptide mapping of proteins from isolated lipid droplets [39], sequence identical to UniProt B7G263.	no	no
Phatr2a_51058	Predicted protein	LD	Mass spectrometric peptide mapping of proteins from isolated lipid droplets [39], sequence identical to UniProt B7G360.	no	no
Phatr2a_51066	Predicted protein	LD	Mass spectrometric peptide mapping of proteins from isolated lipid droplets [39], sequence identical to UniProt B7G3G6.	no	no
Phatr2a_51073	Predicted protein	LD	Mass spectrometric peptide mapping of proteins from isolated lipid droplets [39], sequence identical to UniProt B7G495.	no	no
Phatr2a_51199	Predicted protein	LD	Mass spectrometric peptide mapping of proteins from isolated lipid droplets [39], sequence identical to UniProt B7GA99.	no	no
Phatr2a_51214	Ferredoxin-dependent glutamate synthase, fusion of large and small subunits	LD	Mass spectrometric peptide mapping of proteins from isolated lipid droplets [39], sequence identical to UniProt B7GAZ5.	no	no
Phatr2a_51242	Adenosine kinase	LD	Mass spectrometric peptide mapping of proteins from isolated lipid droplets [39], sequence identical to UniProt B7GCE4.	no	no

Protein ID	Name	Location	Method, References, Comments	Contained in reference set of [21]?	Used for statistics?
Phatr2a_51279	Predicted protein	LD	Mass spectrometric peptide mapping of proteins from isolated lipid droplets [39], sequence identical to UniProt B7GDW6.	no	no
Phatr2a_51291	Predicted protein	LD	Mass spectrometric peptide mapping of proteins from isolated lipid droplets [39], sequence identical to UniProt B7GED8.	no	no
Phatr2a_52215	Predicted protein	LD	Mass spectrometric peptide mapping of proteins from isolated lipid droplets [39], UniProt ID in publication is B7G374, has 2 positions of C-terminal extension (**).	no	no
Phatr2a_52747	Nad-dependent epimerase/dehydratase	LD	Mass spectrometric peptide mapping of proteins from isolated lipid droplets [39], sequence identical to UniProt B7GEG7, N-terminus shared with Phatr2a_56284,31314,41531,53914,24191,24190,31315,55217,31313,50552,24192	no	no
Phatr2a_53998	Predicted protein	LD	Mass spectrometric peptide mapping of proteins from isolated lipid droplets [39], sequence identical to UniProt B7FPU3.	no	no
Phatr2a_54015	Serine hydroxymethyl-transferase	LD	Mass spectrometric peptide mapping of proteins from isolated lipid droplets [39], sequence identical to UniProt B7FQ66.	no	no
Phatr2a_54066	Predicted protein	LD	Mass spectrometric peptide mapping of proteins from isolated lipid droplets [39], sequence identical to UniProt B7FR63.	no	no
Phatr2a_54086	ATP synthase subunit beta	LD	Mass spectrometric peptide mapping of proteins from isolated lipid droplets [39], sequence identical to UniProt B7FS46.	no	no
Phatr2a_54376	Chaperone, dnaj-like protein	LD	Mass spectrometric peptide mapping of proteins from isolated lipid droplets [39], sequence identical to UniProt B7FX28.	no	no
Phatr2a_54381	Histone linker H1	LD	Mass spectrometric peptide mapping of proteins from isolated lipid droplets [39], sequence identical to UniProt B7FX66.	no	no
Phatr2a_54420	Predicted protein	LD	Mass spectrometric peptide mapping of proteins from isolated lipid droplets [39], sequence identical to UniProt B7FXX2.	no	no
Phatr2a_54442	AP-2 complex subunit alpha	LD	Mass spectrometric peptide mapping of proteins from isolated lipid droplets [39], sequence identical to UniProt B7FY75.	no	no
Phatr2a_54476	Formidase	LD	Mass spectrometric peptide mapping of proteins from isolated lipid droplets [39], sequence identical to UniProt B7FYS6.	no	no
Phatr2a_54493	Udp-n-acetylglucosamine diphosphorylase	LD	Mass spectrometric peptide mapping of proteins from isolated lipid droplets [39], sequence identical to UniProt B7FZ25.	no	no
Phatr2a_54511	Coatomer subunit beta	LD	Mass spectrometric peptide mapping of proteins from isolated lipid droplets [39], sequence identical to UniProt B7FZV8.	no	no
Phatr2a_54534	Tubulin alpha chain	LD	Mass spectrometric peptide mapping of proteins from isolated lipid droplets [39], sequence identical to UniProt B7G0C3.	no	no

Protein ID	Name	Location	Method, References, Comments	Contained in reference set of [21]?	Used for statistics?
Phatr2a_54686	Predicted protein	LD	Mass spectrometric peptide mapping of proteins from isolated lipid droplets [39], sequence identical to UniProt B7G2A6.	no	no
Phatr2a_54801	Clathrin heavy chain	LD	Mass spectrometric peptide mapping of proteins from isolated lipid droplets [39], sequence identical to UniProt B7G4Y3.	no	no
Phatr2a_54869	Predicted protein	LD	Mass spectrometric peptide mapping of proteins from isolated lipid droplets [39], sequence identical to UniProt B7G685.	no	no
Phatr2a_54872	Metacaspase	LD	Mass spectrometric peptide mapping of proteins from isolated lipid droplets [39], sequence identical to UniProt B7G6D0.	no	no
Phatr2a_55010	Predicted protein	LD	Mass spectrometric peptide mapping of proteins from isolated lipid droplets [39], sequence identical to UniProt B7G9R3.	no	no
Phatr2a_55035	Pyruvate dehydrogenase E1 component subunit alpha	LD	Mass spectrometric peptide mapping of proteins from isolated lipid droplets [39], sequence identical to UniProt B7GAB0.	no	no
Phatr2a_55206	Macrocin o-methyltransferase domain-containing protein	LD	Mass spectrometric peptide mapping of proteins from isolated lipid droplets [39], sequence identical to UniProt B7GE77.	no	no
Phatr2a_55215	Heat shock protein Hsp90	LD	Mass spectrometric peptide mapping of proteins from isolated lipid droplets [39], sequence identical to UniProt B7GEF7.	no	no
Phatr2a_56284	Nad-dependent epimerase/dehydratase	LD	Mass spectrometric peptide mapping of proteins from isolated lipid droplets [39], sequence identical to UniProt B7GEG7, N-terminus shared with Phatr2a_52747	no	no
Phatr2a_56311	Protein fucoxanthin chlorophyll a/c protein	LD	Mass spectrometric peptide mapping of proteins from isolated lipid droplets [39], UniProt ID in publication is B7FVF9, has 211 residues of N-terminal extension (MKCIAAIALLATTASAFNAFGAAKKAAPKK + 181 aa).	no	no
Phatr2a_56348	Glyceraldehyde-3-phosphate dehydrogenase	LD	Mass spectrometric peptide mapping of proteins from isolated lipid droplets [39], UniProt ID in publication is B7G6K6	no	no
Phatr2a_56355	Predicted protein	LD	Mass spectrometric peptide mapping of proteins from isolated lipid droplets [39], UniProt ID in publication is B7GES3, has 710 residues of N-terminal extension (MSAADTESYAFSADINQLLSLIINTFYSNK + 680 aa).	no	no
Phatr2a_56356	Glutamate dehydrogenase	LD	Mass spectrometric peptide mapping of proteins from isolated lipid droplets [39], UniProt ID in publication is B7G3X3, has 643 residues of N-terminal extension (MSARSEAPTIFKSRNGDPTQKIDLTIQKKN + 613 aa).	no	no
Phatr2a_56366	Protein heat shock protein Hsp70	LD	Mass spectrometric peptide mapping of proteins from isolated lipid droplets [39], UniProt ID in publication is B7FQ84, has 654 residues of N-terminal extension (MSVTGESVGIDLGTTYSCVGVWQNDRVEII + 624 aa).	no	no

Protein ID	Name	Location	Method, References, Comments	Contained in reference set of [21]?	Used for statistics?
Phatr2a_56389	Pyrophosphate–fructose 6-phosphate 1-phosphotransferase	LD	Mass spectrometric peptide mapping of proteins from isolated lipid droplets [39], UniProt ID in publication is B7GCG9, has 419 residues of N-terminal extension (MSHQANVLKGTHVNVAMMTSGGLAPCLSSS + 389 aa).	no	no
Phatr2a_56395	Calmodulin	LD	Mass spectrometric peptide mapping of proteins from isolated lipid droplets [39], UniProt ID in publication is B7G8H4, has 488 residues of N-terminal extension (MKSFSLLSILTASLAPAITDALKPSKCGGS + 458 aa).	no	no
Phatr2a_56421	Predicted protein	LD	Mass spectrometric peptide mapping of proteins from isolated lipid droplets [39], UniProt ID in publication is B7GED7, has 689 residues of N-terminal extension (MTDMISKGPSLVFGASGEQGRVVVEGLVDT + 659 aa).	no	no
Phatr2a_56424	Annexin	LD	Mass spectrometric peptide mapping of proteins from isolated lipid droplets [39], UniProt ID in publication is B5Y5D2, has 334 residues of N-terminal extension (MTIPIYPGIVKENDLSPDQGFGSEIDDLCQ + 304 aa).	no	no
Phatr2a_56428	Predicted protein	LD	Mass spectrometric peptide mapping of proteins from isolated lipid droplets [39], UniProt ID in publication is B7FSZ7, has 361 residues of N-terminal extension (MWRIPSITLLFLTASGATRVESEASLSVEL + 331 aa).	no	no
Phatr2a_56468	Phosphopyruvate hydratase	LD	Mass spectrometric peptide mapping of proteins from isolated lipid droplets [39], UniProt ID in publication is B7GEF2, has 484 residues of N-terminal extension (MLFKPSTLLALFAVAGTTLAFAPRSTTTSL + 454 aa).	no	no
Phatr2a_56628	AIR carboxylase	LD	Mass spectrometric peptide mapping of proteins from isolated lipid droplets [39], UniProt ID in publication is B7FS21, has 1330 residues of N-terminal extension (MTADAKPSLPATEESPAETELRSRLARTIQ + 1300 aa).	no	no
Phatr2a_56630	SAICAR synthetase	LD	Mass spectrometric peptide mapping of proteins from isolated lipid droplets [39], sequence identical to UniProt B7FS23.	no	no
Phatr2a_56729	Predicted protein	LD	Mass spectrometric peptide mapping of proteins from isolated lipid droplets [39], UniProt ID in publication is B7FUE7, has 55 residues of N-terminal extension (MWAHRRASRSYKTVAGLWNAASFLRAKKFP + 25 aa).	no	no
Phatr2a_8659	Predicted protein	LD	Mass spectrometric peptide mapping of proteins from isolated lipid droplets [39], sequence identical to UniProt B7FPR2.	no	no
Phatr2a_8755	Asparagine–tRNA ligase	LD	Mass spectrometric peptide mapping of proteins from isolated lipid droplets [39], sequence identical to UniProt B7FP41.	no	no
Phatr2u_1252	Predicted protein	LD	Mass spectrometric peptide mapping of proteins from isolated lipid droplets [39], sequence identical to UniProt B7S3S6.	no	no
Phatr2u_1316	Predicted protein	LD	Mass spectrometric peptide mapping of proteins from isolated lipid droplets [39], sequence identical to UniProt B7S413.	no	no
Phatr2u_1325	Diphosphomevalonate decarboxylase	LD	Mass spectrometric peptide mapping of proteins from isolated lipid droplets [39], sequence identical to UniProt B7S422.	no	no

Protein ID	Name	Location	Method, References, Comments	Contained in reference set of [21]?	Used for statistics?
Phatr2u_1469	2-hydroxyacid dehydrogenase	LD	Mass spectrometric peptide mapping of proteins from isolated lipid droplets [39], sequence identical to UniProt B7S4E4.	no	no
Phatr2u_1471	Predicted protein (Fragment)	LD	Mass spectrometric peptide mapping of proteins from isolated lipid droplets [39], sequence identical to UniProt B7S4E5.	no	no
Phatr2u_1626	Predicted protein	LD	Mass spectrometric peptide mapping of proteins from isolated lipid droplets [39], sequence identical to UniProt B7S3W9.	no	no
Phatr2u_1638	Predicted protein	LD	Mass spectrometric peptide mapping of proteins from isolated lipid droplets [39], sequence identical to UniProt B7S3Y6.	no	no
Phatr2u_1648	Peptidylprolyl isomerase	LD	Mass spectrometric peptide mapping of proteins from isolated lipid droplets [39], sequence identical to UniProt B7S404.	no	no
Phatr2u_1649	Predicted protein	LD	Mass spectrometric peptide mapping of proteins from isolated lipid droplets [39], sequence identical to UniProt B7S406.	no	no
Phatr2u_1704	Predicted protein	LD	Mass spectrometric peptide mapping of proteins from isolated lipid droplets [39], sequence identical to UniProt B7S480.	no	no
Phatr2u_1735	Predicted protein	LD	Mass spectrometric peptide mapping of proteins from isolated lipid droplets [39], sequence identical to UniProt B7S4C6.	no	no
Phatr2u_176	Predicted protein (Fragment)	LD	Mass spectrometric peptide mapping of proteins from isolated lipid droplets [39], sequence identical to UniProt B7S423.	no	no
Phatr2u_1820	Alcohol dehydrogenase	LD	Mass spectrometric peptide mapping of proteins from isolated lipid droplets [39], sequence identical to UniProt B7S4B2.	no	no
Phatr2u_289	Predicted protein (Fragment)	LD	Mass spectrometric peptide mapping of proteins from isolated lipid droplets [39], sequence identical to UniProt B7S3J3.	no	no
Phatr2u_348	Acetohydroxy-acid reductoisomerase	LD	Mass spectrometric peptide mapping of proteins from isolated lipid droplets [39], sequence identical to UniProt B7S3L8.	no	no
Phatr2u_36	alpha-1,2-Mannosidase (Fragment)	LD	Mass spectrometric peptide mapping of proteins from isolated lipid droplets [39], sequence identical to UniProt B7S490.	no	no
Phatr2u_477	Predicted protein	LD	Mass spectrometric peptide mapping of proteins from isolated lipid droplets [39], sequence identical to UniProt B7S416.	no	no
Phatr2u_542	Cysteine synthase	LD	Mass spectrometric peptide mapping of proteins from isolated lipid droplets [39], sequence identical to UniProt B7S487.	no	no
Phatr2u_552	Arginyl-tRNA synthetase (Fragment)	LD	Mass spectrometric peptide mapping of proteins from isolated lipid droplets [39], sequence identical to UniProt B7S469.	no	no
Phatr2u_611	Proteasome endopeptidase complex	LD	Mass spectrometric peptide mapping of proteins from isolated lipid droplets [39], sequence identical to UniProt B7S4L0.	no	no

Protein ID	Name	Location	Method, References, Comments	Contained in reference set of [21]?	Used for statistics?
Phatr2u_741	Predicted protein	LD	Mass spectrometric peptide mapping of proteins from isolated lipid droplets [39], sequence identical to UniProt B7S483.	no	no
Phatr2u_913	S-adenosylmethionine synthase	LD	Mass spectrometric peptide mapping of proteins from isolated lipid droplets [39], sequence identical to UniProt B7S466.	no	no
Phatr2u_998	Predicted protein	LD	Mass spectrometric peptide mapping of proteins from isolated lipid droplets [39], sequence identical to UniProt B7S3S7.	no	no

References

[1] Allen AE, Dupont CL, Oborník M, Horák A, Nunes-Nesi A, et al. (2011) Evolution and metabolic significance of the urea cycle in photosynthetic diatoms. Nature 473: 203–207.

[2] Allen AE, Moustafa A, Montsant A, Eckert A, Kroth PG, Bowler C (2012) Evolution and Functional Diversification of Fructose Bisphosphate Aldolase Genes in Photosynthetic Marine Diatoms. Molecular Biology and Evolution 29: 367–379.

[3] Apt KE, Zaslavkaia L, Lippmeier JC, Lang M, Kilian O, et al. (2002) *In vivo* characterization of diatom multipartite plastid targeting signals. J Cell Sci 115: 4061–4069.

[4] Ast M, Gruber A, Schmitz-Esser S, Neuhaus HE, Kroth PG, et al. (2009) Diatom plastids depend on nucleotide import from the cytosol. Proc Natl Acad Sci USA 106: 3621–3626.

[5] Balamurugan S, Wang X, Wang HL, An CJ, Li H, et al. (2017) Occurrence of plastidial triacylglycerol synthesis and the potential regulatory role of AGPAT in the model diatom *Phaeodactylum tricornutum*. Biotechnology for Biofuels 10: 97.

[6] Bhaya D, Grossman AR (1993) Characterization of gene clusters encoding the fucoxanthin chlorophyll proteins of the diatom *Phaeodactylum tricornutum*. Nucleic Acids Research 21: 4458–4466.

[7] Bowler C, Allen AE, Badger JH, Grimwood J, Jabbari K, et al. (2008) The *Phaeodactylum* genome reveals the evolutionary history of diatom genomes. Nature 456: 239–244.

[8] Bruckner CG, Rehm C, Grossart HP, Kroth PG (2011) Growth and release of extracellular organic compounds by benthic diatoms depend on interactions with bacteria. Environmental Microbiology 13: 1052–1063.

[9] Buhmann MT, Schulze B, Förderer A, Schleheck D, Kroth PG (2016) Bacteria may induce the secretion of mucin-like proteins by the diatom Phaeodactylum tricornutum. Journal of Phycology 52: 463–474.

[10] Bullmann L, Haarmann R, Mirus O, Bredemeier R, Hempel F, et al. (2010) Filling the Gap, Evolutionarily Conserved Omp85 in Plastids of Chromalveolates. Journal of Biological Chemistry 285: 6848–6856.

[11] Burmeister C (2009). Lokalisation möglicher periplastidärer Proteine in der Diatomee *Phaeodactylum tricornutum* (in German). Bachelorarbeit, Universität Konstanz.

[12] Chen Z, Luo L, Chen R, Hu H, Pan Y, et al. (2018) Acetylome Profiling Reveals Extensive Lysine Acetylation of the Fatty Acid Metabolism Pathway in the Diatom *Phaeodactylum tricornutum*. Molecular & Cellular Proteomics 17: 399–412.

[13] Domergue F, Spiekermann P, Lerchl J, Beckmann C, Kilian O, et al. (2003) New insight into *Phaeodactylum tricornutum* fatty acid metabolism. Cloning and functional characterization of plastidial and microsomal delta12-fatty acid desaturases. Plant Physiol 131: 1648–1660.

[14] Erdene-Ochir E, Shin BK, Kwon B, Jung C, Pan CH (2019) Identification and characterisation of the novel endogenous promoter HASP1 and its signal peptide from Phaeodactylum tricornutum. Scientific Reports 9: 9941.

[15] Ewe D, Tachibana M, Kikutani S, Gruber A, Bartulos CR, et al. (2018) The intracellular distribution of inorganic carbon fixing enzymes does not support the presence of a C4 pathway in the diatom Phaeodactylum tricornutum. Photosynthesis Research 137: 263–280.

[16] Felsner G, Sommer M, Maier U (2010) The physical and functional borders of transit peptide-like sequences in secondary endosymbionts. BMC Plant Biology 10: 223.

[17] Gile GH, Moog D, Slamovits CH, Maier UG, Archibald JM (2015) Dual Organellar Targeting of Aminoacyl-tRNA Synthetases in Diatoms and Cryptophytes. Genome biology and evolution 7: 1728–1742.

[18] Gonzalez NH, Felsner G, Schramm FD, Klingl A, Maier UG, Bolte K (2011) A single peroxisomal targeting signal mediates matrix protein import in diatoms. PLoS One 6: e25316.

[19] Gould SB, Sommer MS, Kroth PG, Gile GH, Keeling PJ, Maier UG (2006) Nucleus-to-Nucleus Gene Transfer and Protein Retargeting into a Remnant Cytoplasm of Cryptophytes and Diatoms. Mol Biol Evol 23: 2413–2422.

[20] Grouneva I, Rokka A, Aro EM (2011) The Thylakoid Membrane Proteome of Two Marine Diatoms Outlines Both Diatom-Specific and Species-Specific Features of the Photosynthetic Machinery. Journal of Proteome Research 10: 5338–5353.

[21] Gruber A, Rocap G, Kroth PG, Armbrust EV, Mock T (2015) Plastid proteome prediction for diatoms and other algae with secondary plastids of the red lineage. Plant Journal 81: 519–528.

[22] Gruber A, Vugrinec S, Hempel F, Gould S, Maier UG, Kroth P (2007) Protein targeting into complex diatom plastids: functional characterisation of a specific targeting motif. Plant Mol Biol 64: 519–530.

[23] Gruber A, Weber T, Río Bártulos C, Vugrinec S, Kroth PG (2009) Intracellular distribution of the reductive and oxidative pentose phosphate pathways in two diatoms. Journal of Basic Microbiology 49: 58–72.

[24] Hao X, Luo L, Jouhet J, Rébeillé F, Maréchal E, et al. (2018) Enhanced triacylglycerol production in the diatom *Phaeodactylum tricornutum* by inactivation of a Hotdog-fold thioesterase gene using TALEN-based targeted mutagenesis. Biotechnology for Biofuels 11: 312.

[25] Hempel F, Bullmann L, Lau J, Zauner S, Maier UG (2009) ERAD-Derived Preprotein Transport across the Second Outermost Plastid Membrane of Diatoms. Molecular Biology and Evolution 26: 1781–1790.

[26] Hempel F, Felsner G, Maier UG (2010) New mechanistic insights into preprotein transport across the second outermost plastid membrane of diatoms. Molecular Microbiology 76: 793–801.

[27] Huang W, Haferkamp I, Lepetit B, Molchanova M, Hou S, et al. (2018) Reduced vacuolar beta-1,3-glucan synthesis affects carbohydrate metabolism as well as plastid homeostasis and structure in *Phaeodactylum tricornutum*. Proceedings of the National Academy of Sciences 115: 4791–4796.

[28] Huang W, Río Bártulos C, Kroth PG (2016) Diatom Vacuolar 1,6-beta-Transglycosylases can Functionally Complement the Respective Yeast Mutants. Journal of Eukaryotic Microbiology 63: 536–546.

[29] Jallet D, Xing D, Hughes A, Moosburner M, Simmons MP, et al. (2020) Mitochondrial fatty acid beta-oxidation is required for storage-lipid catabolism in a marine diatom. New Phytologist 228: 946–958.

[30] Joshi-Deo J, Schmidt M, Gruber A, Weisheit W, Mittag M, et al. (2010) Characterization of a trimeric light-harvesting complex in the diatom *Phaeodactylum tricornutum* built of FcpA and FcpE proteins. Journal of Experimental Botany 61: 3079–3087.

[31] Kilian O, Kroth PG (2004) Presequence acquisition during secondary endocytobiosis and the possible role of introns. J Mol Evol 58: 712–721.

[32] Kilian O, Kroth PG (2005) Identification and characterization of a new conserved motif within the presequence of proteins targeted into complex diatom plastids. Plant J 41: 175–183.

[33] Kitao Y, Harada H, Matsuda Y (2008) Localization and targeting mechanisms of two chloroplastic β-carbonic anhydrases in the marine diatom *Phaeodactylum tricornutum*. Physiologia Plantarum 133: 68–77.

[34] Kitao Y, Matsuda Y (2009) Formation of macromolecular complexes of carbonic anhydrases in the chloroplast of a marine diatom by the action of the C-terminal helix. Biochem J 419: 681–688.

[35] Kroth PG, Schroers Y, Kilian O (2005) The peculiar distribution of class I and class II aldolases in diatoms and in red algae. Curr Genet 48: 389–400.

[36] Lau JB, Stork S, Moog D, Schulz J, Maier UG (2016) Protein–protein interactions indicate composition of a 480 kDa SELMA complex in the second outermost membrane of diatom complex plastids. Molecular Microbiology 100: 76–89.

[37] Lau JB, Stork S, Moog D, Sommer MS, Maier UG (2015) N-terminal lysines are essential for protein translocation via a modified ERAD system in complex plastids. Molecular Microbiology 96: 609–620.

[38] Lepetit B, Volke D, Szabó M, Hoffmann R, Garab G, et al. (2007) Spectroscopic and Molecular Characterization of the Oligomeric Antenna of the Diatom *Phaeodactylum tricornutum*. Biochemistry 46: 9813–9822.

[39] Leyland B, Zarka A, Didi-Cohen S, Boussiba S, Khozin-Goldberg I High resolution proteome of lipid droplets isolated from the pennate diatom *Phaeodactylum tricornutum* (Bacillariophyceae) strain pt4 provides mechanistic insights into complex intracellular coordination during nitrogen deprivation. Journal of Phycology 56: 1642–1663.

[40] Liaud M, Lichtle C, Apt K, Martin W, Cerff R (2000) Compartment-specific isoforms of TPI and GAPDH are imported into diatom mitochondria as a fusion protein: Evidence in favor of a mitochondrial origin of the eukaryotic glycolytic pathway. Mol Biol Evol 17: 213–223.

[41] Liu X, Hempel F, Stork S, Bolte K, Moog D, et al. (2016) Addressing various compartments of the diatom model organism *Phaeodactylum tricornutum* via sub-cellular marker proteins. Algal Research 20: 249–257.

[42] Marter P, Schmidt S, Kiontke S, Moog D (2020) Optimized mRuby3 is a Suitable Fluorescent Protein for in vivo Co-localization Studies with GFP in the Diatom *Phaeodactylum tricornutum.* Protist 171: 125715.

[43] Materna A (2006). Development of molecular tools in the diatom *Phaeodactylum tricornutum.* Dissertation, Universität Konstanz.

[44] Mix AK, Cenci U, Heimerl T, Marter P, Wirkner ML, Moog D (2018) Identification and Localization of Peroxisomal Biogenesis Proteins Indicates the Presence of Peroxisomes in the Cryptophyte *Guillardia theta* and Other "Chromalveolates". Genome biology and evolution 10: 2834–2852.

[45] Montsant A, Maheswari U, Bowler C, Lopez PJ (2005) Diatomics: Toward diatom functional genomics. Journal of Nanoscience and Nanotechnology 5: 5–14.

[46] Moog D, Rensing SA, Archibald JM, Maier UG, Ullrich KK (2015) Localization and evolution of putative triose phosphate translocators in the diatom *Phaeodactylum tricornutum.* Genome Biology and Evolution 7: 2955–2969.

[47] Moog D, Stork S, Zauner S, Maier UG (2011) In Silico and In Vivo Investigations of Proteins of a Minimized Eukaryotic Cytoplasm. Genome Biology and Evolution 3: 375–382.

[48] Niu YF, Wang X, Hu DX, Balamurugan S, Li DW, et al. (2016) Molecular characterization of a glycerol-3-phosphate acyltransferase reveals key features essential for triacylglycerol production in Phaeodactylum tricornutum. Biotechnology for Biofuels 9: 60.

[49] Peschke M, Moog D, Klingl A, Maier UG, Hempel F (2013) Evidence for glycoprotein transport into complex plastids. Proceedings of the National Academy of Sciences of the United States of America 110: 10860–10865.

[50] Rio Bartulos C, Rogers MB, Williams TA, Gentekaki E, Brinkmann H, et al. (2018) Mitochondrial Glycolysis in a Major Lineage of Eukaryotes. Genome biology and evolution 10: 2310–2325.

[51] Schellenberger Costa B, Sachse M, Jungandreas A, Bartulos CR, Gruber A, et al. (2013) Aureochrome 1a is involved in the photoacclimation of the diatom *Phaeodactylum tricornutum.* PLoS One 8: e74451.

[52] Schreiber V, Dersch J, Puzik K, Bäcker O, Liu X, et al. (2017) The Central Vacuole of the Diatom *Phaeodactylum tricornutum*: Identification of New Vacuolar Membrane Proteins and of a Functional Di-leucine-based Targeting Motif. Protist 168: 271–282.

[53] Seo S, Kim J, Lee JW, Nam O, Chang KS, Jin E (2020) Enhanced pyruvate metabolism in plastids by overexpression of putative plastidial pyruvate transporter in *Phaeodactylum tricornutum.* Biotechnology for Biofuels 13: 86.

[54] Shao Z, Thomas Y, Hembach L, Xing X, Duan D, et al. (2019) Comparative characterization of putative chitin deacetylases from Phaeodactylum tricornutum and Thalassiosira pseudonana highlights the potential for distinct chitin-based metabolic processes in diatoms. New Phytologist 221: 1890–1905.

[55] Siaut M, Heijde M, Mangogna M, Montsant A, Coesel S, et al. (2007) Molecular toolbox for studying diatom biology in *Phaeodactylum tricornutum.* Gene 406: 23–35.

[56] Sommer MS, Gould SB, Lehmann P, Gruber A, Przyborski JM, Maier UG (2007) Der1-mediated Preprotein Import into the Periplastid Compartment of Chromalveolates? Mol Biol Evol 24: 918–928.

[57] Stork S, Moog D, Przyborski JM, Wilhelmi I, Zauner S, Maier UG (2012) Distribution of the SELMA Translocon in Secondary Plastids of Red Algal Origin and Predicted Uncoupling of Ubiquitin-Dependent Translocation from Degradation. Eukaryotic Cell 11: 1472–1481.

[58] Sturm S, Engelken J, Gruber A, Vugrinec S, Kroth PG, et al. (2013) A novel type of light-harvesting antenna protein of red algal origin in algae with secondary plastids. BMC Evolutionary Biology 13: 159.

[59] Tachibana M, Allen AE, Kikutani S, Endo Y, Bowler C, Matsuda Y (2011) Localization of putative carbonic anhydrases in two marine diatoms, *Phaeodactylum tricornutum* and *Thalassiosira pseudonana.* Photosynthesis Research 109: 205–221.

[60] Tanaka Y, Nakatsuma D, Harada H, Ishida M, Matsuda Y (2005) Localization of Soluble β-Carbonic Anhydrase in the Marine Diatom *Phaeodactylum tricornutum.* Sorting to the Chloroplast and Cluster Formation on the Girdle Lamellae. Plant Physiology 138: 207–217.

[61] Vugrinec S, Gruber A, Kroth PG (2011) Protein targeting into complex plastids—support for the translocator model. Endocytobiosis and Cell Research 21: 59–63.

[62] Wang X, Dong HP, Wei W, Balamurugan S, Yang WD, et al. (2018) Dual expression of plastidial GPAT1 and LPAT1 regulates triacylglycerol production and the fatty acid profile in *Phaeodactylum tricornutum*. Biotechnology for Biofuels 11: 318.

[63] Weber T, Gruber A, Kroth PG (2009) The Presence and Localization of Thioredoxins in Diatoms, Unicellular Algae of Secondary Endosymbiotic Origin. Molecular Plant 2: 468–477.
