# Supplementary material for: ASAFind 2.0: multi‐class protein targeting prediction for diatoms and algae with complex plastids
Source: Plant J. 2025 Jun 4;122(5):e70138. doi: 10.1111/tpj.70138 (PMC12136025; doi:10.1111/tpj.70138)
Supplement: Supplementary file 11 — Appendix S11. (a) Overlaps of Phaeodactylum tricornutum PPC protein predictions (Appendix S6) with experimental data (Appendix S4). Direct sequence identity (“Proteins”) and KEGG category representations (“K numbers”) are shown. (b) Annotated KEGG categories of predicted PPC proteins for Phaeodactylum tricornutum (“Phatr”) and Thalassiosira pseudonana (“Thaps”), raw data in Appendices S6 and S7. (c) Prediction results for experimentally determined organelle proteins; proteome data from Schober et al. (2019), prediction data from Appendix S7. [file TPJ-122-0-s003.pdf]

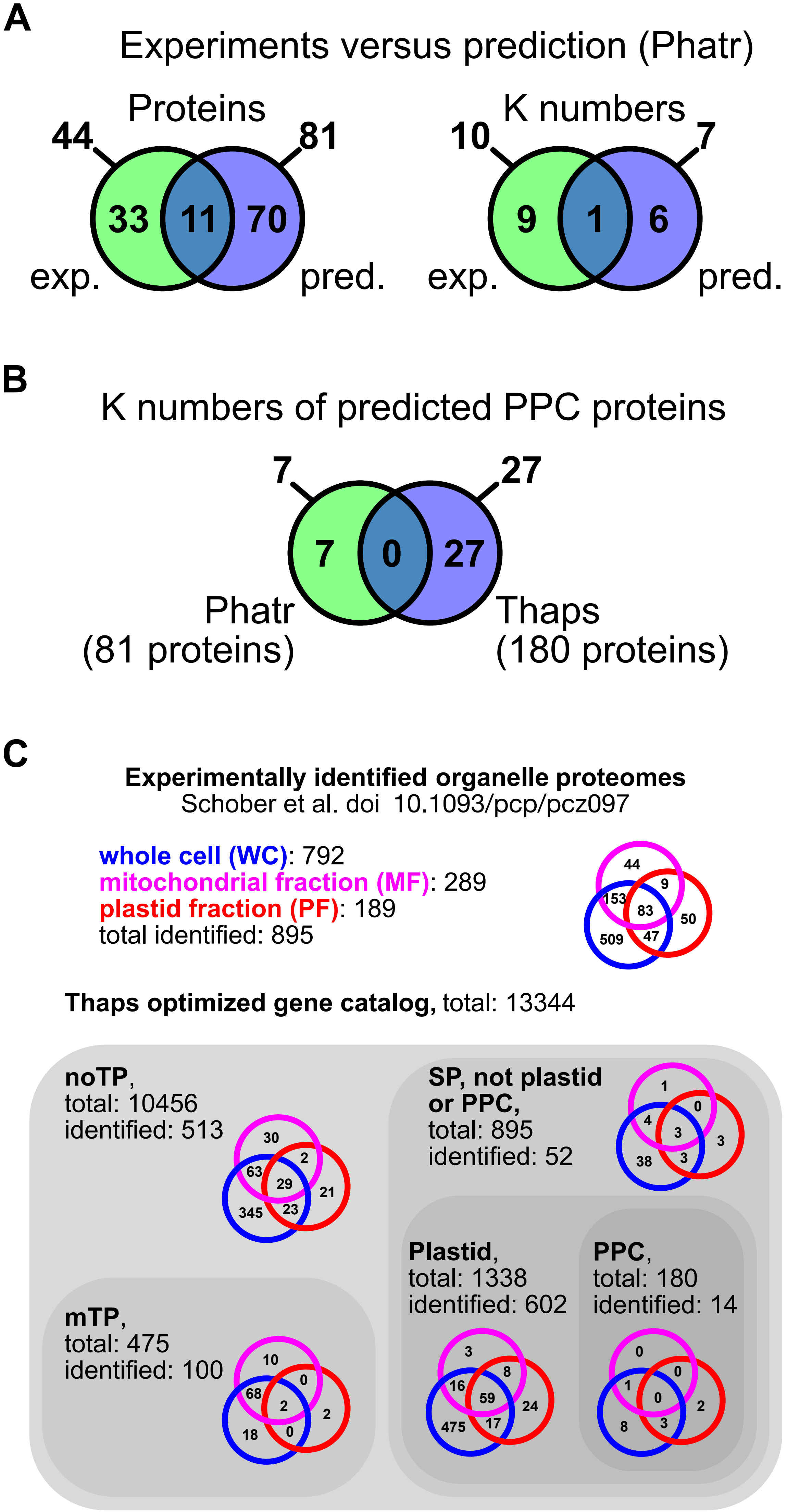
A
Experiments versus prediction (Phatr)
Proteins
44
81
33
11
70
exp.
pred.
K numbers
10
7
9
1
6
exp.
pred.
B
K numbers of predicted PPC proteins
7
27
7
0
27
Phatr
(81 proteins)
Thaps
(180 proteins)
C
Experimentally identified organelle proteomes
Schober et al. doi 10.1093/pcp/pcz097
whole cell (WC): 792
mitochondrial fraction (MF): 289
plastid fraction (PF): 189
total identified: 895
44
9
153
83
50
509
47
Thaps optimized gene catalog, total: 13344
noTP,
total: 10456
identified: 513
30
2
63
29
21
345
23
SP, not plastid or PPC,
total: 895
identified: 52
1
0
4
3
3
38
3
mTP,
total: 475
identified: 100
10
0
68
2
2
18
0
Plastid,
total: 1338
identified: 602
3
8
16
59
24
475
17
PPC,
total: 180
identified: 14
0
0
1
0
2
8
3
